# Practical one-pot amidation of *N*-Alloc-, *N*-Boc-, and *N*-Cbz protected amines under mild conditions†

**DOI:** 10.1039/d1ra02242c

**Published:** 2021-04-28

**Authors:** Wan Pyo Hong, Van Hieu Tran, Hee-Kwon Kim

**Affiliations:** School of Advanced Materials and Chemical Engineering, Daegu Catholic University 13-13, Hayang-ro, Hayang-eup Gyeongsan-si Gyeongbuk 38430 Republic of Korea; Department of Nuclear Medicine, Molecular Imaging & Therapeutic Medicine Research Center, Jeonbuk National University Medical School and Hospital Jeonju 54907 Republic of Korea hkkim717@jbnu.ac.kr; Research Institute of Clinical Medicine of Jeonbuk National University-Biomedical Research Institute of Jeonbuk National University Hospital Jeonju 54907 Republic of Korea

## Abstract

A facile one-pot synthesis of amides from *N*-Alloc-, *N*-Boc-, and *N*-Cbz-protected amines has been described. The reactions involve the use of isocyanate intermediates, which are generated *in situ* in the presence of 2-chloropyridine and trifluoromethanesulfonyl anhydride, to react with Grignard reagents to produce the corresponding amides. Using this reaction protocol, a variety of *N*-Alloc-, *N*-Boc-, and *N*-Cbz-protected aliphatic amines and aryl amines were efficiently converted to amides with high yields. This method is highly effective for the synthesis of amides and offers a promising approach for facile amidation.

## Abstract 2

One-pot efficient transformation of *N*-Alloc-, *N*-Boc-, and *N*-Cbz protected amines to amides was achieved by using 2-chloropyridine and trifluoromethanesulfonyl anhydride as well as Grignard reagent and MgCl_2_.

## Graphical abstract

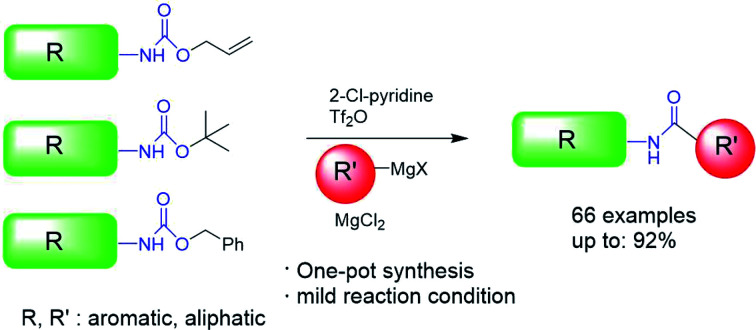

## Introduction

Amide functional groups are important in nature, as they provide the main amino acid linkage in peptides and proteins.^1,2^ In addition, amide structures have been frequently found in many natural products and biologically active compounds.^3–7^ Moreover, numerous drugs including anticancer agents, antibiotics, anesthetics, and enzyme inhibitors contain an amide bond moiety.^8–12^

Due to the importance of amide structures, development of a novel efficient amide formation procedure is a highly attractive area of research, and numerous synthetic methods for the preparation of amides have been developed.^13^ One of the commonly used synthetic methods for preparation of amides is condensation of carboxylic acids with amines.^14,15^ The other useful traditional method for preparation of amides is acylation of amines using acid chlorides.^16,17^ Carbodiimide-mediated amidation also is a popular method.^18,19^ In addition, various synthetic procedures using acyl azide and anhydrides, Staudinger ligation, and the Schmidt reaction have been used to produce amides.^15–24^

Amines are common functional groups in chemistry. In a variety of multi-step organic syntheses, amines are employed with protecting groups to reduce the production of undesired side products. Notably, allyl-carbamate (Alloc-carbamate), *tert*-butyl-carbamate (Boc-carbamate), and benzyl-carbamate (Cbz-carbamate) are observed in organic synthetic processes,^25–28^ because *N*-Alloc-, *N*-Boc-, and *N*-Cbz-protected amines are easily synthesized from various amines using many methods.

However, preparation of amides from these protected amines generally requires two reaction steps: removal of the protecting group from amines to produce free amines, followed by reactions of amines with carboxylic acids. Thus, development of direct efficient preparation of amides from protected amines is important in organic synthesis to reduce cost, waste, and time.

Unfortunately, direct synthesis of amides from Alloc-carbamate, Boc-carbamate, or Cbz-carbamate has not been extensively studied. Only one method has been reported and involves coupling reaction of arylboroxines and carbamates (Boc-carbamate or Cbz-carbamate).^29^ However, formation of amides required high reaction temperatures (100 °C). Moreover, a long process time (16 h) was required to complete the reaction.

**Scheme 1:**
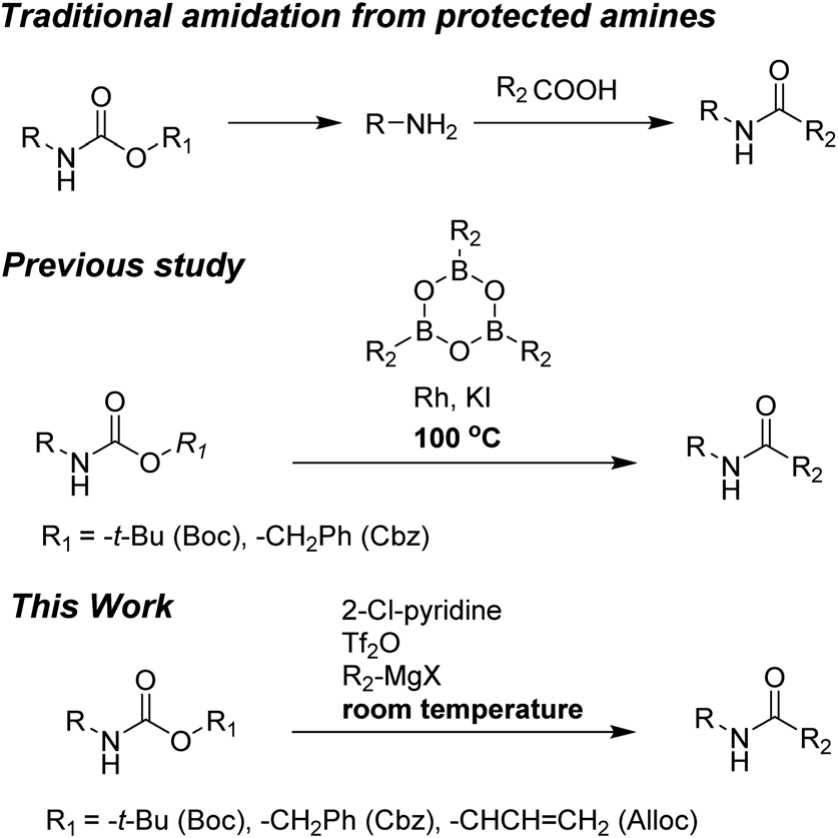
Synthetic approach to amidation of *N*-protected amines.

To the best of our knowledge, simple and facile direct synthesis of amides from Alloc-carbamate, Boc-carbamate, or Cbz-carbamate under mild reaction conditions with short reaction time has not been reported. Thus, development of a novel, effective, and rapid synthetic method under mild conditions for amide formation is a valuable challenge. Herein, we present a novel direct synthetic method for various amides from *N*-Alloc, *N*-Boc-, and *N*-Cbz-protected amines, which is readily applicable in general organic chemistry (Scheme 1).

## Results and discussion

We hypothesized that additional active intermediates generated from protected amines could be used to generate amides directly. Particularly, in this study, we expect that protected amines could be converted into isocyanate intermediates *via* the combined reaction system using bases and trifluoromethanesulfonyl anhydride, followed by treatment with Grignard reagents that could provide target amides under mild conditions. For realization of our idea, *N*-Alloc-protected aniline was selected as the model substrate, because it is widely used in many organic chemistry applications, and trifluoromethanesulfonyl anhydride and phenylmagnesium bromide were employed to produce the amide. In the initial optimization study, the synthetic yield of the target amides was evaluated after reaction with phenylmagnesium bromide at room temperature for 30 min.

We first attempted the reaction experiments with the bases trimethylamine, K_2_CO_3_, DBU, and DMAP, but an amidated product was not obtained (Table 1, entries 1–4). We also examined pyridine as a base; however, the amidated product was prepared in low yield (Table 1, entry 5). Several efficient reactions using both 2-halopyridine and trifluoromethanesulfonyl anhydride have been reported.^30,31^ Thus, 2-halopyridine was tested as a base for the amide formation reaction, and the corresponding amide was obtained with an enhanced yield. When 2-chloropyridine (2-Cl-pyrine) was used, the desired amide was produced in 58% yield (Table 1, entry 6). Addition of 2-bromopyridine (2-Br-pyridine) afforded the corresponding product at 52% yield (Table 1, entry 7). Several previous studies said that utilization of 2-halopyridine and trifluoromethanesulfonyl anhydride could prepare activated intermediates which provided regioselective reactions due to chloro group at the 2-position of pyridinium ring.^32–34^ Thus, it can be assumed that, in this study, employment of 2-chloropyridine and trifluoromethanesulfonyl anhydride gave high reaction yield than those of reactions using the other bases. In addition, 2-methylpyridine (2-Me-pyridine), another pyridine analogue, was evaluated but produced the target in low yield (27%) (Table 1, entry 8).

**Table 1:** Screening of reaction conditions for one-pot amidation^a^

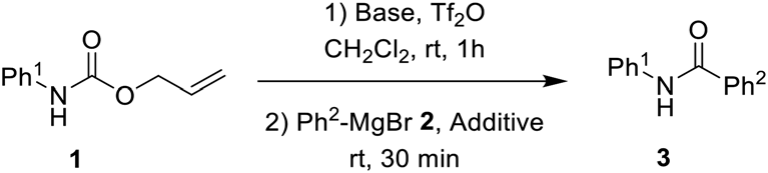				
Entry	Base (equiv.)	Tf_2_O (equiv.)	Additive (equiv.)	Yield^b^ (%)
1	Et_3_N (2.0)	1.3	—	NR^c^
2	K_2_CO_3_ (2.0)	1.3	—	NR^c^
3	DBU (2.0)	1.3	—	NR^c^
4	DMAP (2.0)	1.3	—	NR^c^
5	Pyridine (2.0)	1.3	—	21
6	2-Cl-pyridine (2.0)	1.3	—	58
7	2-Br-pyridine (3.0)	1.3	—	52
8	2-Me-pyridine (2.0)	1.3	—	27
9	2-Cl-pyridine (1.0)	1.3	—	35
10	2-Cl-pyridine (3.0)	1.3	—	58
11	2-Cl-pyridine (4.0)	1.3	—	58
12	2-Cl-pyridine (2.0)	1.0	—	49
13	2-Cl-pyridine (2.0)	2.0	—	58
14	2-Cl-pyridine (2.0)	3.0	—	58
15	2-Cl-pyridine (2.0)	1.3	BiCl_2_ (0.1)	58
16	2-Cl-pyridine (2.0)	1.3	ZnCl_2_ (0.1)	60
17	2-Cl-pyridine (2.0)	1.3	ZrCl_4_ (0.1)	61
18	2-Cl-pyridine (2.0)	1.3	InCl_2_ (0.1)	66
19	2-Cl-pyridine (2.0)	1.3	FeCl_3_ (0.1)	74
20	2-Cl-pyridine (2.0)	1.3	SnCl_2_ (0.1)	78
21	2-Cl-pyridine (2.0)	1.3	MgCl_2_ (0.1)	88

a Reaction conditions: compound 1 (1.0 mmol), base, Tf_2_O, Grignard reagent (Ph-MgBr) 2 (1.5 mmol), additive (0.1 mmol), CH_2_Cl_2_ (4 mL), 30 min.

b Isolated yield after purification by flash column chromatography.

c No reaction.

Next, various amounts of 2-Cl-pyridine and trifluoromethanesulfonyl anhydride were examined. The synthetic yield was affected by the amount of 2-Cl-pyridine. Addition of increased amounts of 2-Cl-pyridine to the reaction resulted in an enhanced reaction yield of the corresponding product (Table 1, entries 9–11). However, greater than 2 equiv. of 2-Cl-pyridine did not enhance the reaction yield any further during amide formation. In addition, more than 1.3 equiv. of trifluoromethanesulfonyl anhydride did not provide increased synthetic yield (entries 13 and 14), and addition of 1.0 equiv. or less of trifluoromethanesulfonyl anhydride provided a reduced yield. Based on these reaction yields, 2 equiv. of 2-Cl-pyridine and 1.3 equiv. of trifluoromethanesulfonyl anhydride were selected for subsequent study of amide synthesis.

Furthermore, additives for the reactions were investigated. In this study, various Lewis acids were employed as additives for the reaction. BiCl_3_, ZnCl_2_, and ZrCl_4_ did not provide increased synthetic yields. When the reaction was conducted in the presence of InCl_2_, FeCl_3_, and SnCl_2_, the reaction yield for the target product increased but remained unsatisfactory. However, when MgCl_2_ was utilized in the reaction, the target amide was obtained in a significantly increased yield (88%), indicating that MgCl_2_ was the most effective additive for direct amidation from *N*-Alloc-protected amines.

Next, several solvents were investigated to further optimize the reaction conditions (Table S1†). Reactions in 1,4-dioxane, MeCN, and toluene resulted in a low yield of amide. However, when dichloromethane (CH_2_Cl_2_) was used as the reaction solvent, the synthetic yield was enhanced significantly, indicating that CH_2_Cl_2_ is the most effective solvent for the amidation.

After the optimized reaction conditions were determined, the scope of the one-pot synthesis of amides was investigated (Table 2). First, *N*-Alloc-protected aromatic compounds were explored for preparation of amides. Reactions of *N*-Alloc-protected aniline with aromatic Grignard reagents bearing electron-donating substituents (methyl- and methoxy-) and electron-withdrawing substituents (chloro- and trifluoromethyl-) provided the corresponding amides (3a–3f) in high yield. In addition, several aliphatic Grignard reagents were treated with *N*-Alloc-protected aniline to give the desired products (3g and 3h) at 92% and 90% yield, respectively. Reactions of various *N*-Alloc-protected aniline with electron-donating group (methyl, di-methyl) and electron-withdrawing group (chloro-, and cyano-) were readily converted to the corresponding amides (3j–3q). Reactions of *N*-Alloc-protected aromatic compounds bearing MOM-protected alcohol (MON ether), phenyl protected amines (benzamide), and ester also produced the desired amides (3r–3t) in high yield, suggesting that this reaction protocol is useful to successfully produce the corresponding amides.

**Table 2:** Scope of amidation from *N*-Alloc-protected aryl amines and Grignard reagent^a^

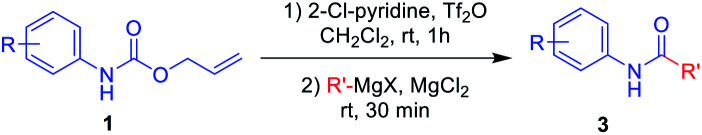
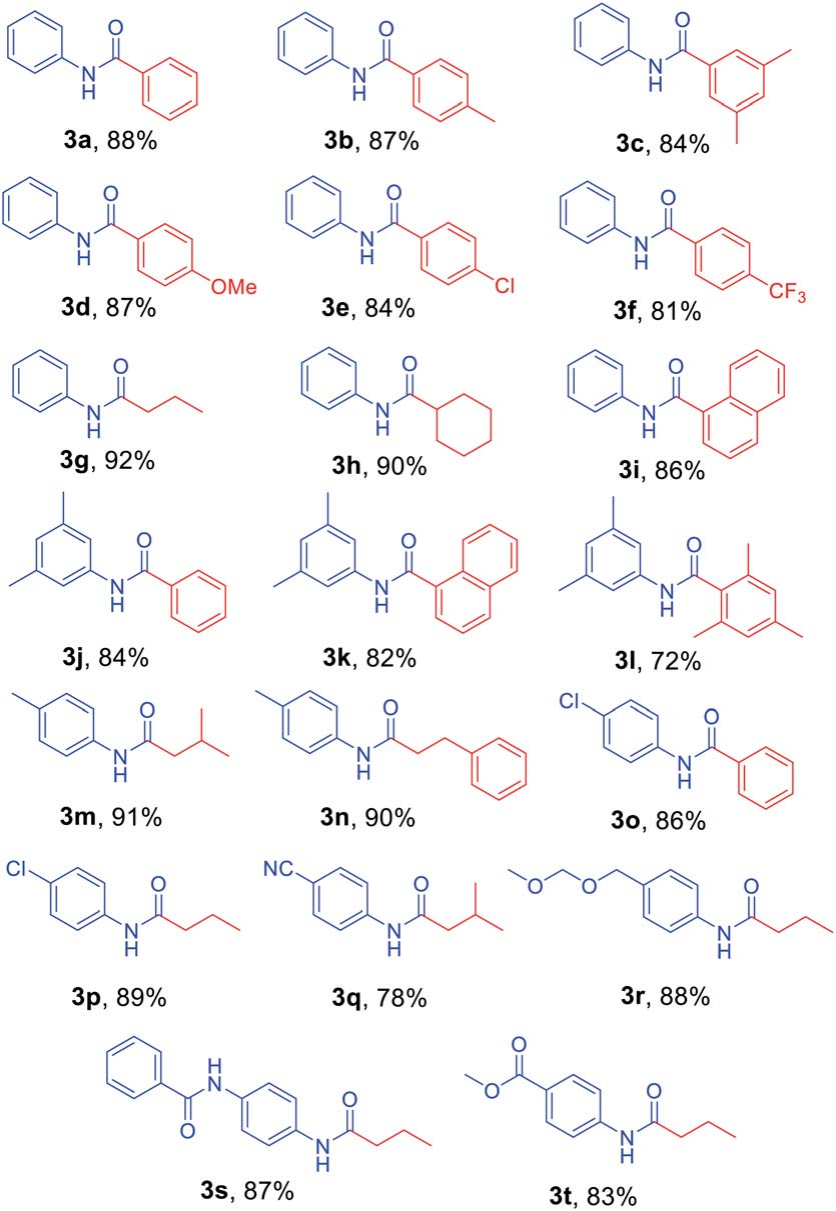

a Reaction conditions: compound 1 (1.0 mmol), 2-Cl-pyridine (2.0 mmol), Tf_2_O (1.3 mmol), Grignard reagent 2 (1.5 mmol), CH_2_Cl_2_ (4 mL), 30 min.

Next, *N*-Alloc-protected aliphatic amines were employed as substrates in this synthetic method to yield various amides (Table 3). *N*-Alloc-protected benzylic amines were treated with aryl and aliphatic Grignard reagents to yield benzamide compounds (3u–3w) in high yields. Reactions of several *N*-Alloc-protected primary aliphatic amines from *n*-butyl amine, iso-butyl amine, and cyclohexylamine under the reaction conditions readily produced the corresponding amides with yields ranging from 87 to 91% (3x–3ac). Also, *N*-Alloc-protected piperidine, a secondary aliphatic amine, was tested for amidation, and the reaction with aryl and aliphatic Grignard reagents led to efficient preparation of amides (3ad and 3ae). Reactions of *N*-Alloc-protected compounds bearing alkene and alkyne also produced the desired amides (3af and 3ag) in high yield, *N*-Boc-protected amines are commonly used in many multi-step syntheses. Thus, the scope of a novel synthetic method was extended to synthesis of amides from *N*-Boc-protected amines (Table 4). The reaction of *N*-Boc-protected aniline with various Grignard reagents bearing electron-donating groups and electron-withdrawing groups successfully yielded the desired amides (3a, 3e, and 3ah–3ak) with yields ranging from 80 to 92% at room temperature. Various *N*-Boc-protected aniline-containing electron-donating groups and electron-withdrawing groups were successfully treated with Grignard reagent to provide the target amides (3j, 3o, and 3al–3ao) in high yield. Furthermore, various *N*-Boc-protected aliphatic amines (benzylic amines, iso-butyl amine, and cyclohexylamine) were utilized for synthesis of amides (3u, 3z, 3ab, and 3ap–3ar), and the desired amides were obtained in high yields (85–90%). These results clearly demonstrated that treatment of *N*-Boc-protected amines with 2-chloropyridine and trifluoromethanesulfonyl anhydride, followed by Grignard reagent led to successful production of amides at high yields.

**Table 3:** Scope of amidation from *N*-Alloc-protected alkyl amines and Grignard reagent^a^

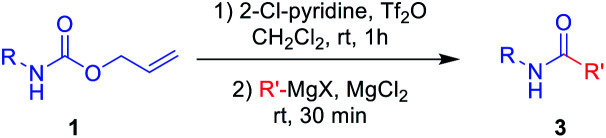
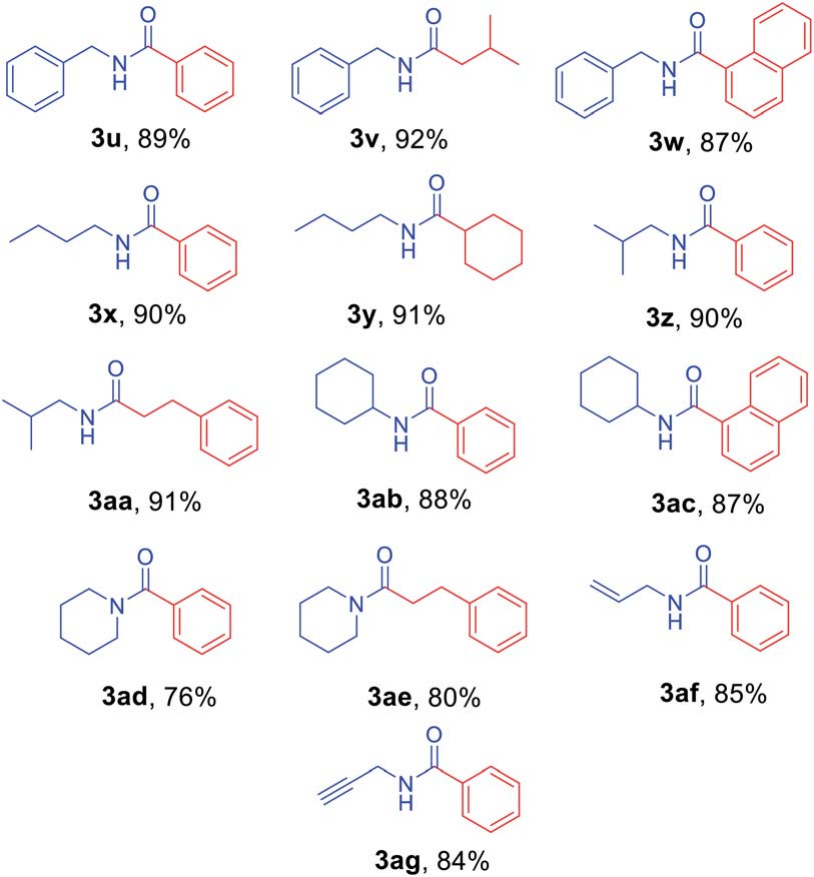

a Reaction conditions: compound 1 (1.0 mmol), 2-Cl-pyridine (2.0 mmol), Tf_2_O (1.3 mmol), Grignard reagent 2 (1.5 mmol), CH_2_Cl_2_ (4 mL), 30 min.

**Table 4:** Scope of amidation from *N*-Boc-protected amines and Grignard reagent^a^

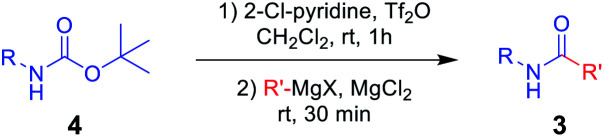
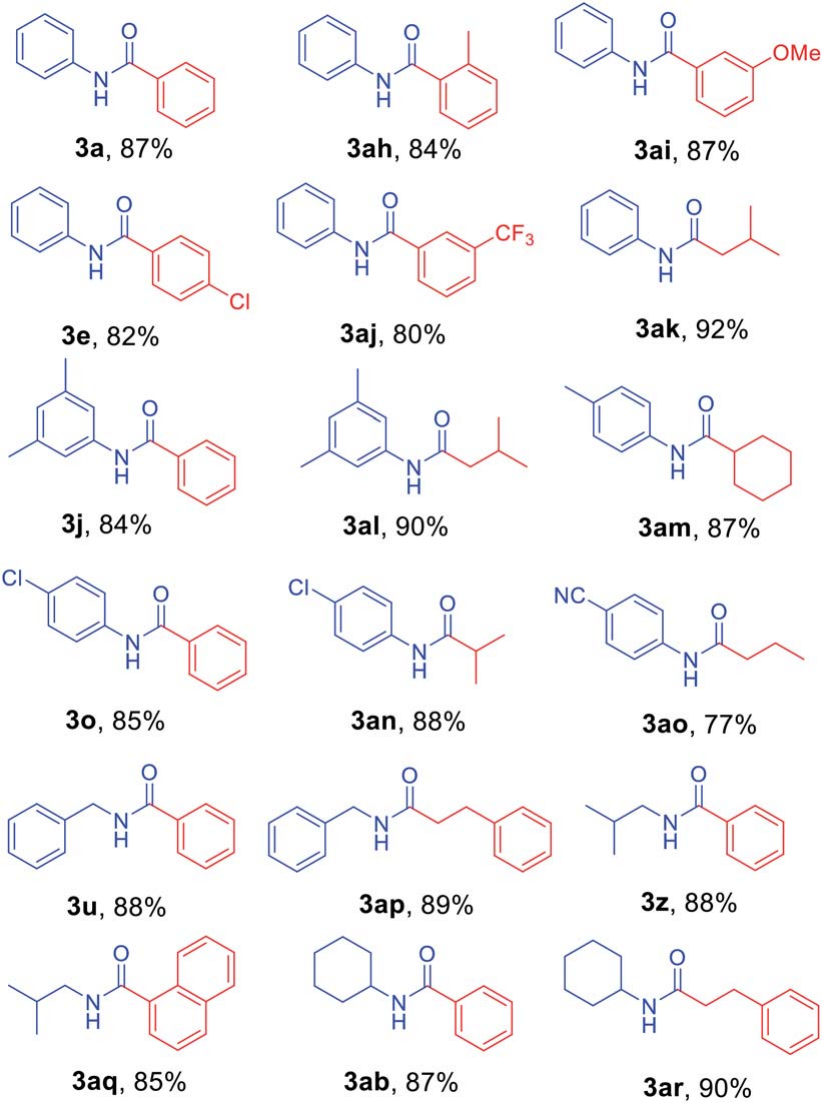

a Reaction conditions: compound 4 (1.0 mmol), 2-Cl-pyridine (2.0 mmol), Tf_2_O (1.3 mmol), Grignard reagent 2 (1.5 mmol), CH_2_Cl_2_ (4 mL), 30 min.

To further investigate the substrate scope for this amide synthesis, *N*-Cbz-protected amines were tested. As shown in Table 5, *N*-Cbz-protected amines were easily transformed to target amides in high yields under the same reaction conditions. In particular, the reactions of *N*-Cbz-protected aryl amines from aniline, dimethyl aniline, chloro aniline, and cyano aniline with different Grignard reagents generated the corresponding amides (3a, 3c, 3j, 3o, and 3as–3ax) at high yield. Reactions of *N*-Cbz-protected aliphatic amines (benzylic amines and cyclohexyl amines) were conducted, and the desired amides (3u, 3ab, and 3ay–3ba) were successfully synthesized *via* the reaction procedure using 2-chloropyridine, trifluoromethanesulfonyl anhydride, and Grignard reagent, in that order. These findings demonstrate successful one-pot transformation of *N*-Cbz-protected amines to amides using a novel amidation procedure.

**Table 5:** Scope of amidation from *N*-Cbz-protected amines and Grignard reagent^a^

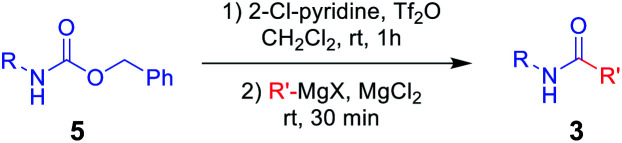
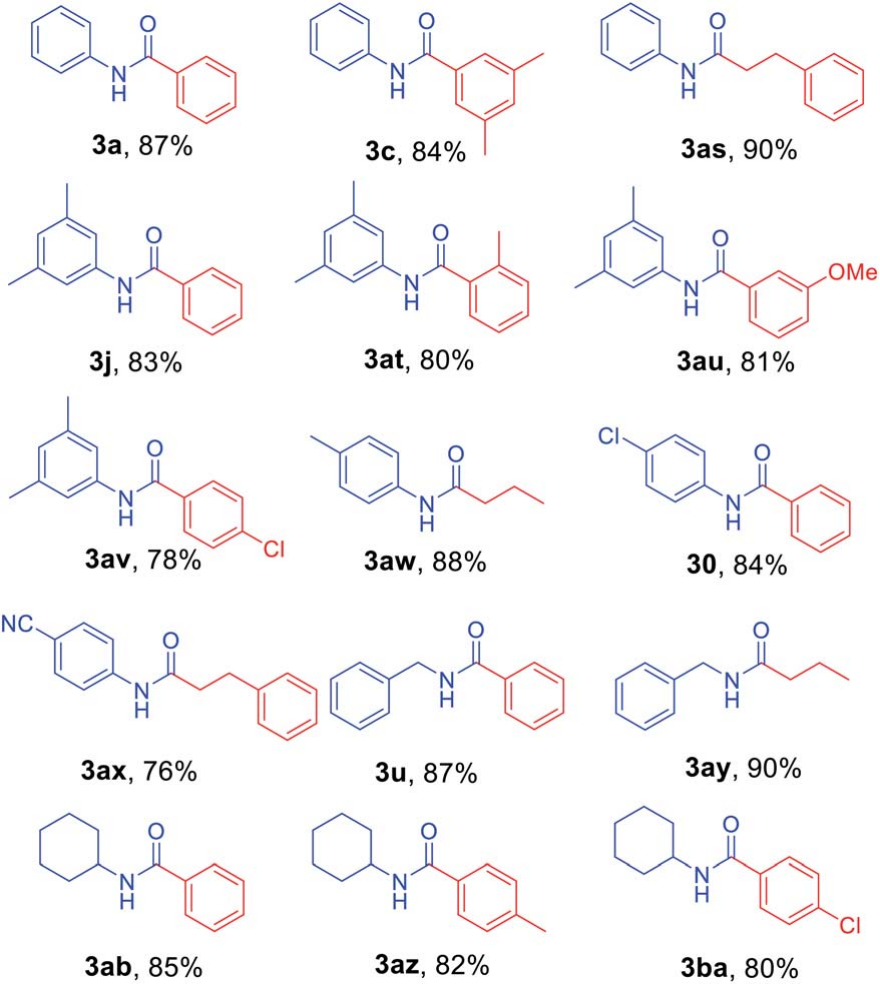

a Reaction conditions: compound 5 (1.0 mmol), 2-Cl-pyridine (2.0 mmol), Tf_2_O (1.3 mmol), Grignard reagent 2 (1.5 mmol), CH_2_Cl_2_ (4 mL), 30 min.

**Scheme 2:**
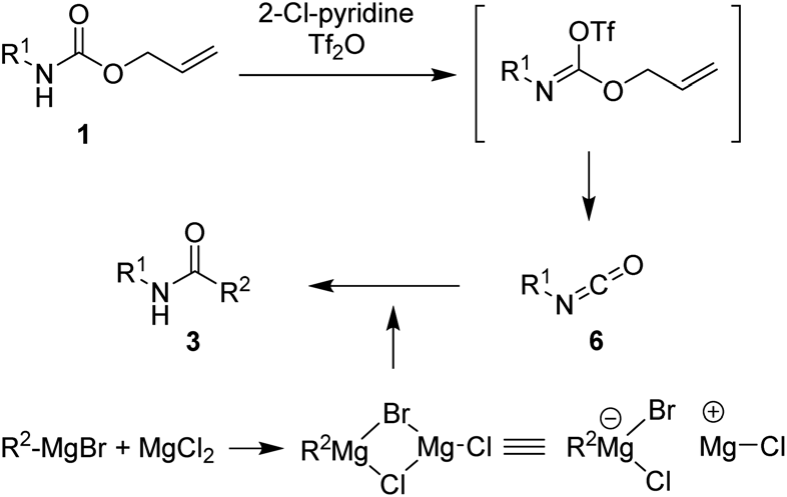
Proposed reaction mechanism.

Several previous studies showed that addition of addictive to phenylmagnesium chloride derivatives provided complex to increase reactivity of Grignard reagent.^35–38^ Thus, it can be assumed that employment of addictive such as MgCl_2_, SnCl_2_, and FeCl_3_ in this study enhanced the reaction yields *via* similar concept. A plausible mechanism for the synthesis of amides from protected amines is as shown in Scheme 2. The initial addition of 2-chloropyridine and trifluoromethanesulfonyl anhydride to *N*-Alloc-protected amine 1 provided an intermediate imino triflate, which yielded the corresponding isocyanate 6. Subsequent addition of Grignard reagents and addictive gave the target product, 3.

## Experimental

### General procedure for the preparation of amide compounds

To a solution of Alloc-protected amine 1a (0.177 g, 1.00 mmol) in dichloromethane (4 mL) 2-Cl-pyridine (0.226 g, 2.0 mmol) and Tf_2_O (0.367 g, 1.3 mmol) were added dropwise over 5 min. After stirring for 1 hour at room temperature, Grignard reagent 2a (0.271 g, 1.5 mmol) and MgCl_2_ (0.009 g, 0.1 mmol) were added to the resulting mixture. The mixture was stirred at room temperature for 30 min. The reaction mixture was extracted with dichloromethane (2 × 10 mL), and then washed with water (10 mL), followed by brine (10 mL). The organic layer was dried over anhydrous sodium sulfate and concentrated under reduced pressure. The resulting residue was then purified by flash column chromatography on silica gel with EtOAc-hexane as eluent to afford the desired product 3a as a white solid (0.173 g, 88%).

## Conclusions

In conclusion, a novel efficient one-pot synthesis of amides from *N*-Alloc-, *N*-Boc-, and *N*-Cbz-protected amines was developed. In this study, *in situ*-generated isocyanates from the reaction with 2-chloropyridine and trifluoromethanesulfonyl anhydride were employed to react with Grignard reagents, providing the resulting amides with high yields. This synthetic procedure was conducted under mild conditions, and formation of amides were achieved in a short time. Our results suggest that this novel, direct, *in situ*-generated isocyanate-mediated transformation of *N*-Alloc-, *N*-Boc-and *N*-Cbz-protected amines into amides is facile and readily applicable to synthesis of various amides.

## Conflicts of interest

There are no conflicts to declare.

## Supplementary Material

RA-011-D1RA02242C-s001
