# Supplementary material for: Practical one-pot amidation of N-Alloc-, N-Boc-, and N-Cbz protected amines under mild conditions
Source: RSC Adv. 2021 Apr 28;11(26):15890–5. doi: 10.1039/d1ra02242c (PMC9030462; doi:10.1039/d1ra02242c)
Supplement: RA-011-D1RA02242C-s001 [file RA-011-D1RA02242C-s001.pdf]

Electronic Supplementary Material (ESI) for RSC Advances.
This journal is © The Royal Society of Chemistry 2021

Supplementary Material

Practical One-Pot Amidation of *N*-Alloc-, *N*-Boc-, and *N*-Cbz Protected Amines under Mild Conditions

Wan Pyo Hong,[a] Van Hieu Tran,[b,c] and Hee-Kwon Kim *[b,c]

[a] Department of Advanced Materials and Chemical Engineering, Daegu Catholic University, 13-13, Hayang-ro, Hayang-eup, Gyeongsan-si, Gyeongbuk, 38430, Republic of Korea

[b] Department of Nuclear Medicine, Molecular Imaging & Therapeutic Medicine Research Center, Jeonbuk National University Medical School and Hospital, Jeonju, 54907, Republic of Korea

[c] Research Institute of Clinical Medicine of Jeonbuk National University-Biomedical Research Institute of Jeonbuk National University Hospital, Jeonju, 54907, Republic of Korea

* Corresponding author.

E-mail address: hkkim717@jbnu.ac.kr (H. Kim).

Table of Content

1. General Information S3

2. Screening of solvents for the preparation of amides S4

3. General procedure of the synthesis of amides S5

4. Characterization of Products S6

5. Reference S28

6. NMR Spectra S33

1. General Information

All chemicals were purchased from Sigma-Aldrich and used without further purification. Reaction progress was monitored by thin-layer chromatography (TLC) analysis. TLC analysis was performed using an aluminum plate with silica gel 60 F254, and TLC spots were visualized by UV light (254nm) exposure. Flash chromatography was performed using 230–400 mesh silica gel and analytical grade solvent. Melting points were recorded using a Stuart SMP10 Melting Point Apparatus. 1H and 13C NMR spectra were recorded on a 600 MHz & 150 MHz respectively JEOL JNM-ECA600 spectrometer or a 400 MHz & 100 MHz respectively Bruker Avance 400 spectrometer. The chemical shifts were reported in δ units (ppm) relative to the residual protonated solvent resonance, and the coupling constants (J) quoted in Hz.

2. Screening of solvents for the preparation of amides

Table S1. Screening of solvents for the preparation of amides[a]

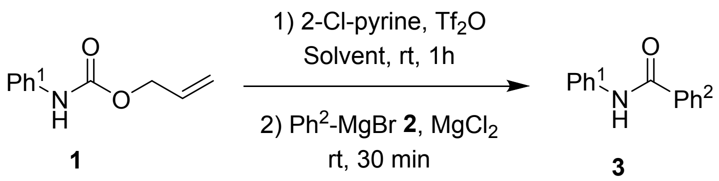

Entry	Solvent	Yield[b] (%)
1	1,4-dioxane	NR[c]
2	MeCN	7
3	THF	8
4	toluene	51
5	Ether	42
6	CH_2Cl_2	88

[a] Reaction conditions: compound **1** (1.0 mmol), 2-Cl-pyridine (2.0 mmol), Tf_2O (1.3 mmol), Grignard reagent **2** (1.5 mmol), $MgCl_2$, CH_2Cl_2 (4 mL), 30 min
[b] Isolated yield after purification of flash column chromatography.

[c] No reaction.

3. General procedure of the synthesis of amides

To a solution of Alloc-protected amine **1a** (0.177 g. 1.00 mmol) in dichloromethane (4 mL) 2-Cl-pyridine (0.226 g. 2.0 mmol) and Tf_2O (0.367 g. 1.3 mmol) were added dropwise over 5 min. After stirring for 1 hour at room temperature, Grignard reagent **2a** (0.271 g. 1.5 mmol) and $MgCl_2$ (0.009 g. 0.1 mmol) were added to the resulting mixture. The mixture was stirred at room temperature for 30 min. The reaction mixture was extracted with dichloromethane (2 x 10 mL), and then washed with water (10 mL), followed by brine (10 mL). The organic layer was dried over anhydrous sodium sulfate and concentrated under reduced pressure. The resulting residue was then purified by flash column chromatography on silica gel with EtOA-hexanes as eluent to afford the desired product **3a** as a white solid (0.173 g, 88%).

4. Characterization of Products

N-phenylbenzamide (3a) [1]

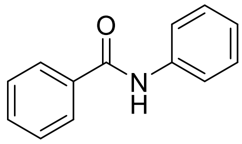

White solid; m.p. 166-168 °C;

^{1}H NMR (400 MHz, DMSO-d6) δ 10.25 (s, 1H), 7.97 – 7.95 (dt, J = 8.0 Hz, J = 2.0 Hz, 2H), 7.67 (dt, J = 8.8 Hz, J = 1.2 Hz, 2H), 7.62 – 7.52 (m, 3H), 7.38 – 7.34 (tt, J = 7.2 Hz, J = 2.0 Hz, 2H), 7.13 – 7.09 (tt, J = 7.2 Hz, J = 0.8 Hz, 1H);

^{13}C NMR (100 MHz, DMSO-d6) δ 166.02, 139.64, 135.46, 132.01, 129.07 (2C), 128.85 (2C), 128.12 (2C), 124.12, 120.82 (2C);

HRMS (ESI) m/z $(M+H)^+$ calcd for $C_{13}H_{12}NO$ = 198.0919, found 198.0920.

4-methyl-N-phenylbenzamide (3b) [2]

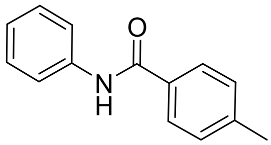

White solid; m.p. 181- 183°C;

^{1}H NMR (600 MHz, $CDCl_3$) δ 8.02 (s, 1H), 7.78 (d, J = 8.4 Hz, 2H), 7.67 (d, J = 8.4 Hz, 2H), 7.36 (t, J = 8.4 Hz, 2H), 7.27 (d, J = 7.2 Hz, 2H), 7.17 – 7.14 (td, J = 7.2 Hz, J = 1.8 Hz, 1H), 2.43 (s, 3H);

^{13}C NMR (150 MHz, $CDCl_3$) δ 165.91, 142.41, 138.18, 132.18, 129.48(2C), 129.13 (2C), 127.17 (2C), 124.5, 120.35 (2C), 21.59;

HRMS (ESI) m/z $(M+H)^+$ calcd for $C_{14}H_{14}NO$ = 212.1075, found 212.1078.

3,5-dimethyl-*N*-phenylbenzamide (3c) [3]

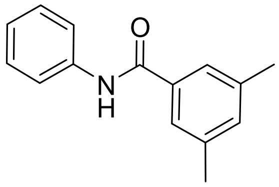

White solid; m.p. 176 - 178 °C;

^{1}H NMR (400 MHz, $CDCl_3$) δ 7.82 (s, 1H), 7.68 (d, J = 7.6 Hz, 2H), 7.49 (s, 2H), 7.39 (t, J = 7.2 Hz, 2H), 7.19 – 7.15 (m, 2H), 2.41 (s, 6H);

^{13}C NMR (100 MHz, $CDCl_3$) δ 166.08, 138.55 (2C), 138.06, 135.05, 133.46, 129.09 (2C), 1224.77 (2C), 124.44, 120.12 (2C), 21.31 (2C);

HRMS (ESI) m/z $(M+H)^+$ calcd for $C_{15}H_{16}NO$ = 226.1232, found 226.1234.

4-methoxy-*N*-phenylbenzamide (3d) [1]

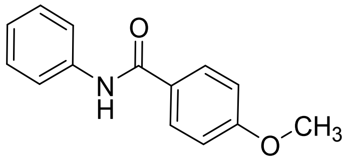

White solid; m.p. 167-168 °C;

^{1}H NMR (600 MHz, DMSO-d6) δ 10.04 (s, 1H), 7.94 – 7.92 (dt, J = 9.0 Hz, J = 1.8 Hz, 2H), 7.74 (dd, J = 7.2 Hz, J = 1.8 Hz, 2H), 7.29 (t, J = 7.8 Hz, 2H), 7.05 – 7.01 (m, 3H) 3.79 (s, 3H); ^{13}C NMR (150 MHz, DMSO-d6) δ 165.42, 162.42, 139.89, 130.11(2C), 129.07 (2C), 127.53, 123.93, 120.87 (2C), 114.11 (2C), 55.95;

HRMS (ESI) m/z $(M+H)^+$ calcd for $C_{14}H_{14}NO_2$ = 228.1025, found 228.1024.

4-chloro-*N*-phenylbenzamide (3e) [4]

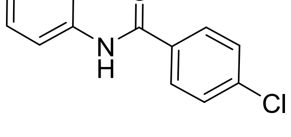

White solid; m.p. 197 -198 °C;

^{1}H NMR (600 MHz, DMSO-d6) δ 10.26 (s, 1H), 7.95 (d, J = 8.4 Hz, 2H), 7.73 (d, J = 7.8 Hz, 2H), 7.57 (d, J = 7.8 Hz, 2H), 7.31 (t, J = 7.8 Hz, 2H), 7.06 (t, J = 7.2 Hz, 1H);

^{13}C NMR (150 MHz, DMSO-d6) δ 164.95, 139.49, 136.90, 134.18, 130.14 (2C), 129.15 (2C), 128.97 (2C), 124.34, 120.95 (2C);

HRMS (ESI) m/z $(M+H)^+$ calcd for $C_{13}H_{11}ClNO$ = 232.0529, found 232.0525.

***N*-phenyl-4-(trifluoromethyl)benzamide (3f) [1]**

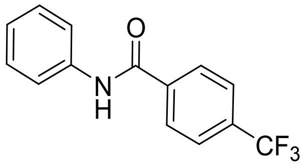

White solid; m.p. 198-200 °C;

^{1}H NMR (600 MHz, DMSO-d6) δ 10.43 (s, 1H), 8.12 (d, J = 7.8 Hz, 2H), 7.88 (d, J = 8.4 Hz, 2H), 7.76 (d, J = 7.8 Hz, 2H), 7.34 (t, J = 7.8 Hz, 2H), 7.09 (t, J = 7.2 Hz, 1H);

^{13}C NMR (150 MHz, DMSO-d6) δ 164.92, 139.62 (2C), 131.98, 131.78, 129.20 (2C), 129.13 (2C), 125.92, 125.89, 124.55, 120.99 (2C);

HRMS (ESI) m/z $(M+H)^+$ calcd for $C_{14}H_{11}F_3NO$ = 266.0793, found 266.0796.

***N*-phenylbutyramide (3g) [1]**

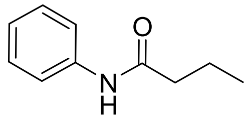

White solid; m.p. 96-98 °C;

^{1}H NMR (600 MHz, $CDCl_3$) δ 7.55 (d, J = 7.2 Hz, 2H), 7.32 (t, J = 8.4 Hz, 2H), 7.11 (t, J = 7.2 Hz, 1H), 2.35 (t, J = 7.8 Hz, 2H), 1.80 – 1.74 (m, 2H), 1.01 (t, J = 7.8 Hz, 3H);

^{13}C NMR (150 MHz, $CDCl_3$) δ 171.62, 138.09, 129.05 (2C), 124.25, 119.96 (2C), 39.73, 19.20, 13.86;

HRMS (ESI) m/z $(M+H)^+$ calcd for $C_{10}H_{14}NO$ = 164.1075, found 164.1078

***N*-phenylcyclohexanecarboxamide (3h)** [5]

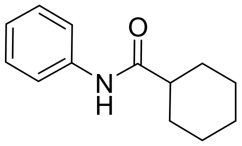

White solid; m.p. 138-140 °C;

^1H NMR (600 MHz, $CDCl_3$) δ 7.56 (d, J = 7.8 Hz, 2H), 7.49 (s, 1H), 7.31 (t, J = 8.4 Hz, 2H), 7.09 (t, J = 7.8 Hz, 1H), 2.28 – 2.23 (m, 1H), 1.97 (d, J = 11.4 Hz, 2H), 1.85 – 1.78 (m, 2H), 1.75 -1.69 (m, 1H), 1.59 – 1.52 (m, 2H), 1.34 – 1.22 (m, 3H);

^{13}C NMR (150 MHz, $CDCl_3$) δ 174.66, 138.26, 129.01 (2C), 124.12, 119.89 (2C), 46.58, 29.74 (2C), 25.75 (3C);

HRMS (ESI) m/z $(M+H)^+$ calcd for $C_{13}H_{18}NO$ = 204.1388, found 204.1389.

***N*-phenyl-1-naphthamide (3i)** [2]

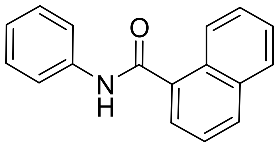

White solid; m.p. 160-162 °C;

^1H NMR (600 MHz, DMSO-d6) δ 10.54 (s, 1H), 8.17 (dd, J = 6.0 Hz, J = 3.0 Hz, 1H), 8.05 (d, J = 8.4 Hz, 1H), 7.99 – 7.97 (m, 1H), 7.80 (d, J = 7.8 Hz, 2H), 7.73 (d, J = 7.2 Hz, 1H), 7.59 – 7.54 (m, 3H), 7.35 (t, J = 7.8 Hz, 2H), 7.09 (t, J = 7.2 Hz, 1H);

^{13}C NMR (150 MHz, DMSO-d6) δ 167.82, 139.88, 135.36, 133.70, 130.61, 130.21, 129.26 (2C), 128.86, 127.52, 126.89, 125.96, 125.66, 125.58, 124.22, 120.36 (2C);

HRMS (ESI) m/z $(M+H)^+$ calcd for $C_{17}H_{14}NO$ = 248.1075, found 248.1078.

***N*-(3,5-dimethylphenyl)benzamide (3j) [2]**

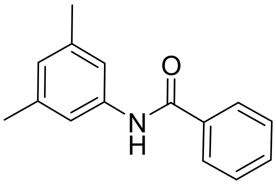

White solid; m.p. 144 – 146 °C; (lit. m.p. 142-143 °C)

^{1}H NMR (600 MHz, $CDCl_3$) *δ* 7.94 (s, 1H), 7.88 (d, *J* = 9.0 Hz, 2H), 7.54 (t, *J* = 7.2 Hz, 1H), 7.47(t, *J* = 7.2 Hz, 2H), 7.31 (s, 2H), 6.81 (s, 1H), 2.32 (s, 6H);

^{13}C NMR (150 MHz, $CDCl_3$) *δ* 165.84, 138.84 (2C), 137.87, 135.19, 131.81, 128.81 (2C), 127.12 (2C), 126.39, 118.13 (2C), 21.48 (2C);

HRMS (ESI) m/z $(M+H)^+$ calcd for $C_{15}H_{16}NO$ = 226.1232, found 226.1233.

***N*-(3,5-dimethylphenyl)-1-naphthamide (3k) [6]**

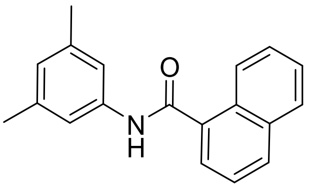

White solid; m.p. 180 - 182 °C;

^{1}H NMR (600 MHz, $CDCl_3$) *δ* 8.36 (dd, *J* = 6.0 Hz, *J* = 3.6 Hz, 1H), 7.95 (d, *J* = 7.8 Hz, 1H), 7.91 – 7.89 (m, 1H), 7.40 (s, 1H), 7.69 (d, *J* = 7.2 Hz, 1H), 7.57 – 7.54 (m, 2H), 7.47 (t, *J* = 7.2 Hz, 1H), 7.34 (s, 2H), 6.85 (s, 1H), 2.36 (s, 6H);

^{13}C NMR (150 MHz, DMSO-d6) *δ* 167.61, 138.98 (2C), 137.99, 134.73, 133.82, 131.01, 130.15, 128.49, 127.38, 126.64, 126.65, 125.39, 125.10, 124.82, 117.79 (2C), 21.53 (2C);

HRMS (ESI) m/z $(M+H)^+$ calcd for $C_{19}H_{18}NO$ = 276.1388, found 276.1387.

N-(3,5-dimethylphenyl)-2,4,6-trimethylbenzamide (3l)

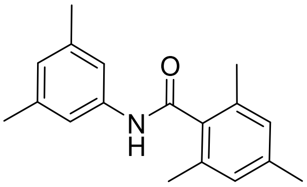

White solid; m.p. 147-148 °C;

^{1}H NMR (400 MHz, $CDCl_3$) δ 7.27 (s, 2H), 7.23 (s, 1H), 6.91 (s, 2H), 6.83 (s, 1H), 2.37 (s, 6H), 2.35 (s, 6H), 2.32 (s, 3H);

^{13}C NMR (100 MHz, $CDCl_3$) δ 168.69, 138.96 (2C), 138.86 (2C), 137.70, 135.15, 134.32, 128.35 (2C), 126.36, 117.50 (2C), 21.41 (2C), 21.15, 19.20 (2C);

HRMS (ESI) m/z $(M+H)^+$ calcd for $C_{18}H_{22}NO$ = 268,1701 found 268.1704.

3-methyl-N-(p-tolyl)butanamide (3m) [7]

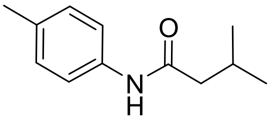

White solid; m.p. 95-97 °C;

^{1}H NMR (600 MHz, $CDCl_3$) δ 7.43 (d, J = 7.8 Hz, 3H), 7.13 (d, J = 7.8 Hz, 2H), 2.32 (s, 3H), 2.24 – 2.21 (m, 3H), 1.02 (d, J = 6.6 Hz, 6H);

^{13}C NMR (150 MHz, $CDCl_3$) δ 170.98, 135.48, 133.88, 129.51 (2C), 120.13 (2C), 47.09, 26.38, 22.55 (2C), 20.94;

HRMS (ESI) m/z $(M+H)^+$ calcd for $C_{12}H_{18}NO$ = 192.1388, found 192.1384.

3-phenyl-N-(p-tolyl)propanamide (3n) [8]

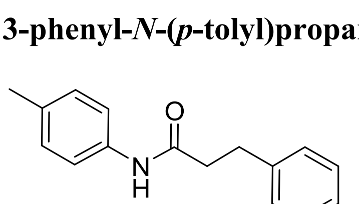

White solid; m.p. 131-132 °C;

^{1}H NMR (600 MHz, $CDCl_3$) δ 7.35 – 7.29 (m, 5H), 7.25 – 7.23 (m, 3H), 7.11 (d, J = 8.4 Hz,

2H), 3.05 (t, J = 7.2 Hz, 2H), 2.65 (t, J = 7.8 Hz, 2H), 2.32 (s, 3H);

^{13}C NMR (150 MHz, $CDCl_3$) δ 170.53, 140.81, 135.29, 134.02, 129.52 (2C), 128.72 (2C), 128.49 (2C), 126.44, 120.23 (2C), 39.45, 31.72, 20.96;

HRMS (ESI) m/z $(M+H)^+$ calcd for $C_{16}H_{18}NO$ = 240.1388, found 240.1387.

***N*-(4-chlorophenyl)benzamide (3o) [4]**

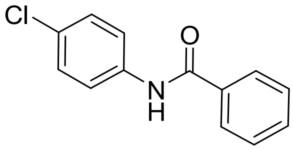

White solid; m.p. 192-194 °C;

^{1}H NMR (600 MHz, $CDCl_3$) δ 7.89 (d, J = 9.0 Hz, 2H), 7.83 (s, 1H), 7.63 – 7.61 (dt, J = 9.0 Hz, J = 2.4 Hz, 2H), 7.59 – 7.57 (dt, J = 7.2 Hz, J = 1.8 Hz, 1H), 7.52 (t, J = 8.4 Hz, 2H), 7.37 – 7.35 (dt, J = 8.4 Hz, J = 1.2 Hz, 2H);

^{13}C NMR (150 MHz, $CDCl_3$) δ 165.76, 136.56, 134.69, 132.18, 129.64, 129.22 (2C), 128.97 (2C), 127.09 (2C), 121.48 (2C);

HRMS (ESI) m/z $(M+H)^+$ calcd for $C_{13}H_{11}ClNO$ = 232.0529, found 232.0527.

***N*-(4-chlorophenyl)butyramide (3p)**

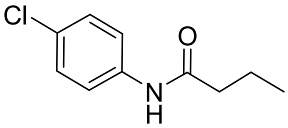

White solid; m.p. 105-106 °C;

^{1}H NMR (600 MHz, $CDCl_3$) δ 7.72 – 7.70 (dt, J = 9.0 Hz, J = 2.4 Hz, 2H), 7.39 – 7.37 (dt, J = 9.0 Hz, J = 2.4 Hz, 2H), 7.28 (s, 1H), 3.42 (q, J = 6.6 Hz, 2H), 1.67 – 1.60 (m, 2H), 0,98 (t, J = 6.6 Hz, 3H);

^{13}C NMR (150 MHz, $CDCl_3$) δ 166.61, 137.56, 133.26, 128.81 (2C), 128.39(2C), 41.92, 22.94, 11.51; HRMS (ESI) m/z $(M+H)^+$ calcd for $C_{10}H_{13}ClNO$ = 198.0686, found 198.0688.

***N*-(4-cyanophenyl)-3-methylbutanamide (3q)**

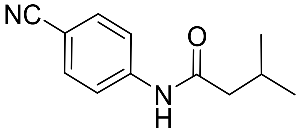

White solid; m.p. 114 -116 °C;

^{1}H NMR (600 MHz, $CDCl_3$) *δ* 7.85 (s, 1H), 7.72 (d, *J* = 9.0 Hz, 2H), 7.61 – 7.59 (dt, *J* = 9.0 Hz, *J* = 1.8 Hz, 2H), 2.28 (d, *J* = 6.6 Hz, 2H), 2.24 – 2.19 (m, 1H), 1.02 (d, *J* = 6.6 Hz, 6H);

^{13}C NMR (150 MHz, $CDCl_3$) *δ* 171.64, 142.31, 133.33 (2C), 119.63 (2C), 119.06, 106.77, 47.05, 26.29, 22.52 (2C);

HRMS (ESI) m/z $(M+H)^+$ calcd for $C_{12}H_{15}N_2O$ = 203.1184, found 203.1187.

***N*-(4-((methoxymethoxy)methyl)phenyl)butyramide (3r)**

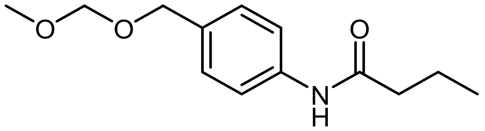

Colorless oil; ^{1}H NMR (400 MHz, $CDCl_3$) δ 7.53 (d, *J* = 8.0 Hz, 2H), 7.34 (d, *J* = 8.4 Hz, 2H), 7.20 (s, 1H), 4.71 (s, 2H), 4.57 (s, 2H), 3.42 (s, 3H), 2.35 (t, *J* = 7.6 Hz, 2H), 1.81 – 1.74 (m, 2H), 1.03 (t, *J* = 7.6 Hz, 3H); ^{13}C NMR (100 MHz, $CDCl_3$) *δ* 171.17, 137.48, 133.66, 128.73 (2C), 119.70 (2C), 95.57, 68.73, 55.36, 39.71, 19.05, 13.76; HRMS (ESI) m/z $(M+H)^+$ calcd for $C_{13}H_{20}NO_3$ = 238.1443, found 238.1445.

***N*-(4-butyramidophenyl)benzamide (3s)**

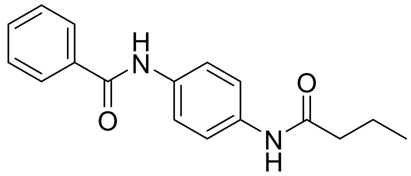

White solid; m.p 235 -237 ^{o}C; ^{1}H NMR (400 MHz, DMSO-d6) δ 10.19, 9.84, 7.96 (d, *J* = 7.2 Hz, 2H), 7.69 (d, *J* = 8.8 Hz, 2H), 7.61 – 7.51 (m, 5H), 2.28 (t, *J* = 7.2 Hz, 2H), 1.65 – 1.59 (m, 2H), 0.93 (t, *J* = 7.2 Hz, 3H); ^{13}C NMR (100 MHz, DMSO-d6) *δ* 171.31, 165.67, 135.71,

135.42, 134.76, 131.89, 128.81 (2C), 128.04 (2C), 121.26 (2C), 119.71 (2C), 38.75, 19.08, 14.15; HRMS (ESI) m/z $(M+H)^+$ calcd for $C_{17}H_{19}N_2O_2$ = 283.1147, found 283.1148.

Methyl 4-benzamidobenzoate (3t) [9]

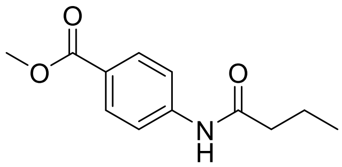

White solid; m.p. 170 -172 °C;

^{1}H NMR (400 MHz, $CDCl_3$) δ 8.08 (d, J = 8.4 Hz, 2H), 8.05 (s, 1H), 7.91 (d, J = 7.2 Hz, 2H), 7.78 (d, J = 8.8 Hz, 2H), 7.61 – 7.57 (m, 1H), 7.52 (t, J = 8.0 Hz, 2H), 3.93 (s, 3H);

^{13}C NMR (100 MHz, $CDCl_3$) δ 166.58, 165.78, 142.14, 134.55, 132.24, 130.93 (2C),128.92 (2C), 127.08 (2C), 125.89, 119.19 (2C), 52.07;

HRMS (ESI) m/z $(M+H)^+$ calcd for $C_{15}H_{14}NO_3$ = 256.0974, found 256.0977.

***N*-benzylbenzamide (3u) [10]**

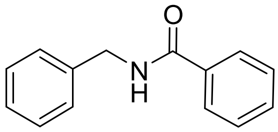

White solid; m.p. 107-109 °C;

^{1}H NMR (600 MHz, $CDCl_3$) δ 7.82 -7.81 (m, 2H), 7.53 (m, 1H), 7.44 (t, J = 7.8 Hz, 2H), 7.38 – 7.35 (m, 4H), 7.34 – 7.30 (m, 1H), 4.66 (d, J = 5.4 Hz, 2H);

^{13}C NMR (150 MHz, $CDCl_3$) δ 167.45, 138.28, 134.45, 131.63, 128.87 (2C), 128.68 (2C), 127.99 (2C), 127.70, 127.06 (2C), 44.19;

HRMS (ESI) m/z $(M+H)^+$ calcd for $C_{14}H_{14}NO$ = 212.1075, found 212.1078

***N*-benzyl-3-methylbutanamide (3v) [11]**

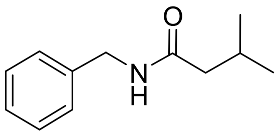

White solid; m.p. 58- 60 °C;

^{1}H NMR (600 MHz, $CDCl_3$) δ 7.33 – 7.30 (m, 2H), 7.26 – 7.24 (m, 3H), 5.83 (s, 1H), 4.42 (d, J = 4.2 Hz, 2H), 2.16 – 2.09 (m, 1H), 2.06 (d, J = 6.6 Hz, 2H), 0.95 (d, J = 6.6 Hz, 6H); ^{13}C NMR (150 MHz, $CDCl_3$) δ 172.44, 138.53, 128.78 (2C), 127.92 (2C), 127.57, 46.19, 43.62, 26.27, 22.59 (2C); HRMS (ESI) m/z $(M+H)^+$ calcd for $C_{12}H_{18}NO$ 192.1388, found 192.1385.

***N*-benzyl-1-naphthamide (3w) [10]**

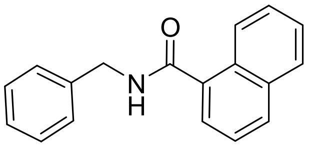

White solid; m.p. 148-150 °C;

^{1}H NMR (600 MHz, $CDCl_3$) δ 8.34 (m, 1H), 7.90 (d, J = 8.4 Hz, 1H), 7.88 – 7.85 (m, 1H), 7.58 -7.52 (m, 3H), 7.41 – 7.36 (m, 5H), 7.33 – 7.30 (m, 1H), 6.53 (s, 1H), 4.68 (d, J = 6.0 Hz, 2H);

^{13}C NMR (150 MHz, $CDCl_3$) δ 169.52, 138.25, 134.35, 133.76, 130.75, 130.26, 128.89 (2C), 128.39, 127.94 (2C), 127.68, 127.21, 126.51, 125.53, 125.05, 124.76, 44.12;

HRMS (ESI) m/z $(M+H)^+$ calcd for $C_{18}H_{16}NO$ = 262.1232, found 262.1236.

***N*-butylbenzamide (3x) [10]**

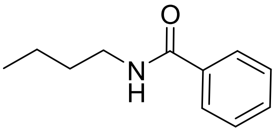

Colorless oil;

^{1}H NMR (600 MHz, $CDCl_3$) δ 7.78 (d, J = 6.0 Hz, 2H), 7.49 (t, J = 7.8 Hz, 1H), 7.42 (t, J = 7.8 Hz, 2H), 6.33 (s, 1H), 3.46 (q, J = 6.6 Hz, 2H), 1.62 – 1.58 (m, 2H), 1.45 – 1.38 (m, 2H),

0.96 (t, J = 7.2 Hz, 3H);

^{13}C NMR (150 MHz, $CDCl_3$) δ 167.69, 134.91, 131.36, 128.59 (2C), 126.94 (2C), 39.91, 31.81, 20.24, 13.88;

HRMS (ESI) m/z $(M+H)^+$ calcd for $C_{11}H_{16}NO$ = 178.1232, found 178.1234.

***N*-butylcyclohexanecarboxamide (3y) [12]**

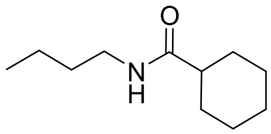

White solid; m.p. 65 - 67 °C;

^{1}H NMR (600 MHz, $CDCl_3$) δ 5.65 (s, 1H), 3.22 (q, J = 6.6 Hz, 2H), 2.07 – 2.03 (m, 1H), 1.85 – 1.82 (m, 2H), 1.78 – 1.75 (m, 2H), 1.67 – 1.64 (m, 1H), 1.48 – 1.40 (m, 4H), 1.35 – 1.29 (m, 2H), 1.28 – 1.18 (m, 3H), 0.91 (t, J = 7.8 Hz, 3H);

^{13}C NMR (150 MHz, $CDCl_3$) δ 176.18, 45.69, 39.07, 31.83, 29.81 (2C), 25.83 (3C), 20.14, 13.86;

HRMS (ESI) m/z $(M+H)^+$ calcd for $C_{11}H_{22}NO$ = 184.1701, found 184.1702.

***N*-isobutylbenzamide (3z) [13]**

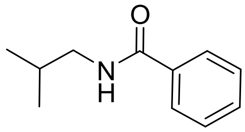

White solid; m.p. 56-58 °C;

^{1}H NMR (600 MHz, $CDCl_3$) δ 7.79 – 7.78 (m, 2H), 7.51 – 7.48 (m, 1H), 7.43 (t, J = 7.8 Hz, 2H), 6.42 (s, 1H), 3.29 (t, J = 6.0 Hz, 2H), 1.95 – 1.88 (m, 1H), 0.99 (d, J = 6.6 Hz, 6H);

^{13}C NMR (150 MHz, $CDCl_3$) δ 167.73, 135.02, 131.37, 128.60 (2C), 126.94 (2C), 47.43, 28.72, 20.27 (2C);

HRMS (ESI) m/z $(M+H)^+$ calcd for $C_{11}H_{16}NO$ = 178.1232, found 178.1235

N-isobutyl-3-phenylpropanamide (3aa) [14]

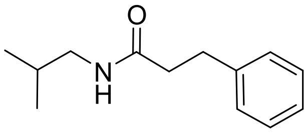

White solid; m.p. 59-61 °C;

^{1}H NMR (600 MHz, $CDCl_3$) δ 7.38 – 7.36 (m, 2H), 7.31 – 7.28 (m, 3H), 5.56 (s, 1H), 3.13 (t, J = 6.6 Hz, 2H), 3.07 (t, J = 8.4 Hz, 2H), 2.58 (t, J = 7.2 Hz, 2H), 1.79 – 1.75 (m, 1H), 0.93 (d, J = 7.2 Hz, 6H);

^{13}C NMR (150 MHz, $CDCl_3$) δ 172.15, 140.97, 128.61 (2C), 128.44 (2C), 126.31, 46.95, 38.68, 31.90, 28.49, 20.11 (2C);

HRMS (ESI) m/z $(M+H)^+$ calcd for $C_{13}H_{20}NO$ = 206.1545, found 206.1546.

N-cyclohexylbenzamide (3ab) [4]

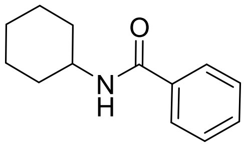

White solid; m.p. 149 -151 °C;

^{1}H NMR (600 MHz, $CDCl_3$) δ 7.77 (d, J = 6.6 Hz, 2H), 7.49 (m, 1H), 7.42 (t, J = 7.2 Hz, 2H), 6.12 (d, J = 5.4 Hz, 1H), 4.01 – 3.95 (m, 1H), 2.04 – 2.02 (m, 2H), 1.78 – 1.74 (m, 2H), 1.67 – 1.64 (m, 1H), 1.45 – 1.38 (m, 2H), 1.28 – 1.16 (m, 3H);

^{13}C NMR (150 MHz, $CDCl_3$) δ 166.72, 135.17, 131.31, 128.57 (2C), 126.93 (2C), 48.77, 33.30 (2C), 25.64, 25.03 (2C);

HRMS (ESI) m/z $(M+H)^+$ calcd for $C_{13}H_{18}NO$ = 204.1388, found 204.1386

N-cyclohexyl-1-naphthamide (3ac) [15]

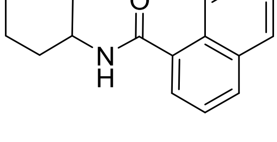

White solid; m.p. 156-157 °C;

^{1}H NMR (600 MHz, $CDCl_3$) δ 8.28 (d, J = 7.8 Hz, 1H), 7.89 -7.85 (m, 2H), 7.56 – 7.51(m, 3H), 7.43 (t, J = 7.2 Hz, 1H), 6.02 (d, J = 7.8 Hz, 1H), 4.11 – 4.04 (m, 1H), 2.11 – 2.04 (m, 2H), 1.79 – 1.75 (m, 2H), 1.69 – 1.66 (m, 1H), 1.49 – 1.42 (m, 2H), 1.28 – 1.16 (m, 3H);

^{13}C NMR (150 MHz, $CDCl_3$) δ 168.82, 135.13, 133.72, 130.34, 130.17, 128.36, 127.08, 126.43, 125.45, 124.82, 124.79, 48.82, 33.27 (2C), 25.61, 25.01 (2C);

HRMS (ESI) m/z $(M+H)^+$ calcd for $C_{17}H_{20}NO$ = 254.1545, found 254.1547.

Phenyl(piperidin-1-yl)methanone (3ad) [16]

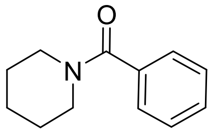

Colorless liquid;

^{1}H NMR (600 MHz, $CDCl_3$) δ 7.41 – 7.38 (m, 5H), 3.71 (s, 2H), 3.34 (s, 2H), 1.68 (s, 4H), 1.52 (s, 2H);

^{13}C NMR (150 MHz, $CDCl_3$) δ 170.39, 136.57, 129.43, 128.48 (2C), 126.85 (2C), 48.86, 43.17, 26.62, 25.74, 24.67;

HRMS (ESI) m/z $(M+H)^+$ calcd for $C_{12}H_{16}NO$ = 190.1232, found 190.1236.

3-phenyl-1-(piperidin-1-yl)propan-1-one (3ae) [8]

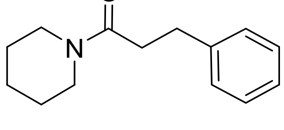

Colorless oil;

^{1}H NMR (600 MHz, $CDCl_3$) δ 7.38 (t, J = 7.2 Hz, 2H), 7.32 – 7.28 (m, 3H) 3.65 (t, J = 5.4 Hz, 2H), 3.42 (t, J = 5.4 Hz, 2H), 2.96 (t, J = 7.8 Hz, 2H), 2.53 (t, J = 7.8 Hz, 2H), 1.72 – 1.69 (m, 2H), 1.66 – 1.59 (m, 2H), 1.57 – 1.53 (m, 2H);

^{13}C NMR (150 MHz, $CDCl_3$) δ 170.48, 141.57, 128.55 (2C), 128.52 (2C), 126.16, 46.68, 42.78, 35.26, 31.70, 26.46, 25.63, 24.61;

HRMS (ESI) m/z $(M+H)^+$ calcd for $C_{14}H_{20}NO$ = 218.1545, found 218.1546.

***N*-allylbenzamide (3af)** [17]

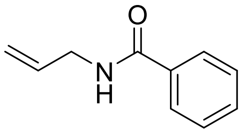

Colorless oil;

^1H NMR (400 MHz, $CDCl_3$) δ 7.81 – 7.79 (m, 2H), 7.55 – 7.50 (m, 1H), 7.48 – 7.43 (td, J = 7.2 Hz, J = 1.6 Hz, 2H), 6.23 (s, 1H), 5.99 – 5.19 (m, 1H), 5.32 – 5.26 (dq, J = 17.2 Hz, J = 1.6 Hz, 1H), 5.23 – 5.19 (dq, J = 10.4 Hz, J = 1.6 Hz, 1H), 4.12 (t, J = 5.6 Hz, 2H);

^{13}C NMR (100 MHz, $CDCl_3$) δ 167.35, 134.47, 134.15, 131.52, 128.61 (2C), 126.91 (2C), 116.75, 42.46;

HRMS (ESI) m/z $(M+H)^+$ calcd for $C_{10}H_{12}NO$ = 162.0919, found 162.0915.

***N*-(prop-2-yn-1-yl)benzamide (3ag)** [18]

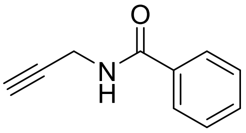

White solid; m.p. 113 - 115 °C;

^1H NMR (400 MHz, $CDCl_3$) δ 7.82 – 7.79 (m, 2H), 7.56 – 7.52 (m, 1H), 7.43 (td, J = 6.8 Hz, J = 1.6 Hz, 2H), 6.35 (s, 1H), 4.29 (dd, J = 5.2 Hz J = 2.8 Hz, 2H), 2.307 (t, J = 2.4 Hz, 1H);

^{13}C NMR (100 MHz, $CDCl_3$) δ 167.09, 133.75, 131.82, 128.66 (2C), 127.02 (2C), 79.47, 71.94, 29.82;

HRMS (ESI) m/z $(M+H)^+$ calcd for $C_{10}H_{10}NO$ = 160.0762, found 160.0767.

2-methyl-*N*-phenylbenzamide (3ah) [19]

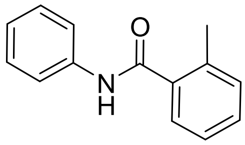

White solid; m.p. 125-127 °C;

^{1}H NMR (400 MHz, $CDCl_3$) δ 7.67 (d, J = 8.0 Hz, 2H), 7.53 (d, J = 7.2 Hz, 2H), 7.42 (t, J = 7.6 Hz, 3H), 7.34 – 7.31 (m, 2H), 7.20 (t, J = 7.6 Hz, 1H), 2.55 (s, 3H);

^{13}C NMR (100 MHz, $CDCl_3$) δ 168.05, 137.99, 136.38, 131.30, 130.32, 129.14 (2C), 126.61, 125.93, 124.75, 124.58, 119.87 (2C), 19.84;

HRMS (ESI) m/z $(M+H)^+$ calcd for $C_{14}H_{14}NO$ = 212.1075, found 212.1078.

3-methoxy-N-phenylbenzamide (3ai) [20]

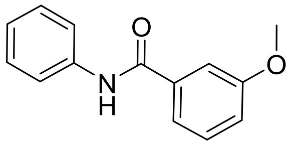

White solid; m.p. 113 - 115 °C;

^{1}H NMR (400 MHz, $CDCl_3$) δ 7.87 (s, 1H), 7.69 (d, J = 8.4 Hz, 2H), 7.47 – 7.45 (m, 1H), 7.43 – 7.37 (m, 4H), 7.21 – 7.16 (m, 1H), 7.12 – 7.08 (m, 1H), 3.88 (s, 3H);

^{13}C NMR (100 MHz, $CDCl_3$) δ 165.56, 160.01, 137.90, 136.51, 129.79, 129.13 (2C), 124.60, 120.16 (2C), 118.66, 118.05, 112.50, 55.51;

HRMS (ESI) m/z $(M+H)^+$ calcd for $C_{14}H_{14}NO_2$ = 228.1025, found 228.1028.

***N*-phenyl-3-(trifluoromethyl)benzamide (3aj) [21]**

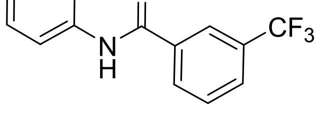

White solid; m.p. 118 - 120 °C;

^{1}H NMR (600 MHz, DMSO-d6) δ 8.12 (s, 2H), 8.05 (d, J = 7.2 Hz, 1H), 7.80 (d, J = 8.4 Hz, 1H), 7.65 (d, J = 8.4 Hz, 2H), 7.59 (t, J = 8.4 Hz, 1H), 7.37 (t, J = 7.2 Hz, 2H) 7.19 (t, J = 7.8 Hz, 1H);

^{13}C NMR (150 MHz, DMSO-d6) δ 164.59, 137.53, 135.85, 131.45, 131.23, 130.46, 129.49, 129.22 (2C), 128.47, 125.13, 124.18, 120.62 (2C);

HRMS (ESI) m/z $(M+H)^+$ calcd for $C_{14}H_{11}F_3NO$ = 266.0793, found 266.0795.

3-methyl-*N*-phenylbutanamide (3ak) [22]

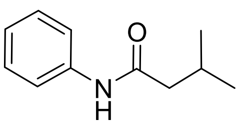

White solid; m.p. 109 - 110 °C;

^{1}H NMR (600 MHz, $CDCl_3$) δ 7.55 (d, J = 7.8 Hz, 2H), 7.52 (s, 1H), 7.32 (t, J = 8.4 Hz, 2H), 7.11 (t, J = 8.4 Hz, 1H), 2.24 – 2.22 (m, 3H), 1.03 (d, J = 7.2 Hz, 6H);

^{13}C NMR (150 MHz, $CDCl_3$) δ 171.11, 138.07, 129.03 (2C), 124.27, 120.01 (2C), 47.13, 26.37, 22.56 (2C);

HRMS (ESI) m/z $(M+H)^+$ calcd for $C_{11}H_{16}NO$ = 178.1232, found 178.1231

***N*-(3,5-dimethylphenyl)-3-methylbutanamide (3al)**

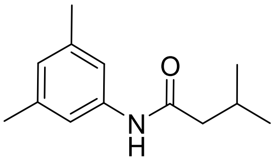

White solid; m.p. 96 - 98 °C;

^{1}H NMR (600 MHz, $CDCl_3$) δ 7.31 (s, 1H), 7.18 (s, 2H), 6.76 (s, 1H), 2.29 (s, 6H), 2.23 – 2,20 (m, 3H), 1.02 (d, J = 6.6 Hz, 6H);

^{13}C NMR (150 MHz, $CDCl_3$) δ 170.98, 138.74 (2C), 137.88, 126.01, 117.70 (2C), 47.19, 26.39, 22.55 (2C), 21.44 (2C);

HRMS (ESI) m/z $(M+H)^+$ calcd for $C_{13}H_{20}NO$ = 206.1545, found 206.1549.

***N*-(*p*-tolyl)cyclohexanecarboxamide (3am)** [16]

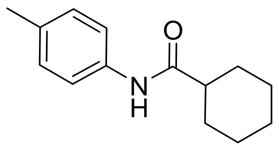

White solid; m.p. 153-155 °C;

^{1}H NMR (400 MHz, $CDCl_3$) δ 7.43 (d, J = 8.4 Hz, 2H), 7.23 (s, 1H), 7.14 (d, J = 8.4 Hz, 2H), 2.32 (s, 3H), 2.26 – 2.19 (m, 1H), 1.98 (d, J = 13.2 Hz, 2H), 1.87 - 1.83 (m, 2H), 1.73 – 1.70 (m, 1H), 1.59 – 1.51(m, 2H), 1.36 – 1.25 (m, 3H);

^{13}C NMR (100 MHz, $CDCl_3$) δ 174.32, 135.49, 133.69, 129.45 (2C), 119.83 (2C), 46.53, 29.70 (2C), 25.72 (3C), 20.89;

HRMS (ESI) m/z $(M+H)^+$ calcd for $C_{14}H_{20}NO$ = 218.1545, found 218.1546.

***N*-(4-chlorophenyl)-3-methylbutanamide (3am)** [23]

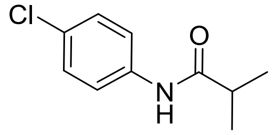

White solid; m.p. 150 - 152 °C; 1H NMR (400 MHz, CDCl3) δ 7.51 (d, J = 8.8 Hz, 2H), 7.32 – 7.27 (m, 2H), 7.25 (s, 1H), 2.56 – 2.49 (m, 1H), 1.28 (d, J = 6.8 Hz, 6H);

13C NMR (150 MHz, CDCl3) δ 175.25, 136.59, 129.11, 128.97 (2C), 121.04 (2C), 36.71, 19.59 (2C);

HRMS (ESI) m/z (M+H)+ calcd for C10H13ClNO = 198.0686, found 198.0689.

***N*-(4-cyanophenyl)butyramide (3ao)**

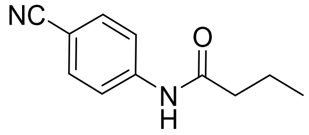

White solid; m.p. 115 - 116 °C;

^{1}H NMR (600 MHz, $CDCl_3$) δ 7.87 (s, 1H), 7.72 – 7.70 (dt, J = 9.0 Hz, J = 1.8 Hz, 2H), 7.62 – 7.59 (dt, J = 9.0 Hz, J = 1.8 Hz, 2H), 2.39 (t, J = 7.2 Hz, 2H), 1.80 – 1.74 (m, 2H), 1.01 (t, J = 7.8 Hz, 3H);

^{13}C NMR (150 MHz, $CDCl_3$) δ 172.06, 142.37, 133.34 (2C), 119.58 (2C), 119.07, 106.73, 39.69, 18.95, 13.79;

HRMS (ESI) m/z $(M+H)^+$ calcd for $C_{11}H_{13}N_2O$ = 189.1028, found 189.1032

***N*-benzyl-3-phenylpropanamide (3ap)** [24]

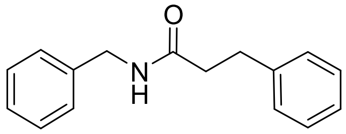

White solid; m.p. 84-86 °C;

^{1}H NMR (600 MHz, $CDCl_3$) δ 7.34 – 7.28 (m, 5H), 7.25 – 7.23 (m, 3H), 7.18 (d, J = 7.2 Hz, 2H), 5.78 (s, 1H), 4.43 (d, J = 6.0 Hz, 2H), 3,03 (t, J = 7.8 Hz, 2H), 2.55 (t, J = 6.6 Hz, 2H);

^{13}C NMR (150 MHz, $CDCl_3$) δ 172.00, 140.85, 138.22, 128.74 (2C), 128.65 (2C), 128.50 (2C), 127.82 (2C), 127.55, 126.35, 43.65, 38.58, 31.82;

HRMS (ESI) m/z $(M+H)^+$ calcd for $C_{16}H_{18}NO$ = 240.1388, found 240.1387.

***N*-isobutyl-1-naphthamide (3aq)**

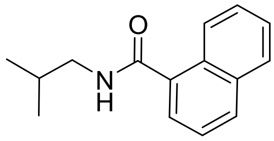

White solid; m.p. 85-87 °C;

^{1}H NMR (600 MHz, $CDCl_3$) δ 8.30 (d, J = 9.0 Hz, 1H), 7.92 (d, J = 8.4 Hz, 1H), 7.88 (d, J = 7.2 Hz, 1H), 7.59 – 7.53 (m, 3H), 7.45 (t, J = 7.2 Hz, 1H), 6.16 (s, 1H), 3.37 (t, J = 6.6 Hz, 2H), 1.98 – 1.92 (m, 1H), 1.04 (d, J = 7.2 Hz, 6H);

^{13}C NMR (150 MHz, $CDCl_3$) δ 169.73, 135.07, 133,75, 130.47, 130.21, 128.36, 127.14,

126.48, 125.52, 124.81, 124.76, 47.38, 28.77, 20.30;

HRMS (ESI) m/z $(M+H)^+$ calcd for $C_{15}H_{18}NO$ = 228.1388, found 228.1389.

***N*-cyclohexyl-3-phenylpropanamide (3ar) [25]**

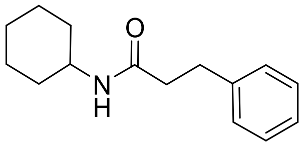

White solid; m.p. 113-115 °C;

^{1}H NMR (600 MHz, $CDCl_3$) δ 7.27 – 7.26 (m, 2H), 7.18 – 7.17 (m, 3H), 5.25 (s, 1H), 3.74 – 3.69 (m, 1H), 2.94 (t, J = 7.8 Hz, 2H), 2.42 (t, J = 7.8 Hz, 2H), 1.82 – 1.79 (m, 2H), 1.64 - 1.55 (m, 3H),1.35 – 1.27 (m, 2H), 1.12 – 1.08 (m, 1H), 1.03 -0.96 (m, 2H);

^{13}C NMR (150 MHz, $CDCl_3$) δ 171.17, 140,99, 128.57 (2C), 128.49 (2C), 126.28, 48.15, 38.88, 33.18 (2C), 31.99, 25.57, 24.89 (2C);

HRMS (ESI) m/z $(M+H)^+$ calcd for $C_{15}H_{22}NO$ = 232.1701, found 232.1705.

***N*,3-diphenylpropanamide (3as) [8]**

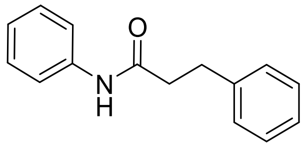

White solid; m.p. 99 - 101 °C;

^{1}H NMR (600 MHz, $CDCl_3$) 7.47 (d, J = 7.8 Hz, 2H), 7.33 – 7.29 (m, 5H), 7.25 – 7.23 (m, 3H), 7.12 (t, J = 7.8 Hz, 1H), 3.06 (t, J = 7.2 Hz, 2H), 2.67 (t, J = 7.8 Hz, 2H);

^{13}C NMR (150 MHz, $CDCl_3$) δ 170.62, 140.72, 137.83, 129.05 (2C), 128.75 (2C), 128.50 (2C), 126.49, 124.42, 120.08 (2C), 39.52, 31.66;

HRMS (ESI) m/z $(M+H)^+$ calcd for $C_{15}H_{16}NO$ = 226.1232, found 226.1234.

***N*-(3,5-dimethylphenyl)-2-methylbenzamide (3at) [26]**

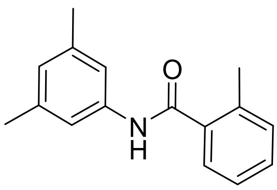

White solid; m.p. 135 – 137 °C;

^{1}H NMR (400 MHz, $CDCl_3$) δ 7.47 (d, J = 7.6 Hz, 1H), 7.37 – 7.33 (m, 2H, ArH + NH), 7.27 – 7.23 (m, 4H), 6.80 (s, 1H), 2.51 (s, 3H), 2.33 (s, 6H);

^{13}C NMR (150 MHz, $CDCl_3$) δ 168.09, 138.94 (2C), 137.93, 136.70, 136.51, 131.33, 130.29, 126.65, 126.37, 125.96, 117.66 (2C), 21.49 (2C), 19.93;

HRMS (ESI) m/z $(M+H)^+$ calcd for $C_{16}H_{18}NO$ = 240.1388, found 240.1384.

***N*-(3,5-dimethylphenyl)-3-methoxybenzamide (3au) [27]**

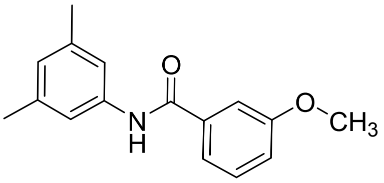

White solid; m.p. 103 - 105 °C;

^{1}H NMR (600 MHz, $CDCl_3$) δ 7.87 (s, 1H), 7.45 (d, J = 1.2 Hz, 1H), 7.39 -7.36 (m, 2H), 7.29 (s, 2H), 7.09 – 7.07 (dt, J = 6.6 Hz, J = 1.8 Hz, 1H), 6.81 (s, 1H), 3.86 (s, 3H), 2.33 (s, 6H);

^{13}C NMR (150 MHz, $CDCl_3$) δ 165.61, 160.02, 138.86 (2C), 137.81, 136.69, 129.79, 126.41, 118.73, 118,04 (3C), 112.51, 55.54, 21.48 (2C);

HRMS (ESI) m/z $(M+H)^+$ calcd for $C_{16}H_{18}NO_2$ = 256.1338, found 256.1335.

4-chloro-*N*-(3,5-dimethylphenyl)benzamide (3av) [26]

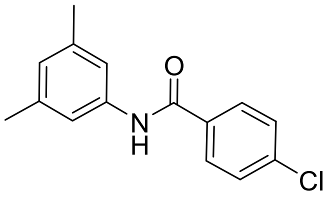

White solid; m.p. m.p. 148 - 150 °C; [lit. m.p. 141 – 144 °C]

^{1}H NMR (600 MHz, $CDCl_3$) δ 7.97 (s, 1H), 7.79 (d, J = 9.0 Hz, 2H), 7.42 (d, J = 9.0 Hz, 2H), 7.27 (s, 2H), 6.81 (s, 1H), 2.31 (s, 6H);

^{13}C NMR (150 MHz, $CDCl_3$) δ 164.85, 138.90 (2C), 138.05, 137.59, 133,49, 129.01 (2C), 128.57 (2C), 126.65, 118.28 (2C), 21.48 (2C);

HRMS (ESI) m/z $(M+H)^+$ calcd for $C_{15}H_{15}ClNO$ = 260.0842, found 260.0844.

***N*-(*p*-tolyl)butyramide (3aw)**

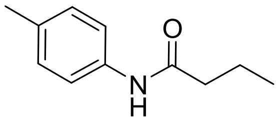

White solid; m.p. 73 - 75 °C;

^{1}H NMR (600 MHz, $CDCl_3$) δ 7.61 (s, 1H), 7.42 (d, J = 7.8 Hz, 3H), 7.12 (d, J = 7.8 Hz, 2H), 2.33 – 2.31 (m, 5H), 1.78 – 1.72 (m, 2H), 0.99 (t, J = 7.2 Hz, 3H);

^{13}C NMR (150 MHz, $CDCl_3$) δ 171.58, 135.58, 133.81, 129.48 (2C), 120.15 (2C), 39.63, 20.94, 19.23, 13.85;

HRMS (ESI) m/z $(M+H)^+$ calcd for $C_{11}H_{16}NO$ = 178.1232, found 178.1236

***N*-(4-cyanophenyl)-3-phenylpropanamide (3ax) [28]**

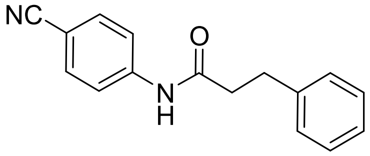

White solid; m.p. 119 - 121 °C;

^{1}H NMR (600 MHz, $CDCl_3$) δ 7.67 (s, 1H), 7.62 (d, J = 9.0 Hz, 2H), 7.57 – 7.55 (dt, J = 9.0 Hz, J = 2.4 Hz, 2H), 7.31 (t, J = 7.2 Hz, 2H), 7.25 – 7.23 (m, 3H), 3.06 (t, J = 7.2 Hz, 2H), 2.73 (t, J = 7.8 Hz, 2H);

^{13}C NMR (150 MHz, $CDCl_3$) δ 171.08, 142.09, 140.30, 133.33 (2C), 128.83 (2C), 128.43 (2C), 126.68, 119.64 (2C), 119.04, 106.87, 39.49, 31.39;

HRMS (ESI) m/z $(M+H)^+$ calcd for $C_{16}H_{15}N_2O$ = 251.1184, found 251.1187.

***N*-benzylbutyramide (3ay)** [29]

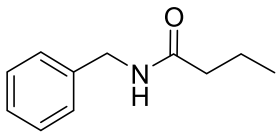

White solid; m.p. 42-44 °C;

^{1}H NMR (400 MHz, $CDCl_3$) δ 7.37 – 7.28 (m, 5H), 5.87 (s, 1H), 4.47 (d, J = 5.6 Hz, 2H), 2.22 (t, J = 7.6 Hz, 2H), 1.762 – 1.67 (m, 2H), 0.98 (t, J = 7.2 Hz, 3H);

^{13}C NMR (100 MHz, $CDCl_3$) δ 172.94, 138,36, 128.73 (2C), 127.84 (2C), 127.52, 43.62, 38.66, 19.23, 13.83;

HRMS (ESI) m/z $(M+H)^+$ calcd for $C_{11}H_{16}NO$ = 178.1232, found 178.1235.

***N*-cyclohexyl-4-methylbenzamide (3az)** [30]

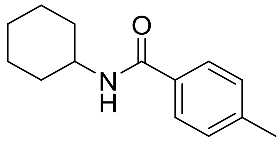

White solid; m.p. 153-155 °C;

^{1}H NMR (600 MHz, $CDCl_3$) δ 7.67 (d, J = 8.4 Hz, 2H), 7.23 (d, J = 7.2 Hz, 2H), 6.01 (d, J = 7.2 Hz, 1H), 4.01 – 3.95 (m, 1H), 2.40 (s, 3H), 2.06 – 2.02 (m, 2H), 1.81 – 1.74 (m, 2H), 1.67 - 1.65 (m, 1H), 1.46 – 1.40 (m, 2H), 1.27 – 1.19 (m, 3H);

^{13}C NMR (150 MHz, $CDCl_3$) δ 166.63, 141.67, 132.28, 129.22 (2C), 126.91 (2C), 48.65, 33.36 (2C), 25.67, 25.03 (2C), 21.52;

HRMS (ESI) m/z $(M+H)^+$ calcd for $C_{14}H_{20}NO$ = 218.1545, found 218.1547.

4-chloro-*N*-cyclohexylbenzamide (3ba) [31]

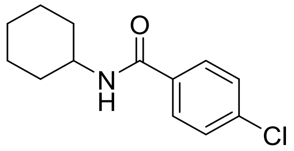

White solid; m.p. 184-185 °C;

^{1}H NMR (600 MHz, $CDCl_3$) δ 7.72 – 7.69 (dt, J = 5.6 Hz, J = 1.6 Hz, 2H), 7.41 – 7.39 (dt, J = 6.0 Hz, J = 1.2 Hz, 2H), 6.02 (d, J = 1.8 Hz, 1H), 3.98 – 3.93 (m, 1H), 2.05 – 2.02 (m, 2H), 1.78 – 1.65 (m, 2H), 1.68 – 1.65 (m, 1H), 1.47 – 1.39 (m, 2H), 1.28 – 1.17 (m, 3H);

^{13}C NMR (150 MHz, $CDCl_3$) δ 165.65, 137.50, 133.49, 128.82 (2C), 128.39 (2C), 48.92, 33.29 (2C), 25.61, 25.00 (2C);

HRMS (ESI) m/z $(M+H)^+$ calcd for $C_{13}H_{17}ClNO$ = 238.0999, found 238.0995.

5. Reference

1. Xuefeng Guo, Lin Tang, Yu Yang, Zhenggen Zha and Zhiyong Wang. An efficient synthesis of amides from alcohols and azides catalyzed by a bifunctional catalyst Au/DNA under mild conditions. Green Chem., 2014, 16, 2443.
2. Sergey A. Rzhevskiy, Alexandra A. Ageshina, Gleb A. Chesnokov, Pavel S. Gribanov, Maxim A. Topchiy, Mikhail S. Nechaev and Andrey F. Asachenko. Solvent- and transition metal-free amide synthesis from phenyl esters and aryl amines. RSC Adv., 2019, 9, 1536–1540.
3. Xian-Ying Shi, Ke-Yan Liu, Juan Fan, Xue-Fen Dong, Jun-Fa Wei and Chao-Jun Li. A Convenient Synthesis of N-Aryl Benzamides by RhodiumCatalyzed ortho-Amidation and Decarboxylation of Benzoic Acids. Chem. Eur. J. 2015, 21, 1900 – 1903.
4. Jin Zhang, Yanyan Hou, Yangmin Ma, and Michal Szostak. Synthesis of Amides by Mild Palladium-Catalyzed Aminocarbonylation of Arylsilanes with Amines Enabled by Copper (II) Fluoride. J. Org. Chem. 2019, 84, 338−345

5. Taoufik Ben Halima, Jaya Kishore Vandavasi, Mohanad Shkoor and Stephen G. Newman. A Cross-Coupling Approach to Amide Bond Formation from Esters. *ACS Catal.* 2017, 7, 3, 2176–2180

6. Diane S. W. Lim, Tedrick T. S. Lew, and Yugen Zhang. Direct Amidation of N-Boc- and N-Cbz-Protected Amines via Rhodium-Catalyzed Coupling of Arylboroxines and Carbamates. Org. Lett. 2015, 17, 6054-6057.

7. Ruiqiang Guo, Chuanlei Zhu, Zhe Sheng, Yanzhe Li, Wei Yin, Changhu Chu. Silica sulfuric acid mediated acylation of amines with 1,3-diketones via CAC bond cleavage under solvent-free conditions. Tetrahedron Letters 56 (2015) 6223–6226

8. Peng Yang, Xiuhua Wang, Yu Ma,a Yaxin Sun, Li Zhang, Jieyu Yue, Kaiyue Fu, Jianrong Steve Zhou and Bo Tang. Nickel-catalyzed C-alkylation of thioamide, amides and esters by primary alcohols through a hydrogen autotransfer strategy. *Chem. Commun.*, 2020, 56, 14083—14086.

9. Tanya L. Schneider, Kevin T. Halloran, Julie A. Hillner, Rebecca R. Conry, and Brian R. Linton. Application of H/D Exchange to Hydrogen Bonding in Small Molecules. Chem. Eur. J. 2013, 19, 15101 – 15104

10. Wei Ren and Motoki Yamane. Mo(CO)6-Mediated Carbamoylation of Aryl Halides. J. Org. Chem. 2010, 75, 8410–8415.

11. Andrea Ojeda-Porras, Alejandra Hernández-Santana and Diego Gamba-Sánchez. Direct amidation of carboxylic acids with amines under microwave irradiation using silica gel as a solid support. Green Chem., 2015, 17, 3157–3163.

12. Kien Soon Goh and Choon-Hong Tan. Metal-free pinnick-type oxidative amidation of aldehydes. : RSC Advances, 2012, 2, 5536–5538.

13. Gemma L. Thomas, Christine Bo¨hner, Mark Ladlow and David R. Spring. Synthesis and utilization of functionalized polystyrene resins. *Tetrahedron.*, 2005, **61**, 12153–12159

14. Jianfei Bai, Bartosz K. Zambron, and Pierre Vogel. Amides in One Pot from Carboxylic Acids and Amines via Sulfinylamides. Org. Lett. 2014, 16, 604−607.

15. Weiwei Fang, Qinyue Deng, Mizhi Xu, and Tao Tu. Highly Efficient Aminocarbonylation of Iodoarenes at Atmospheric Pressure Catalyzed by a Robust Acenaphthoimidazolyidene Allylic Palladium Complex. *Organic Letters*. 2013, Vol. **15**, No. 14, 3678–3681.

16. Subeen Yu, Kwang Ho Song, and Sunwoo Lee. Metal-Free Transamidation of Primary Amides using Trimethylsilyl Chloride. Asian J. Org. Chem. 2019, 8, 1613 – 1616.

17. Yongmei Liu, Shicheng Shi, Marcel Achtenhagen, Ruzhang Liu, and Michal Szostak. Metal-Free Transamidation of Secondary Amides via Selective N−C Cleavage under Mild Conditions. *Org. Lett*. 2017, **19**, 1614−1617.

18. Andreas P. Häring,Phillip Biallas,and Stefan F. Kirsch. An Unconventional Reaction of 2,2-Diazido Acylacetates with Amines. *Eur. J. Org. Chem*. 2017, 1526–1539

19. Agustín A. De la Fuente-Olvera, Oscar R. Suárez-Castillo, and Daniel Mendoza-Espinosa. Synthesis and Catalytic Applications of Palladium (II) Complexes Supported by Hydroxyl-Functionalized Triazolylidenes. Eur. J. Inorg. Chem. 2019, 4879–4886.

20. Shi-Meng Wang, Chuang Zhao, Xu Zhang and Hua-Li Qin. Clickable coupling of carboxylic acids and amines at room temperature mediated by SO_2F_2: a significant

breakthrough for the construction of amides and peptide linkages. Org. Biomol. Chem., 2019, 17, 4087–4101

21. Qinhua Hu, Lele Wang, Chen Wang, Yubin Wu, Zhengxin Ding and Rusheng Yuan. Ligand-free Pd(0)/SiO_2-catalyzed aminocarbonylation of aryl iodides to amides under atmospheric CO pressure. RSC Adv., 2017, 7, 37200–37207.
22. Andrea Ojeda-Porras, Alejandra Hernández-Santana and Diego Gamba-Sánchez. Direct amidation of carboxylic acids with amines under microwave irradiation using silica gel as a solid support. Green Chem., 2015, 17, 3157–3163.
23. Elisabetta Massolo, Margherita Pirola, Alessandra Puglisi, Sergio Rossi and Maurizio Benaglia. A one pot protocol to convert nitro-arenes into Naryl amides. RSC Adv., 2020, 10, 4040–4044.
24. Tomomi Yoshii, Saori Tsuzuki, Shunya Sakurai, Ryu Sakamoto, Julong Jiang, Miho Hatanaka, Akira Matsumoto and Keiji Maruok. N-Hydroxybenzimidazole as a structurally modifiable platform for N-oxyl radicals for direct C–H functionalization reactions. Chem. Sci., 2020, 11, 5772–5778
25. Hisanori Nambu, Kayoko Hata, Masato Matsugi, and Yasuyuki Kita. Efficient Synthesis of Thioesters and Amides from Aldehydes by Using an Intermolecular Radical Reaction in Water. Chem. Eur. J. 2005, 11, 719 – 727.
26. Diane S. W. Lim, Tedrick T. S. Lew, and Yugen Zhang. Direct Amidation of N‑Boc- and N‑Cbz-Protected Amines via Rhodium-Catalyzed Coupling of Arylboroxines and Carbamates. Org. Lett. 2015, 17, 6054−6057.
27. Soonho Hwang, Sang Yoon Choi, Jin Hee Lee, Shinae Kim, Jinkyung In, Sang Keun Ha, Eunjung Lee, Tae-Yoon Kim, Sun Yeou Kim, Sun Choi, Sanghee Kim.

Identification of a potent and noncytotoxic inhibitor of melanin production. Bioorganic & Medicinal Chemistry 18 (2010) 5602–5609.

28. Jason D. Williams, William J. Kerr, Stuart G. Leach, and David M. Lindsay. A Practical and General Amidation Method from Isocyanates Enabled by Flow Technology. Angew. Chem. Int. Ed. 2018, 57, 12126 –12130.
29. Khushbu P. Patel, Eknath M. Gayakwad and Ganapati S. Shankarling. Graphene oxide: a convenient metal-free carbocatalyst for facilitating amidation of esters with amines. New J. Chem., 2020, 44, 2661—2668.
30. Shengmei Guo, Sen Li, Wenjie Yan, Zhibin Liang, Zhengjiang Fu and Hu Cai. Environmentally sustainable production and application of acyl phosphates. Green Chem., 2020, 22, 7343–7347.
31. Sirilak Wangngae, Chuthamat Duangkamol, Mookda Pattarawarapan and Wong Phakhodee. Significance of reagent addition sequence in the amidation of carboxylic acids mediated by PPh_3 and I_2. RSC Adv., 2015, 5, 25789–25793

6. NMR Spectra

***N*-phenylbenzamide (3a)**

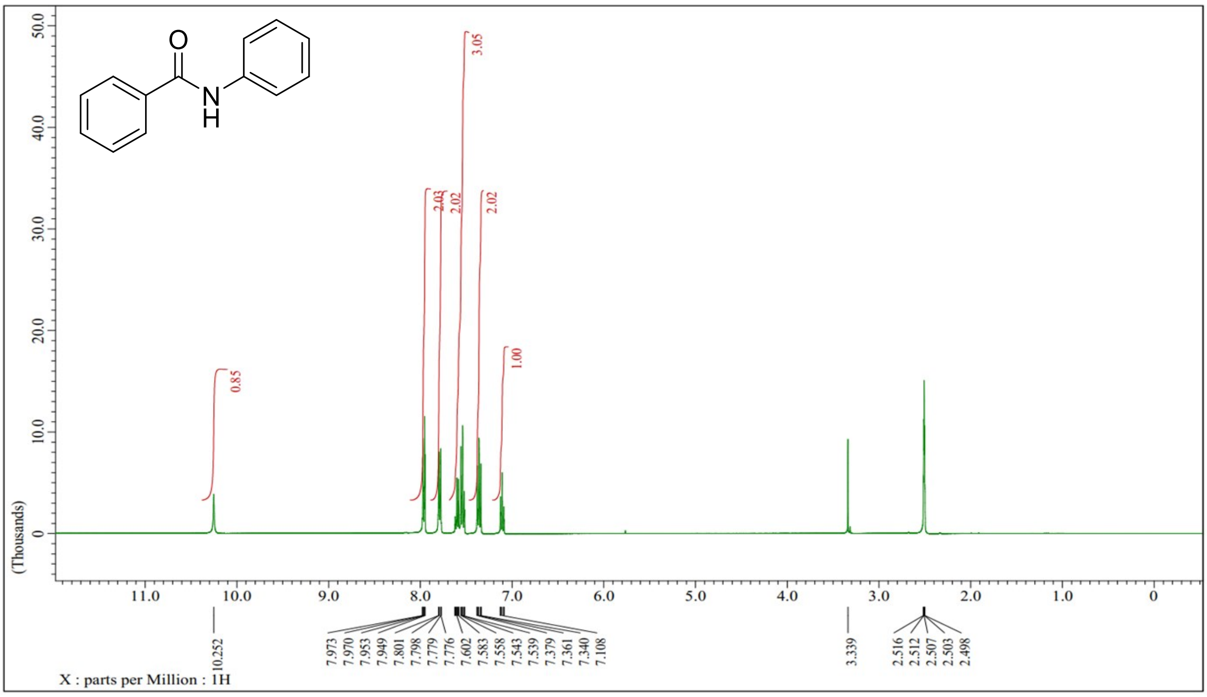

^{1}H NMR spectrum of *N*-phenylbenzamide (3a)

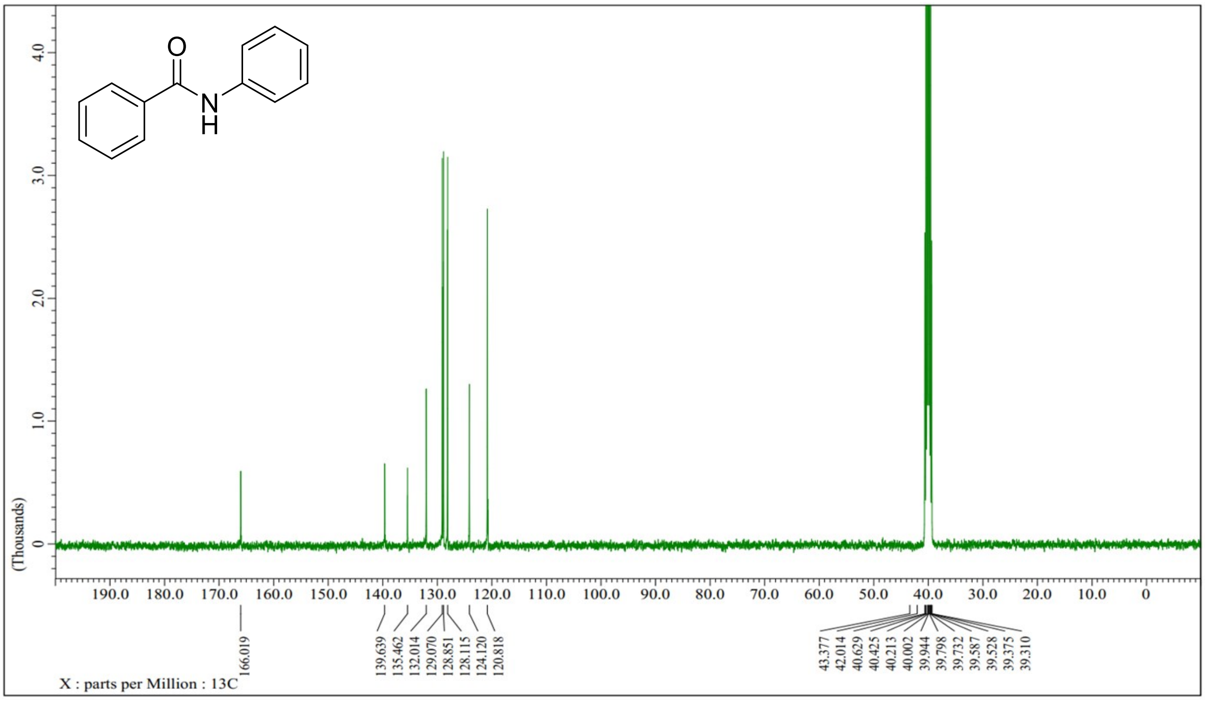

^{13}C NMR spectrum of *N*-phenylbenzamide (3a)

4-methyl-*N*-phenylbenzamide (3b)

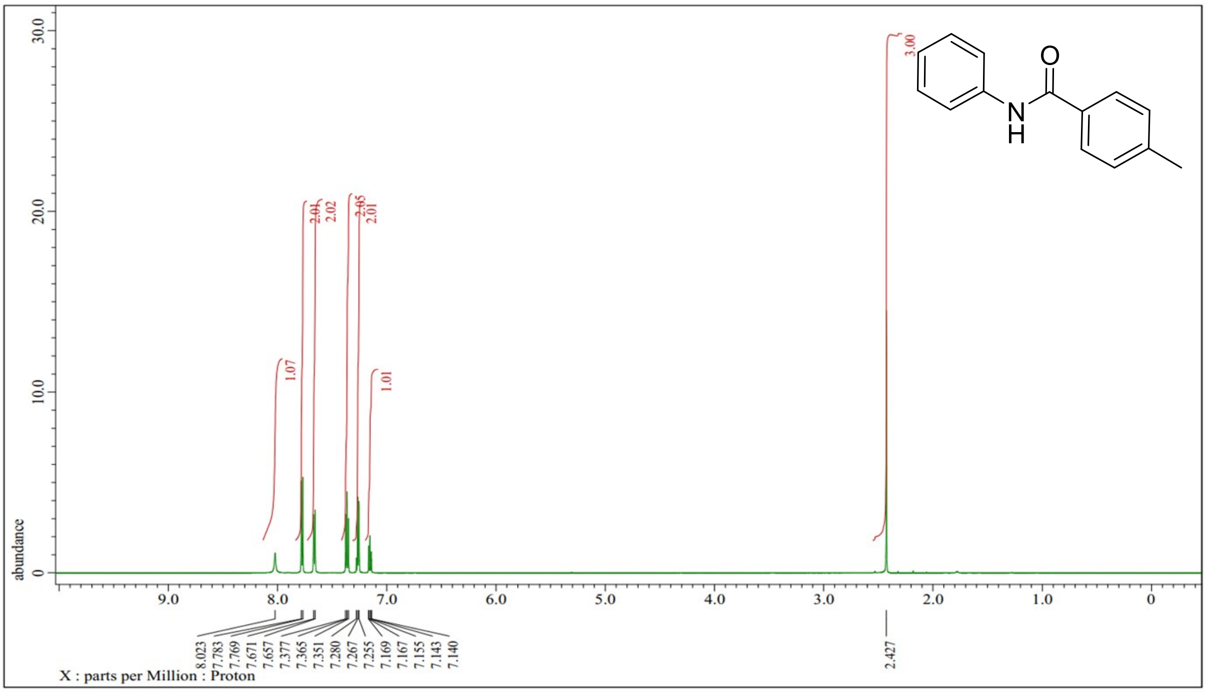

^{1}H NMR spectrum of 4-methyl-*N*-phenylbenzamide (3b)

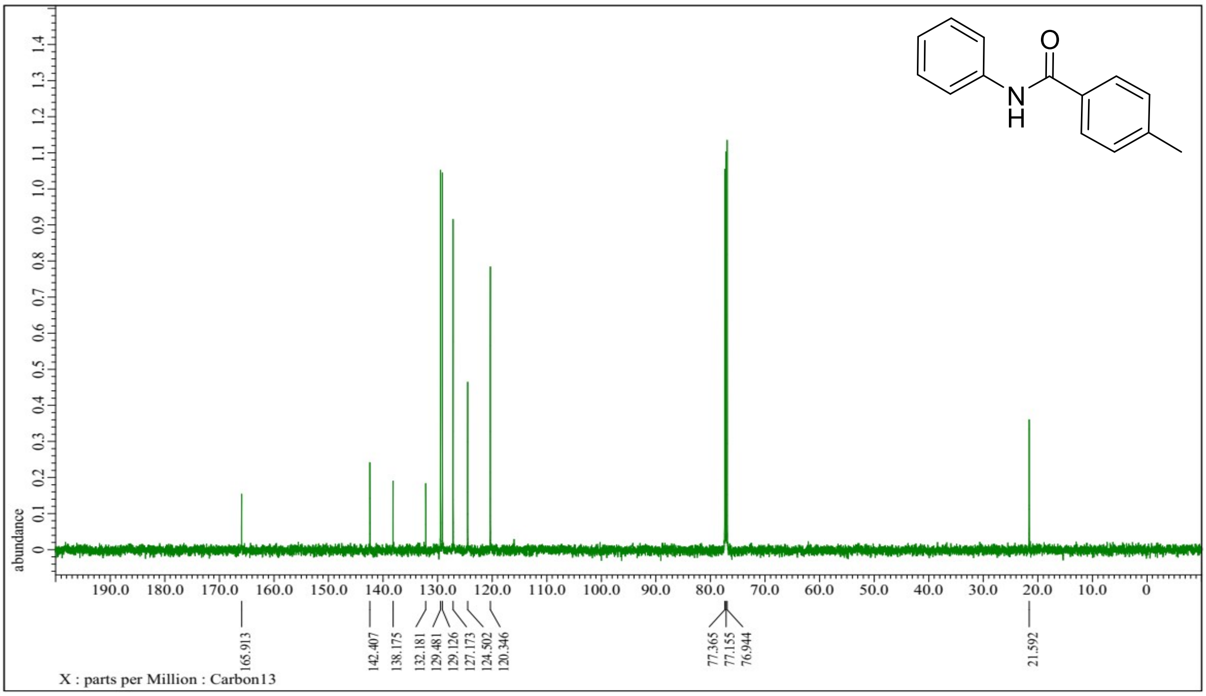

^{13}C NMR spectrum of 4-methyl-*N*-phenylbenzamide (3b)

3,5-dimethyl-*N*-phenylbenzamide (3c)

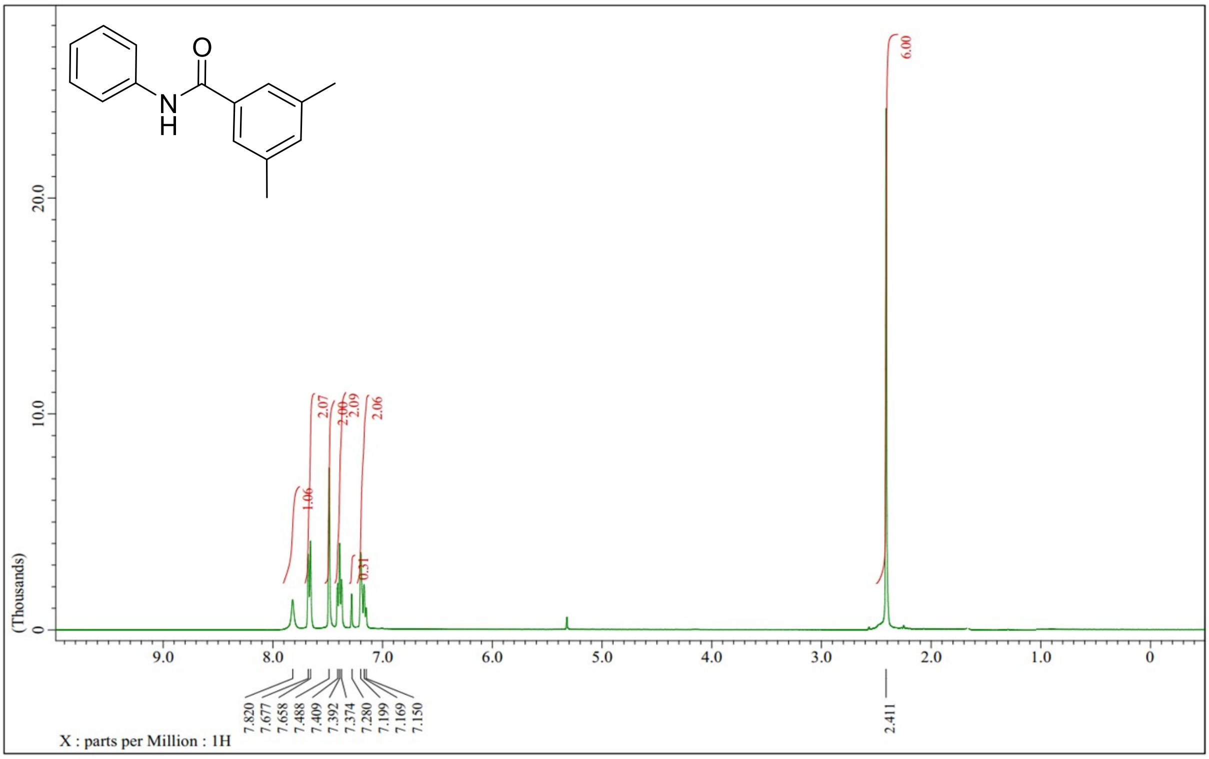

^{1}H NMR spectrum of 3,5-dimethyl-*N*-phenylbenzamide (3c)

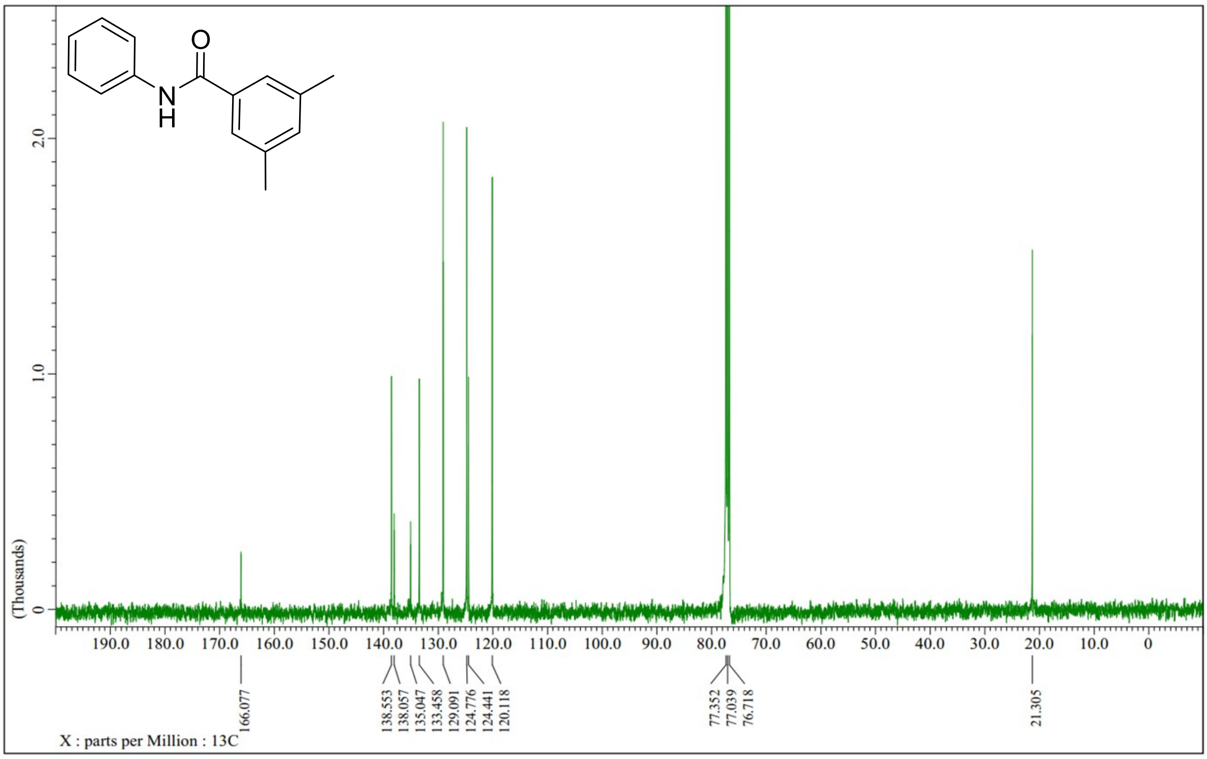

^{13}C NMR spectrum of 3,5-dimethyl-*N*-phenylbenzamide (3c)

4-methoxy-*N*-phenylbenzamide (3d)

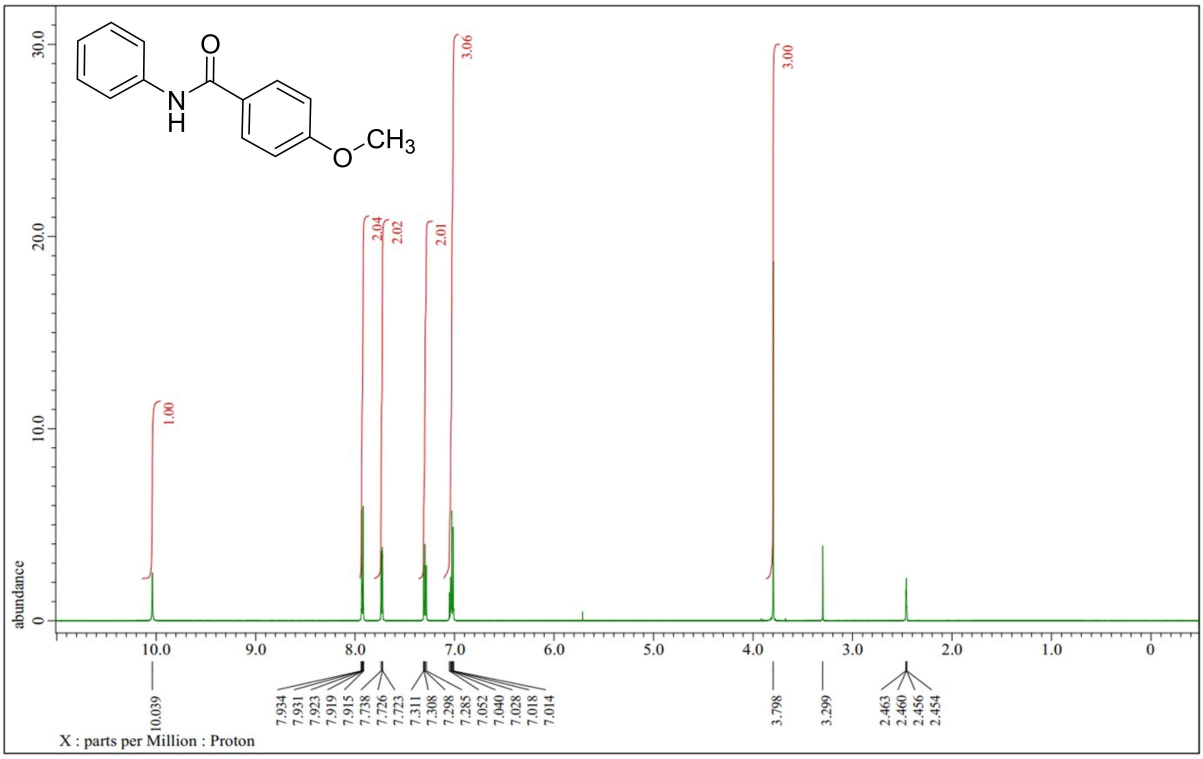

^{1}H NMR spectrum of 4-methoxy-*N*-phenylbenzamide (3d)

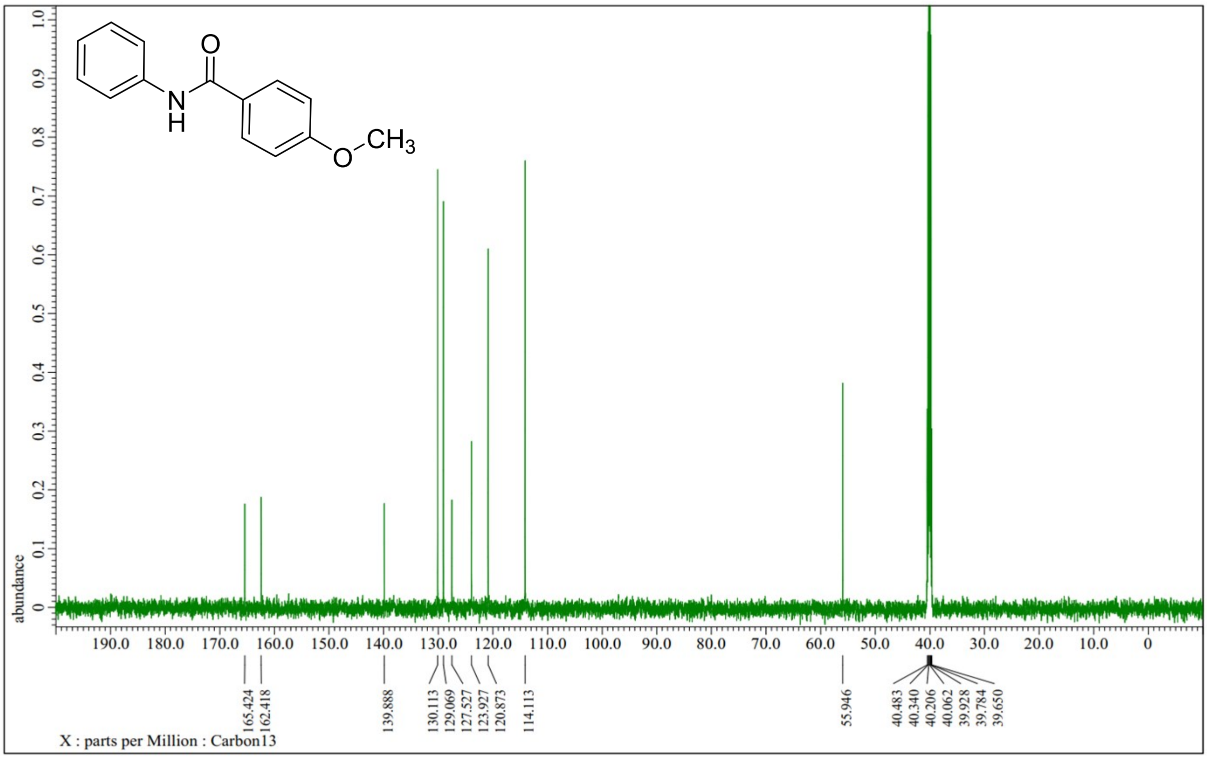

^{13}C NMR spectrum of 4-methoxy-*N*-phenylbenzamide (3d)

4-chloro-*N*-phenylbenzamide (3e)

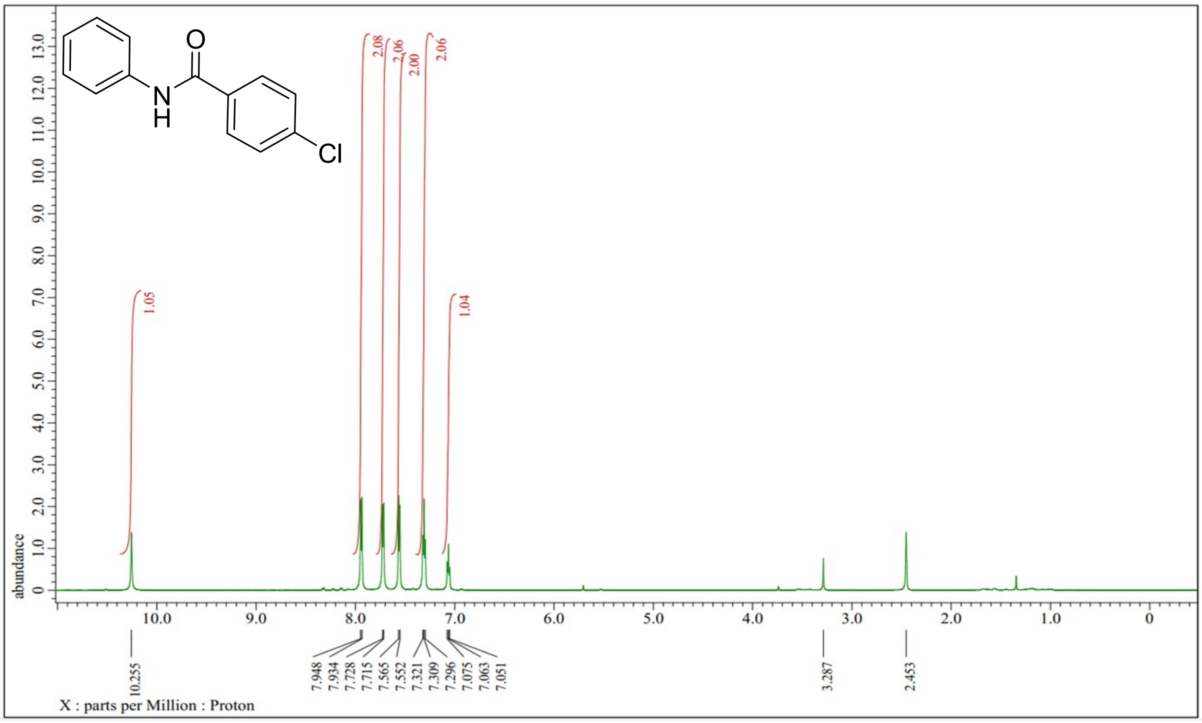

^{1}H NMR spectrum of 4-chloro-*N*-phenylbenzamide (3e)

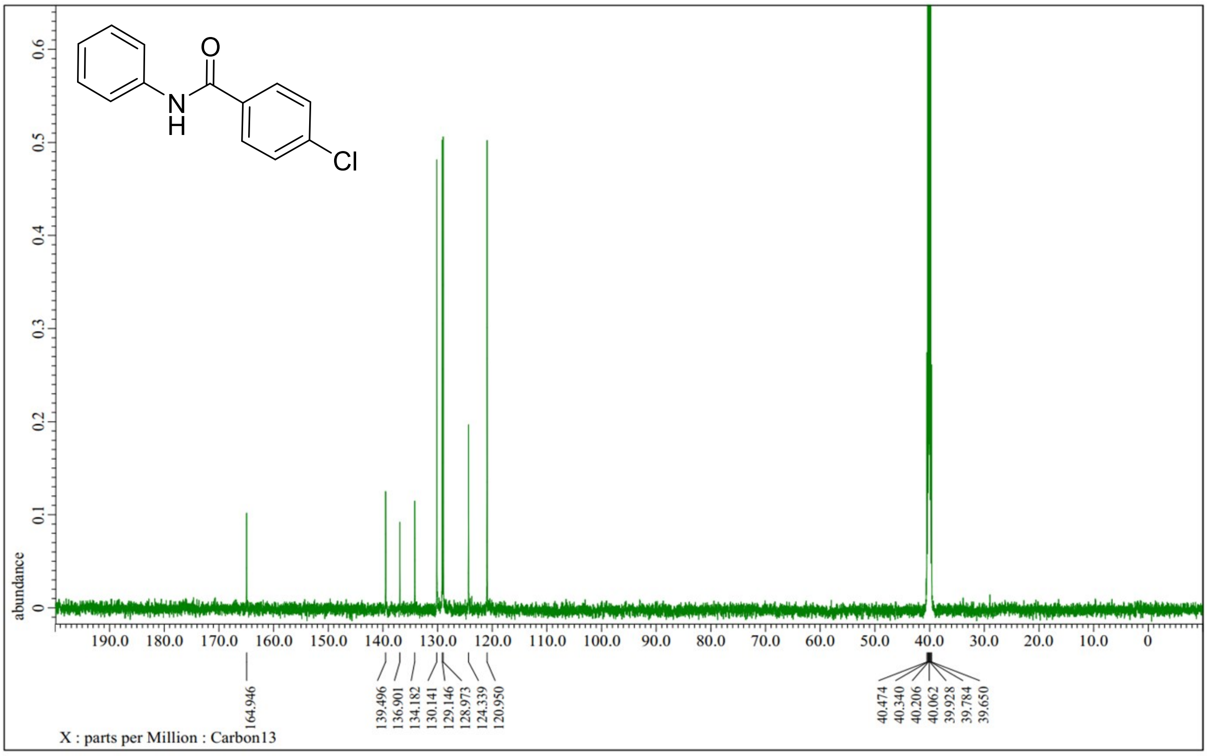

^{13}C NMR spectrum of 4-chloro-*N*-phenylbenzamide (3e)

N-phenyl-4-(trifluoromethyl)benzamide (3f)

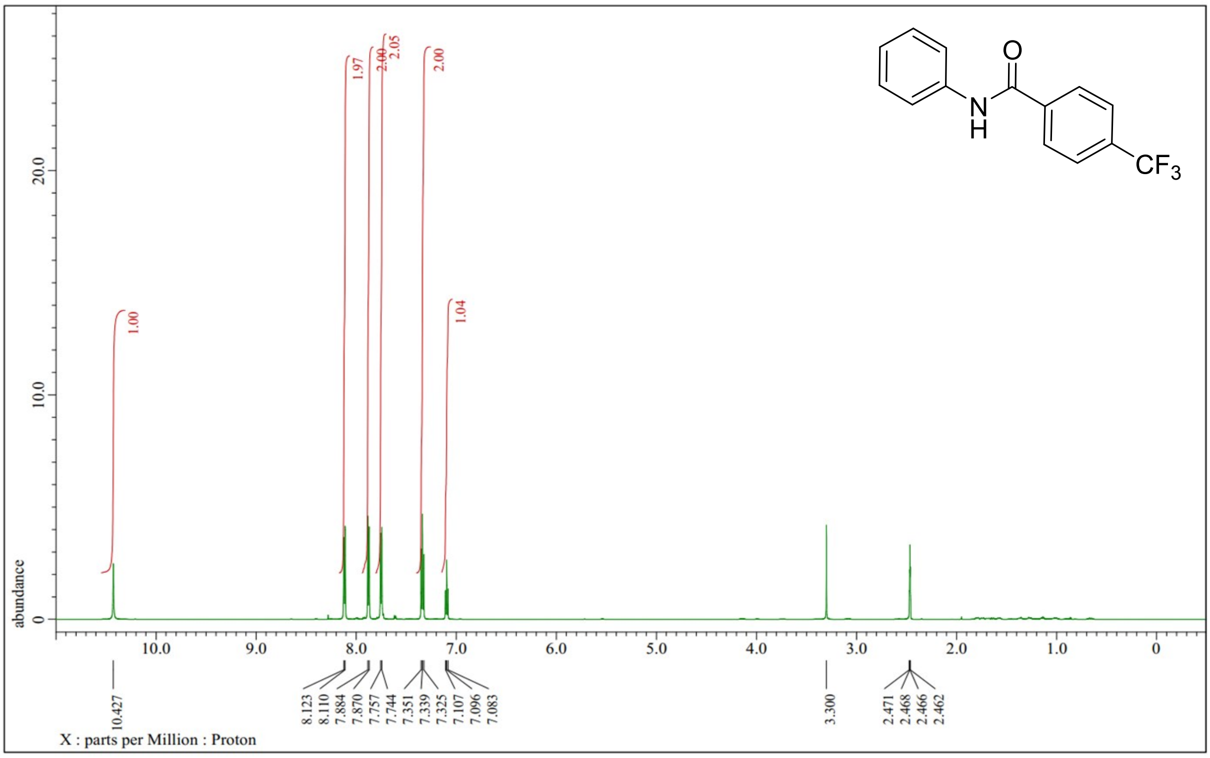

^{1}H NMR spectrum of *N*-phenyl-4-(trifluoromethyl)benzamide (3f)

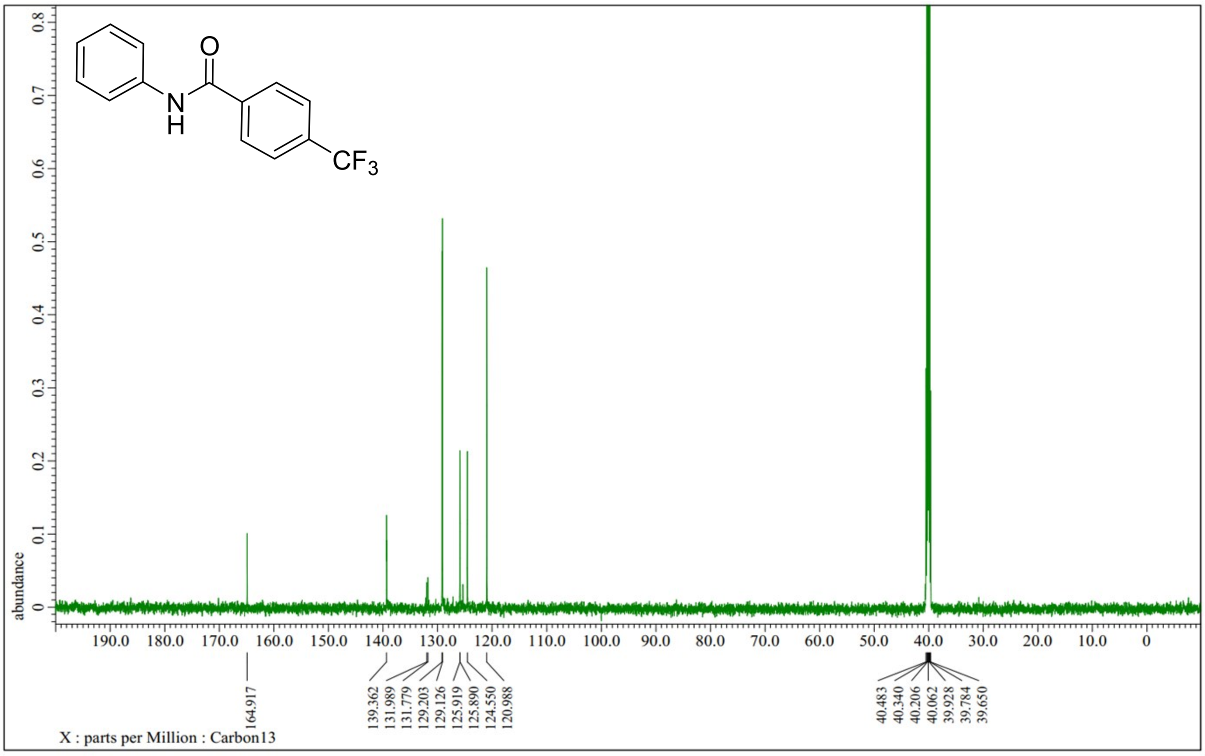

^{13}C NMR spectrum of *N*-phenyl-4-(trifluoromethyl)benzamide (3f)

***N*-phenylbutyramide (3g)**

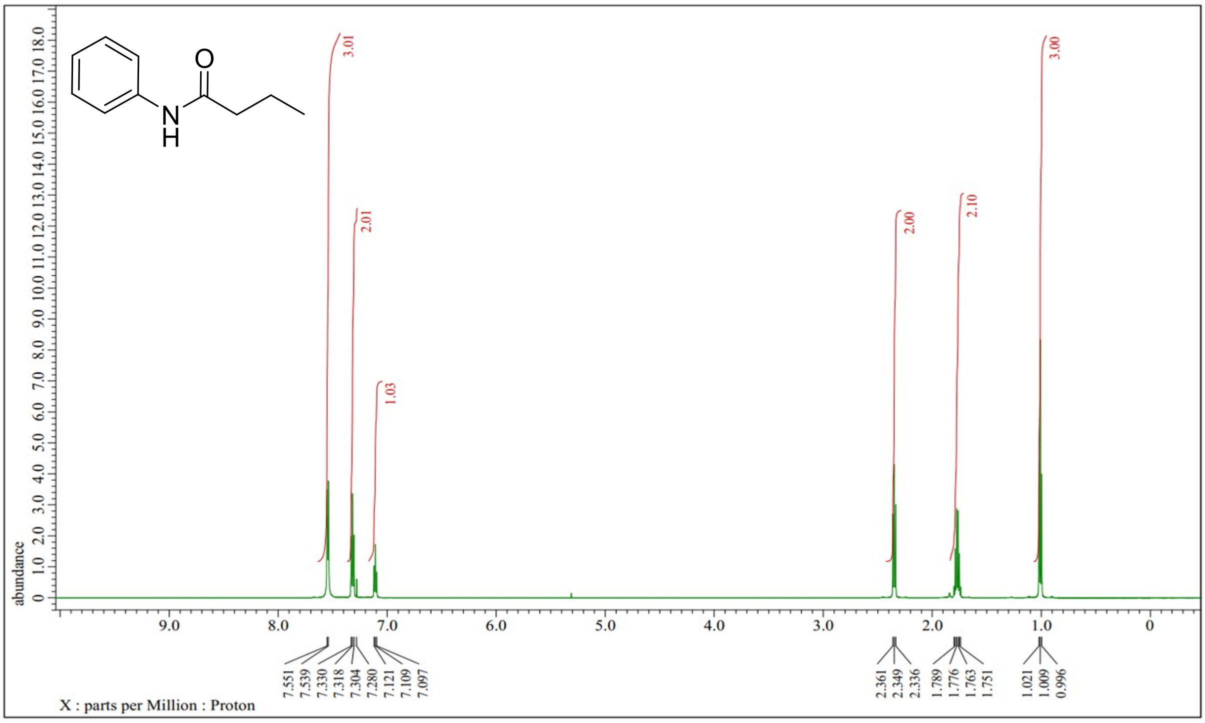

^{1}H NMR spectrum of *N*-phenylbutyramide (3g)

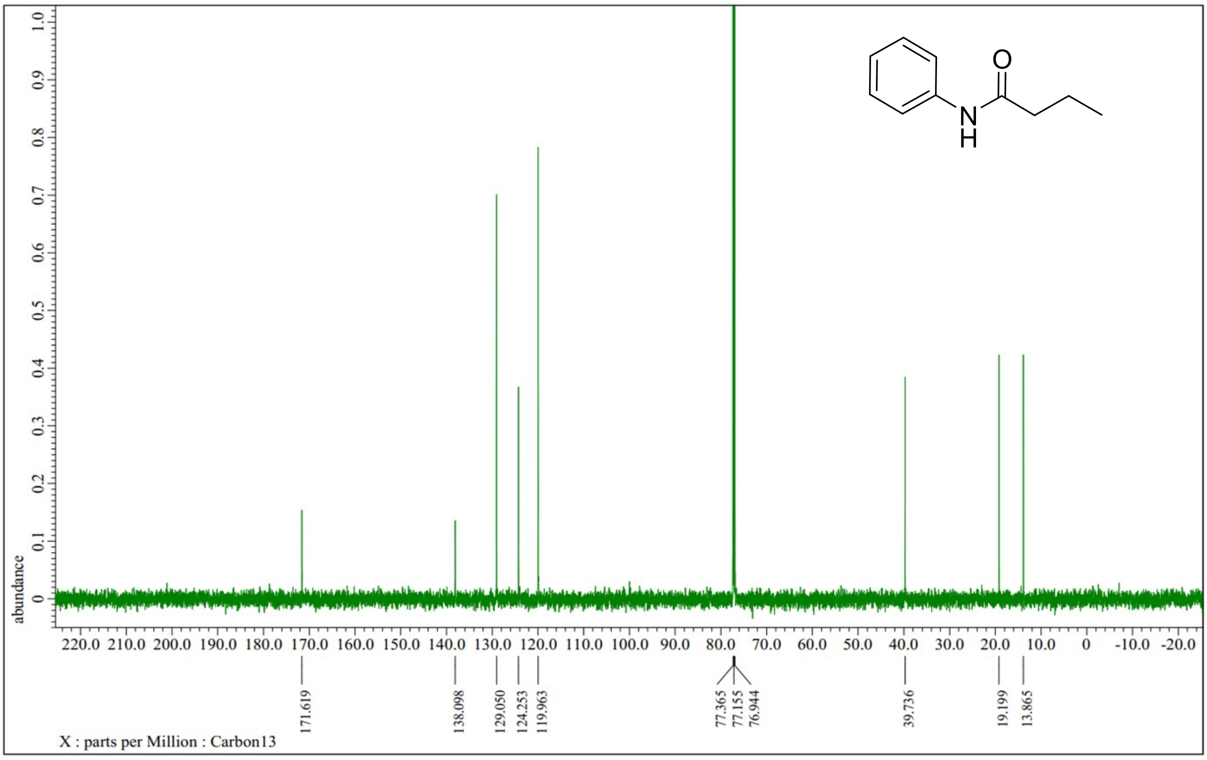

^{13}C NMR spectrum of *N*-phenylbutyramide (3g)

***N*-phenylcyclohexanecarboxamide (3h)**

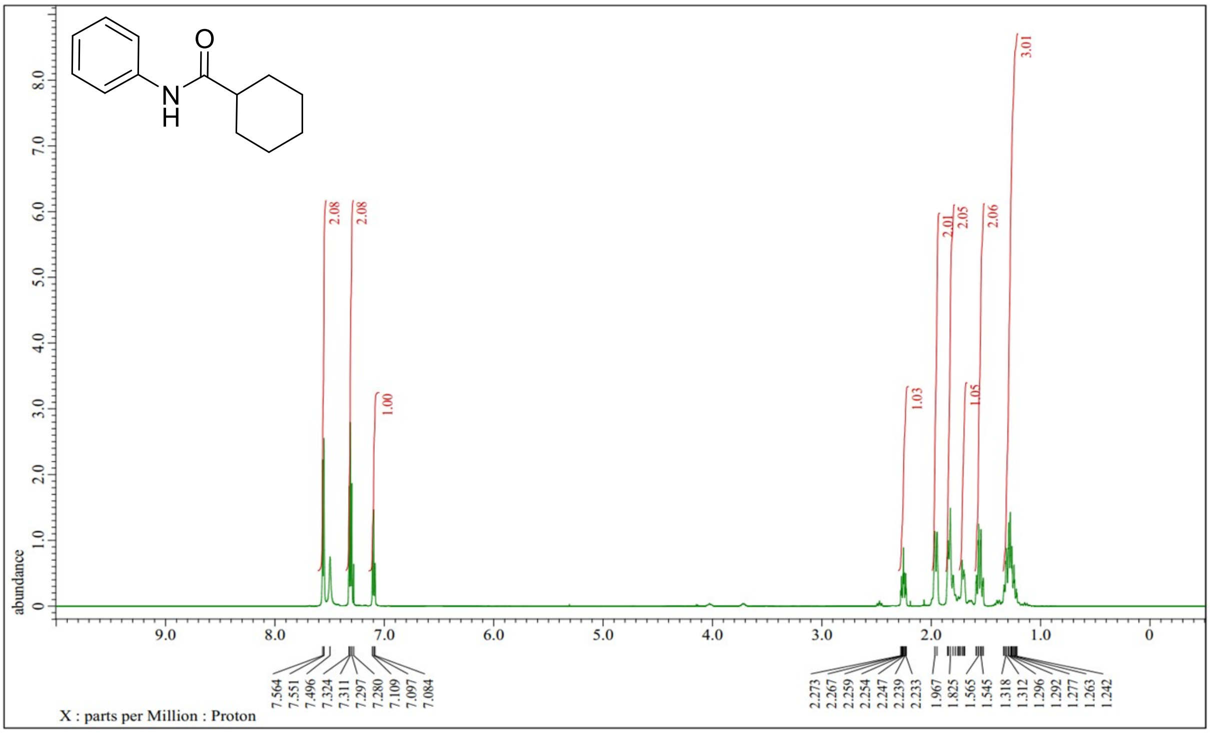

^{1}H NMR spectrum of *N*-phenylcyclohexanecarboxamide (3h)

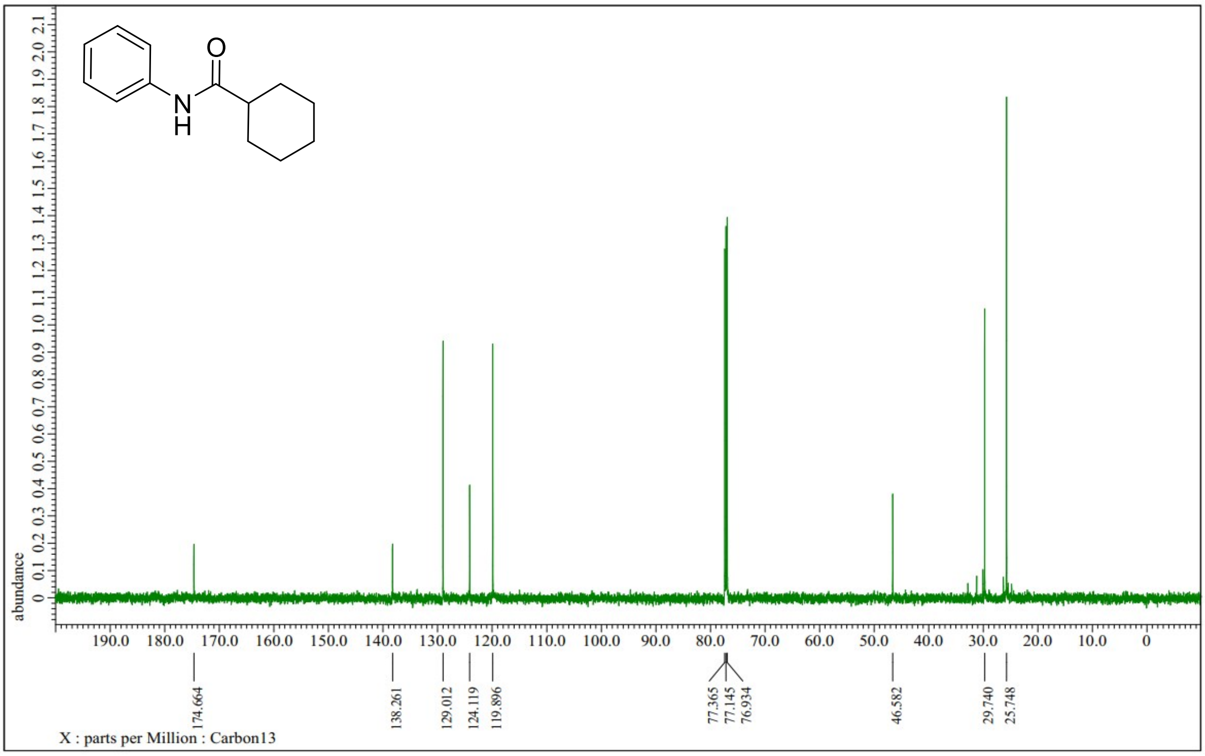

^{13}C NMR spectrum of *N*-phenylcyclohexanecarboxamide (3h)

_N_-phenyl-1-naphthamide (3i)

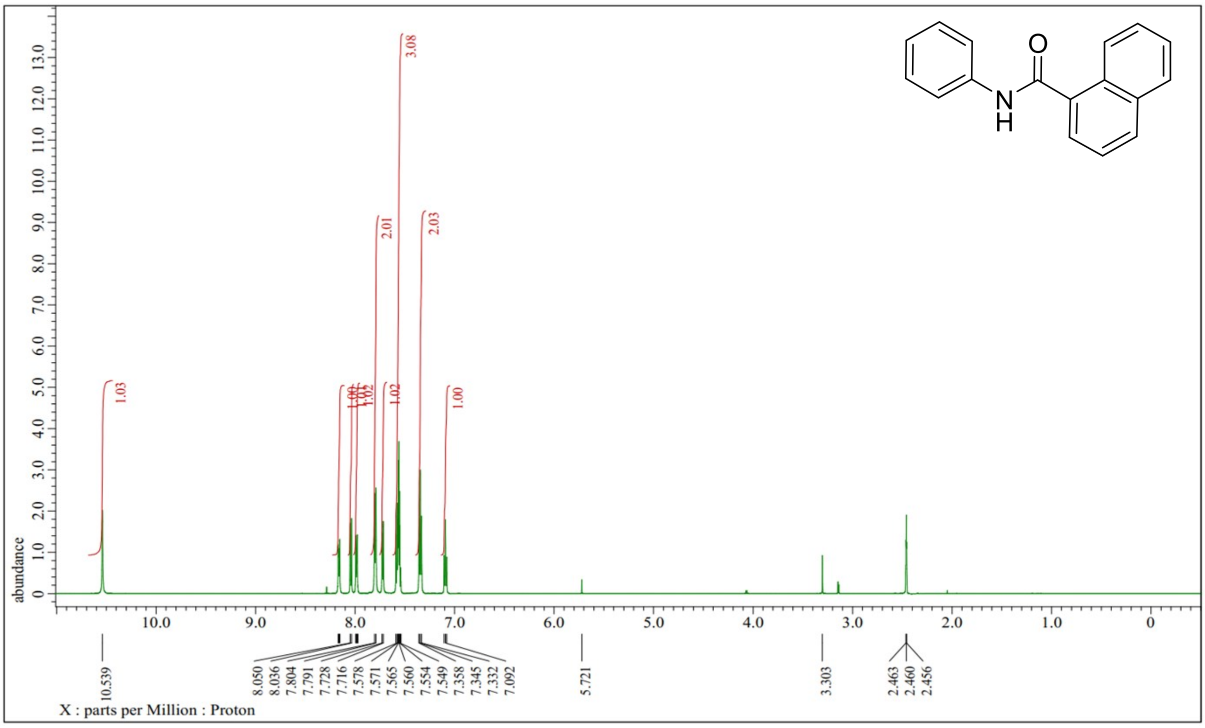

^{1}H NMR spectrum of _N_-phenyl-1-naphthamide (3i)

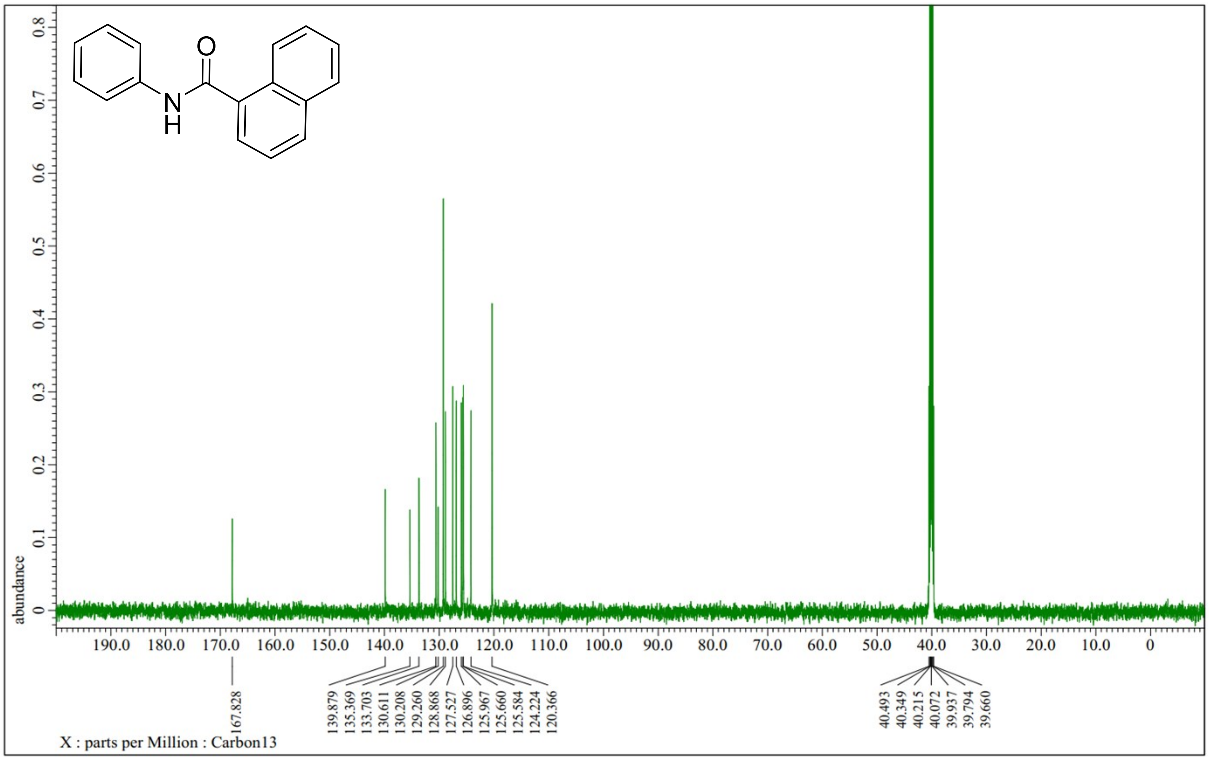

^{13}C NMR spectrum of _N_-phenyl-1-naphthamide (3i)

***N*-(3,5-dimethylphenyl)benzamide (3j)**

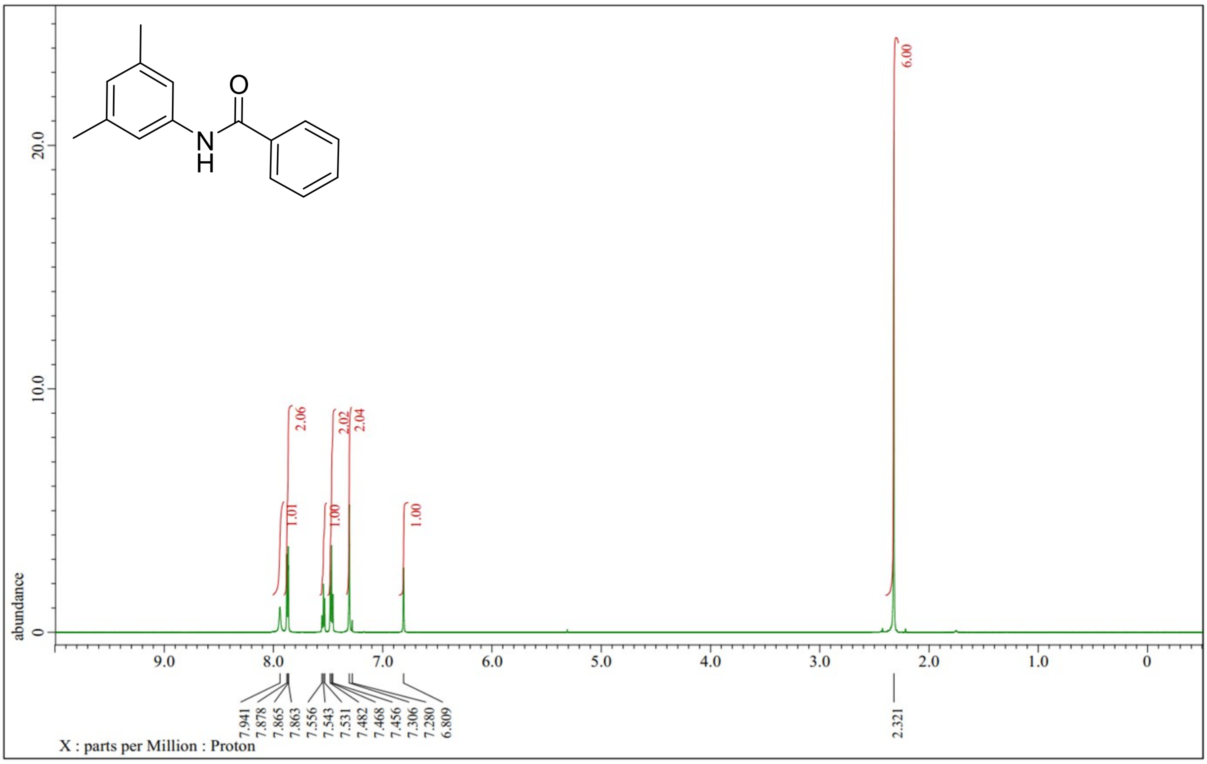

^{1}H NMR spectrum of *N*-(3,5-dimethylphenyl)benzamide (3j)

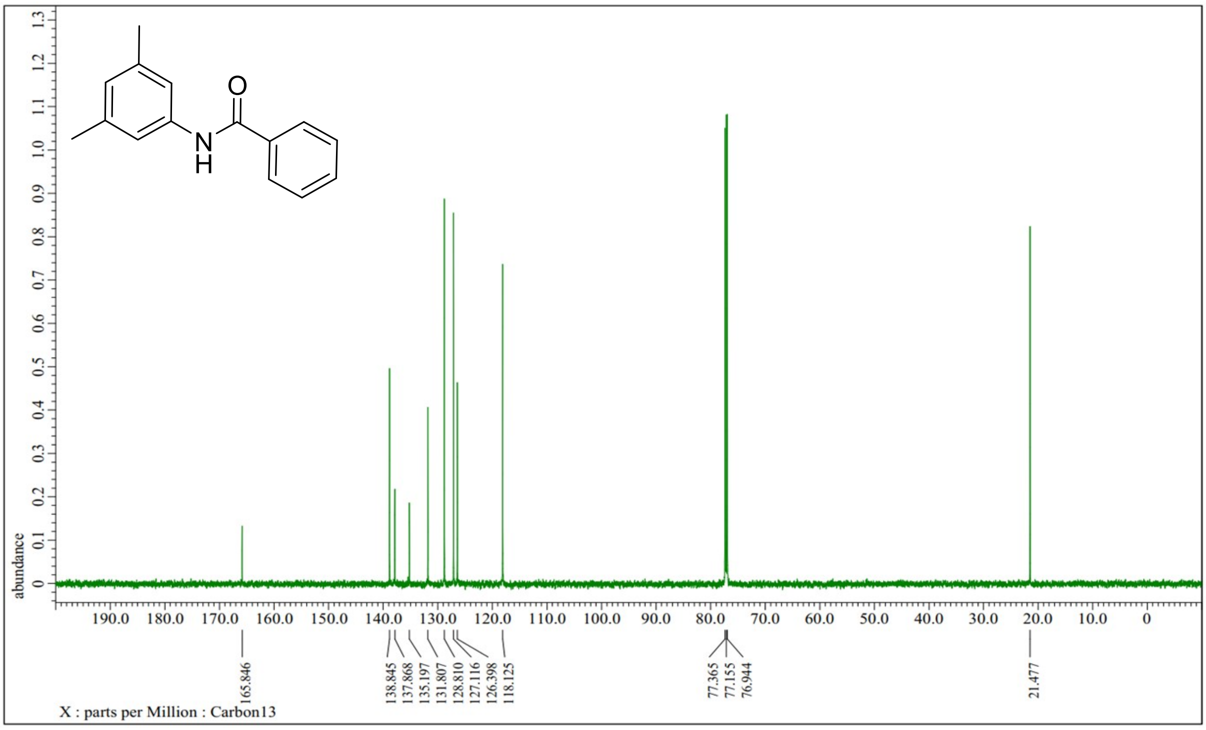

^{13}C NMR spectrum of *N*-(3,5-dimethylphenyl)benzamide (3j)

N-(3,5-dimethylphenyl)-1-naphthamide (3k)

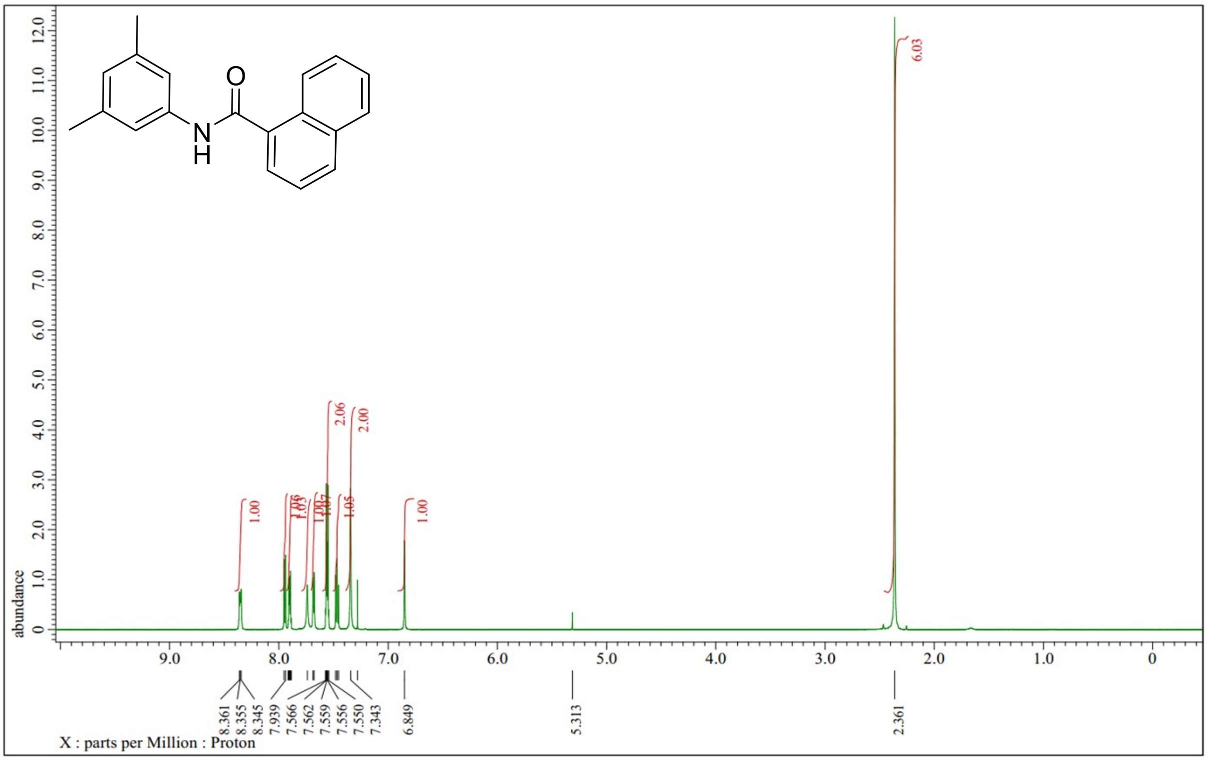

^{1}H NMR spectrum of *N*-(3,5-dimethylphenyl)-1-naphthamide (3k)

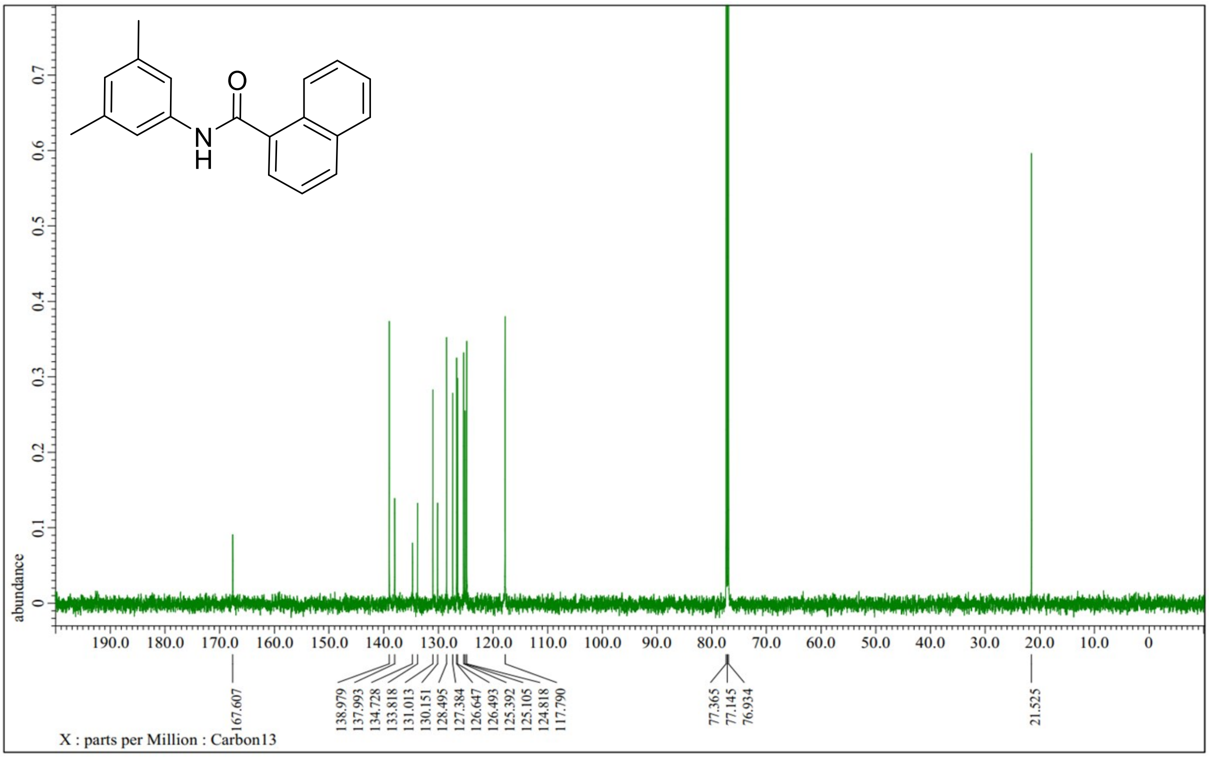

^{13}C NMR spectrum of *N*-(3,5-dimethylphenyl)-1-naphthamide (3k)

N-(3,5-dimethylphenyl)-2,4,6-trimethylbenzamide (3l)

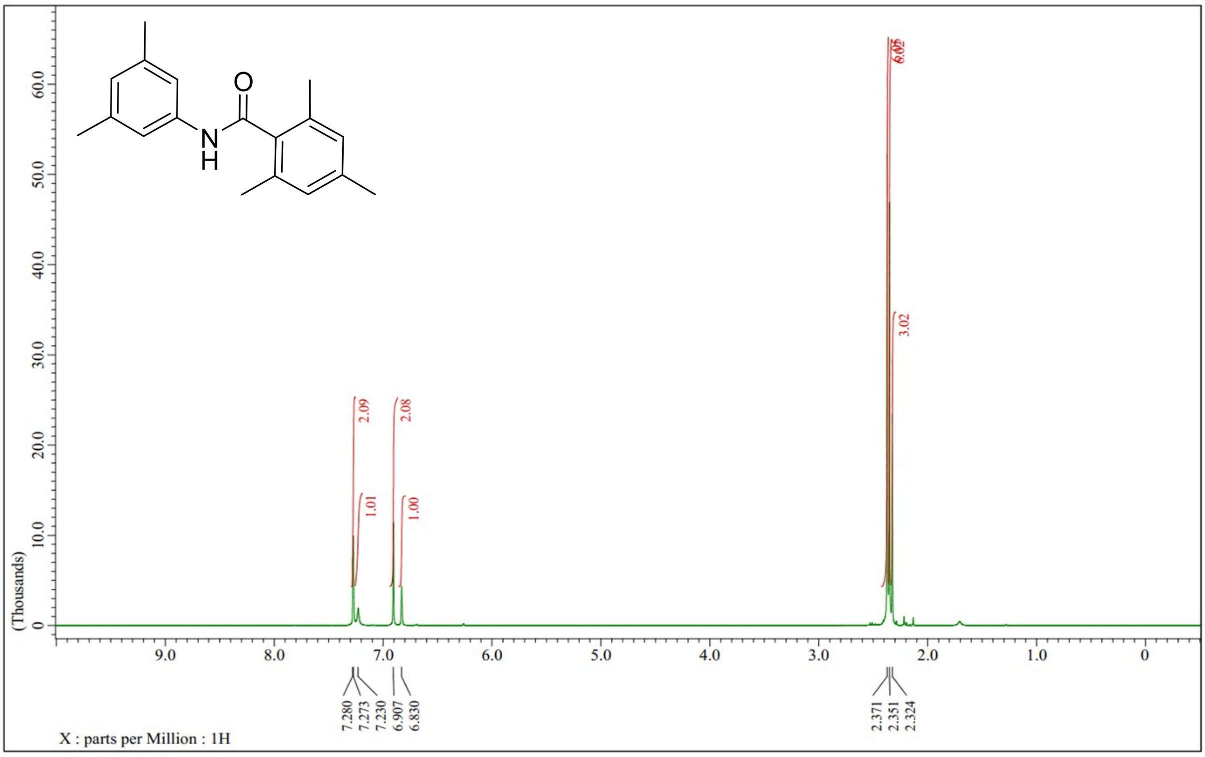

^{1}H NMR spectrum of *N*-(3,5-dimethylphenyl)-2,4,6-trimethylbenzamide (3l)

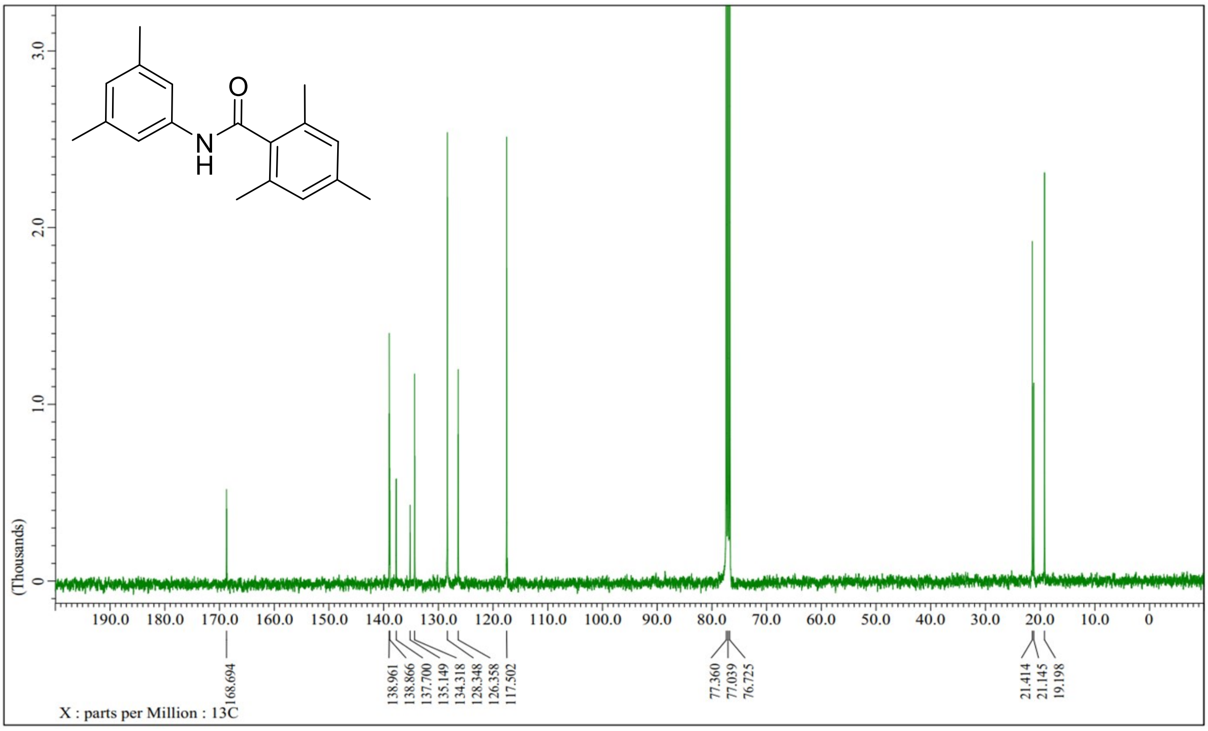

^{13}C NMR spectrum of *N*-(3,5-dimethylphenyl)-2,4,6-trimethylbenzamide (3l)

3-methyl-*N*-(*p*-tolyl)butanamide (3m)

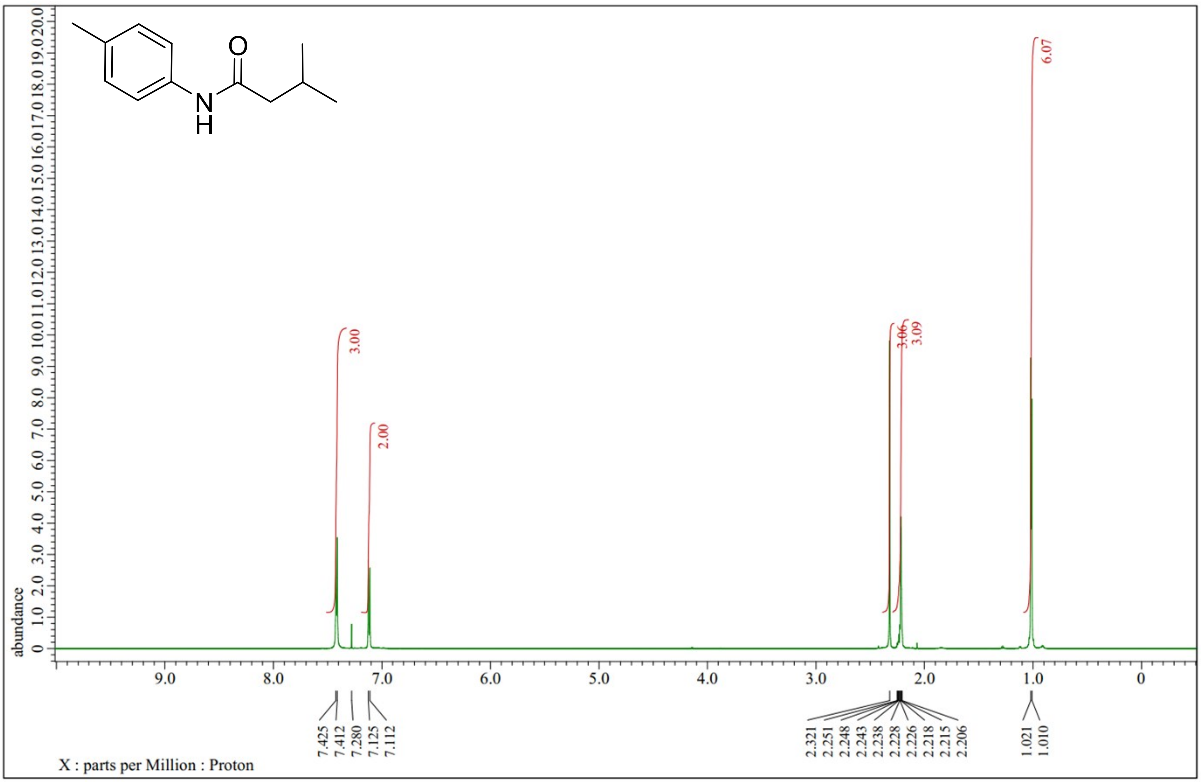

^{1}H NMR spectrum of 3-methyl-*N*-(*p*-tolyl)butanamide (3m)

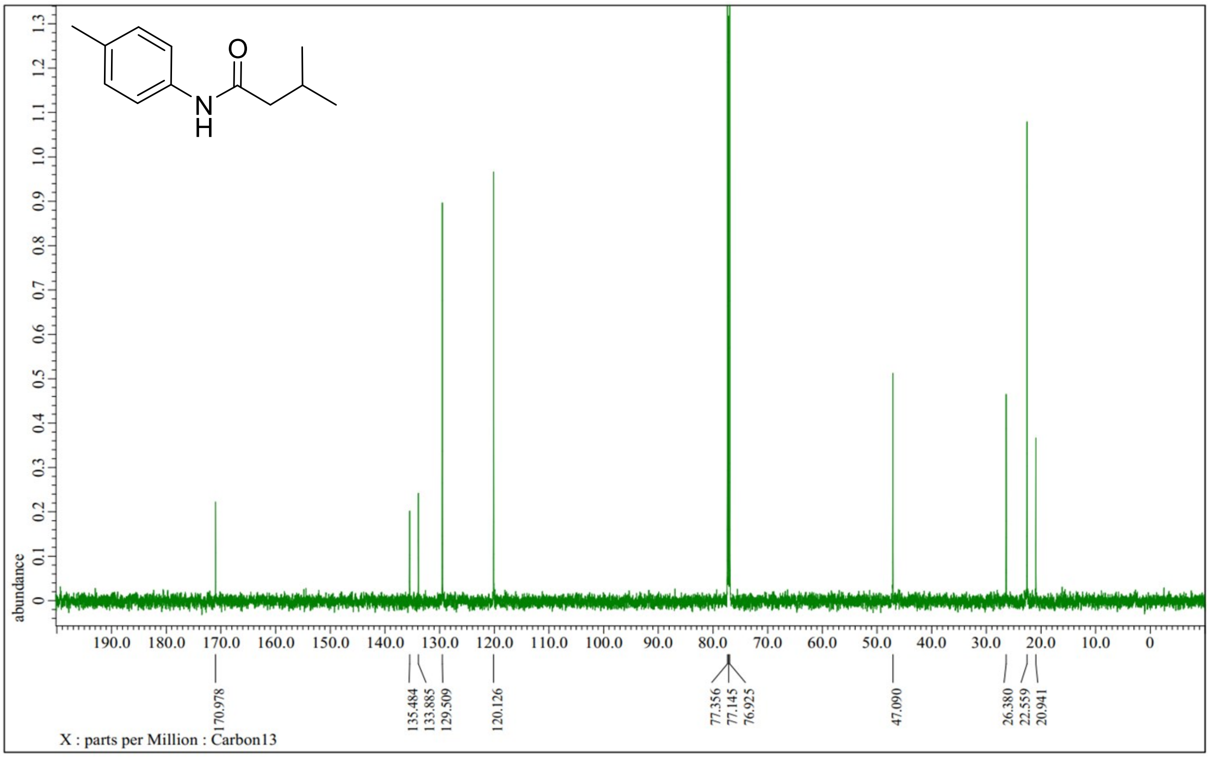

^{13}C NMR spectrum of 3-methyl-*N*-(*p*-tolyl)butanamide (3m)

3-phenyl-*N*-(*p*-tolyl)propanamide (3n)

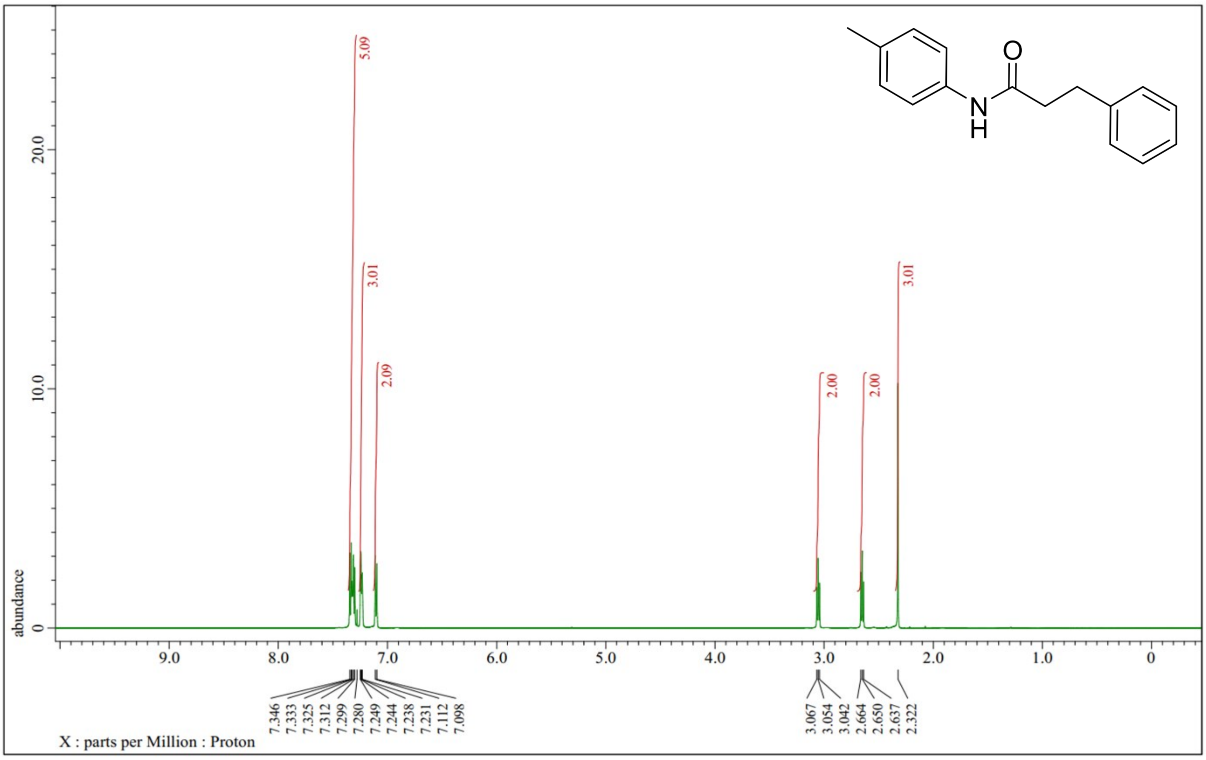

^{1}H NMR spectrum of 3-phenyl-*N*-(*p*-tolyl)propanamide (3n)

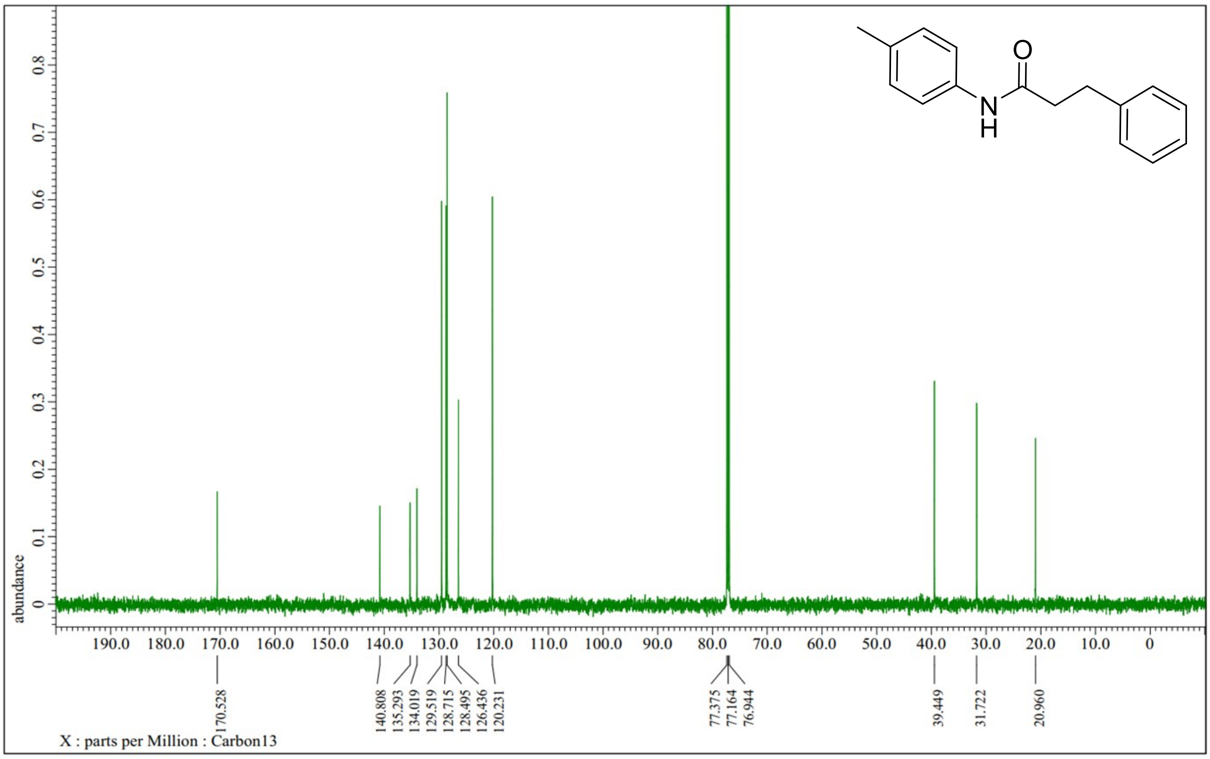

^{13}C NMR spectrum of 3-phenyl-*N*-(*p*-tolyl)propanamide (3n)

***N*-(4-chlorophenyl)benzamide (3o)**

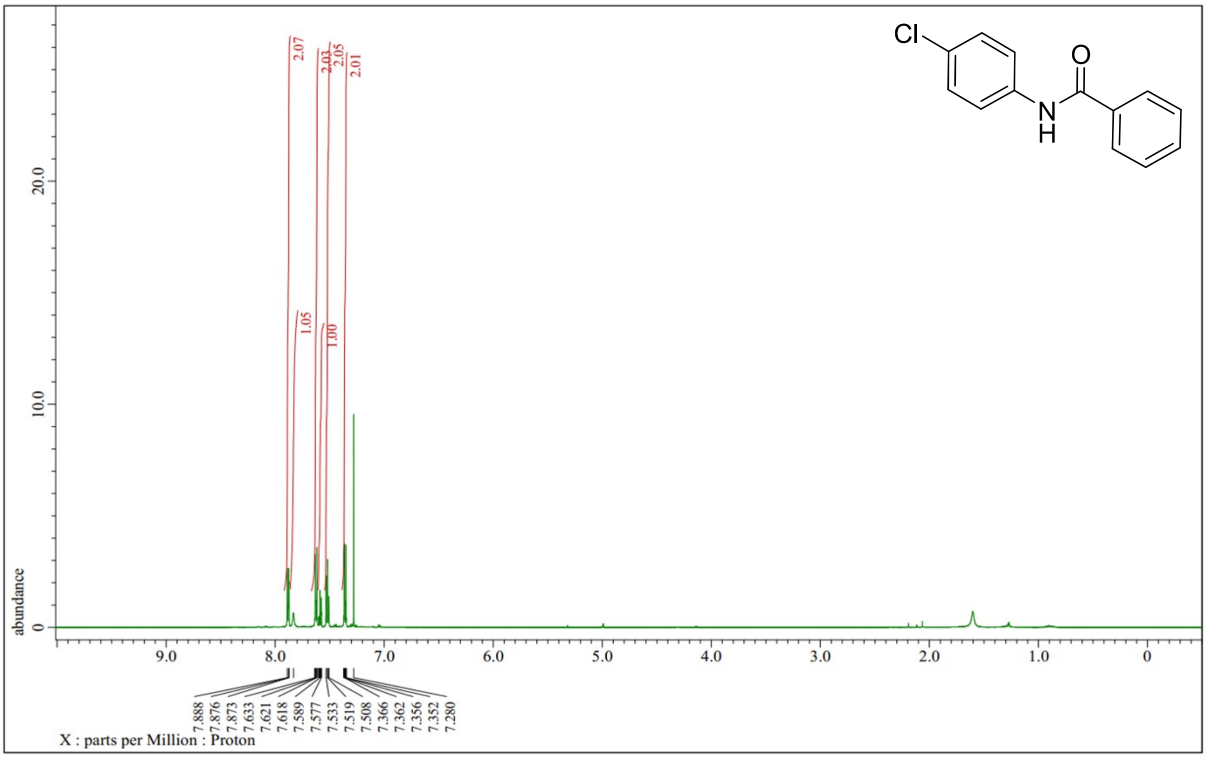

^{1}H NMR spectrum of *N*-(4-chlorophenyl)benzamide (3o)

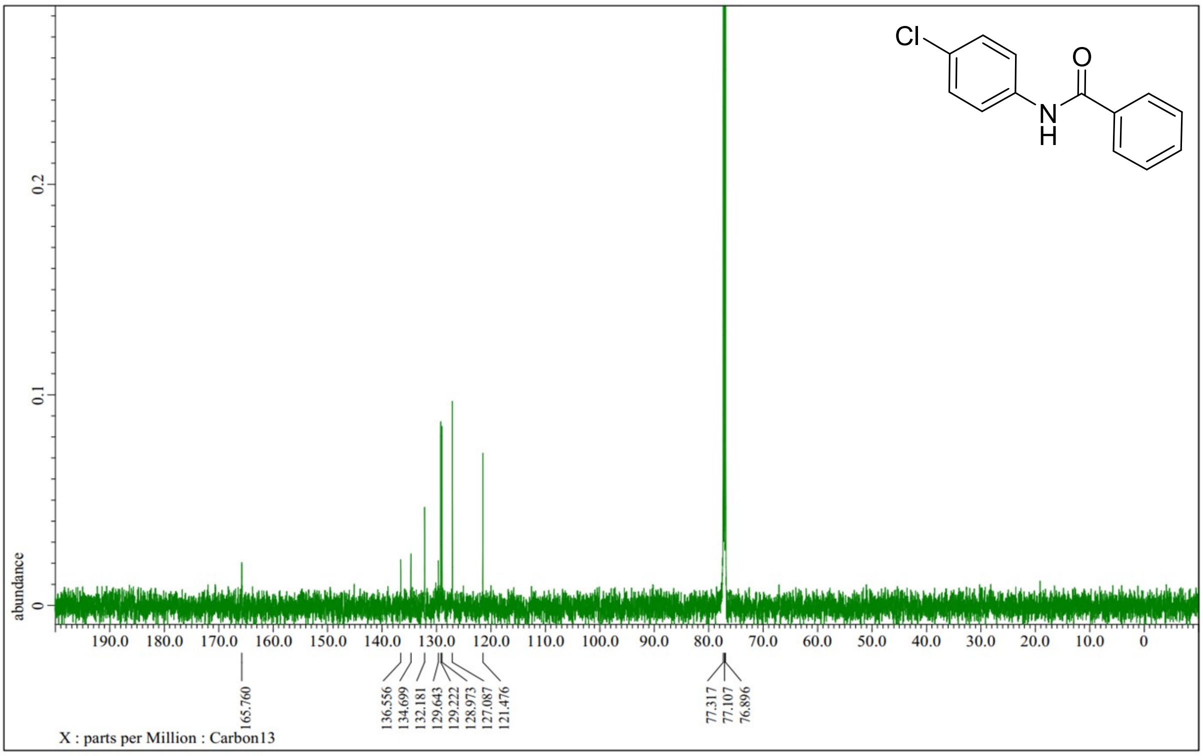

^{13}C NMR spectrum of *N*-(4-chlorophenyl)benzamide (3o)

***N*-(4-chlorophenyl)butyramide (3p)**

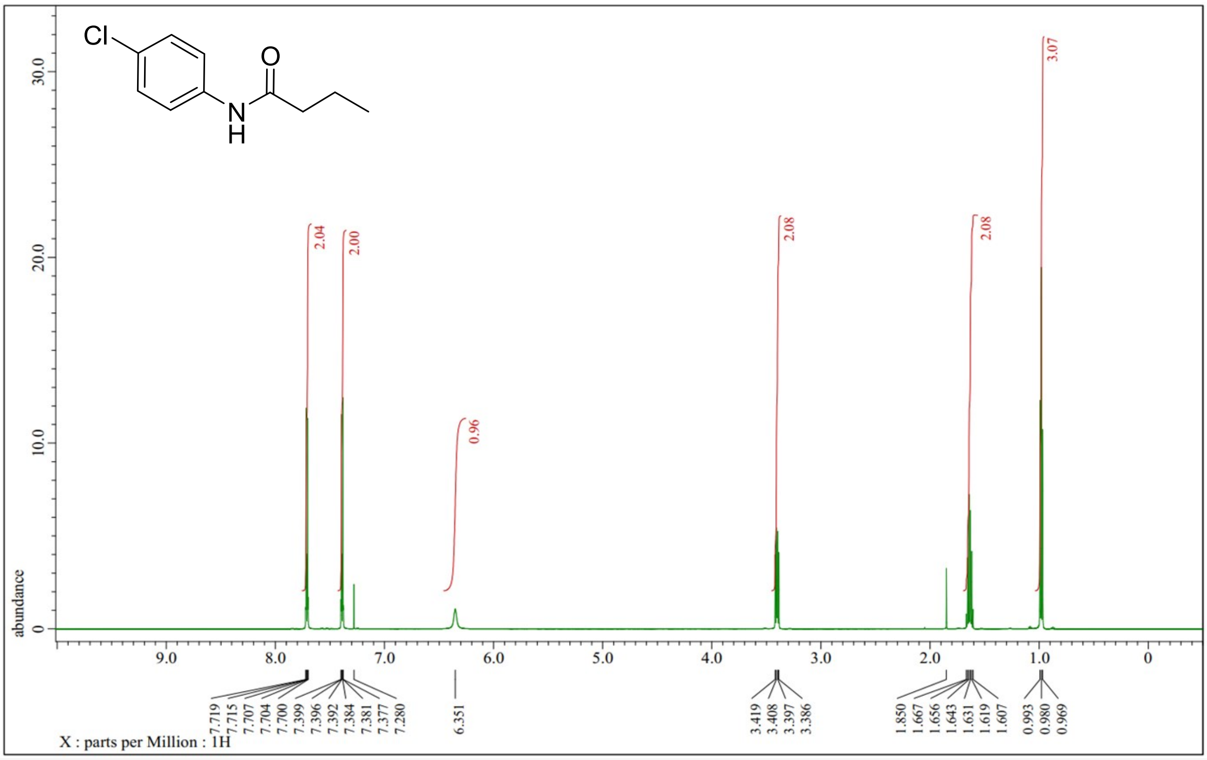

^{1}H NMR spectrum of *N*-(4-chlorophenyl)butyramide (3p)

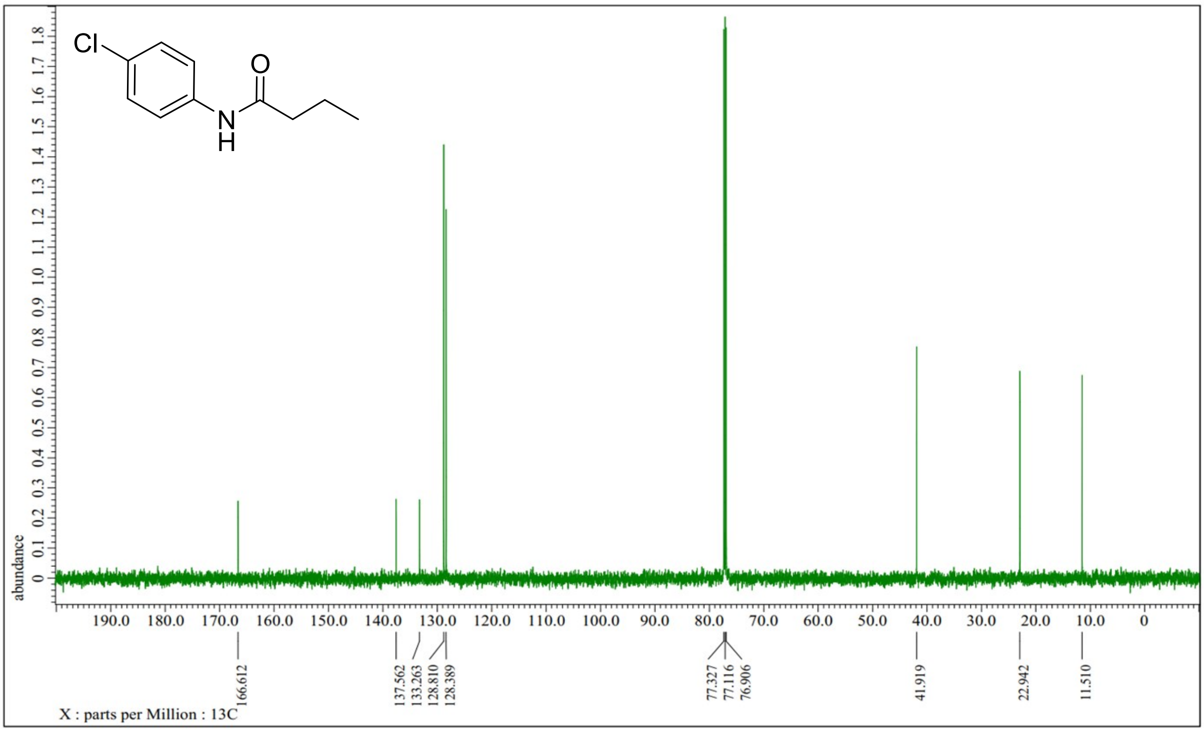

^{13}C NMR spectrum of *N*-(4-chlorophenyl)butyramide (3p)

***N*-(4-cyanophenyl)-3-methylbutanamide (3q)**

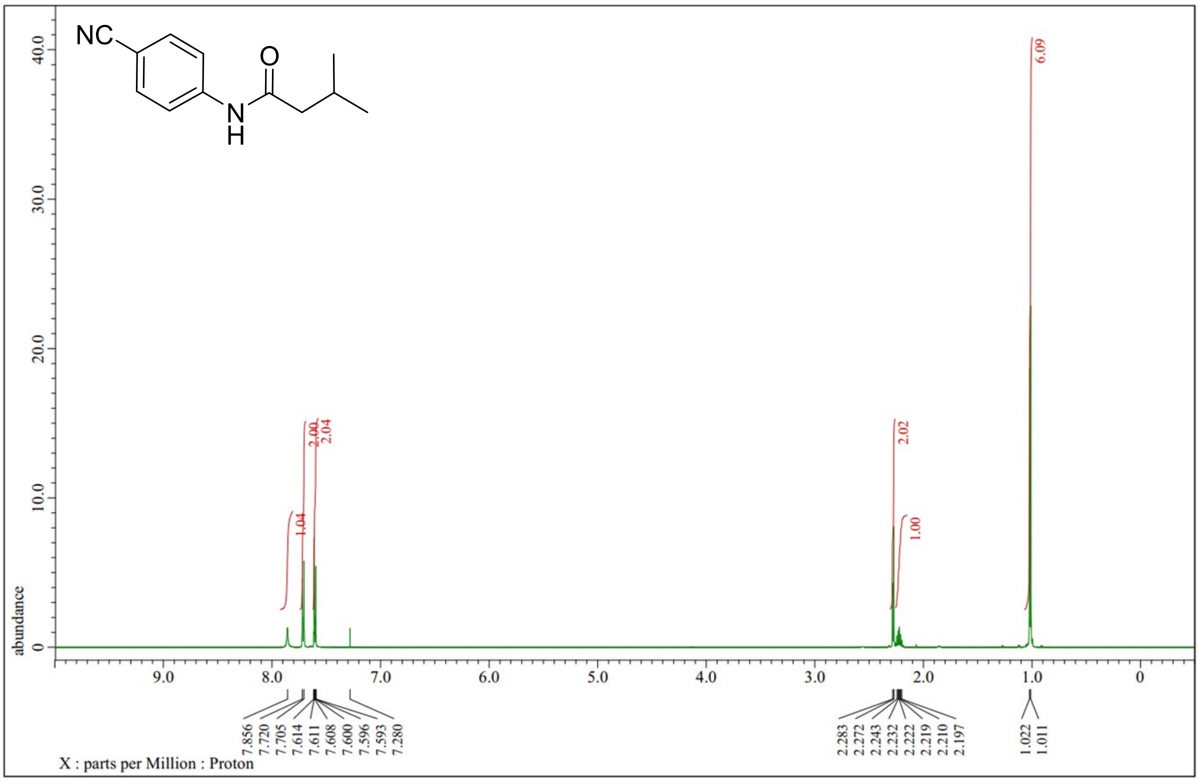

^{1}H NMR spectrum of *N*-(4-cyanophenyl)-3-methylbutanamide (3q)

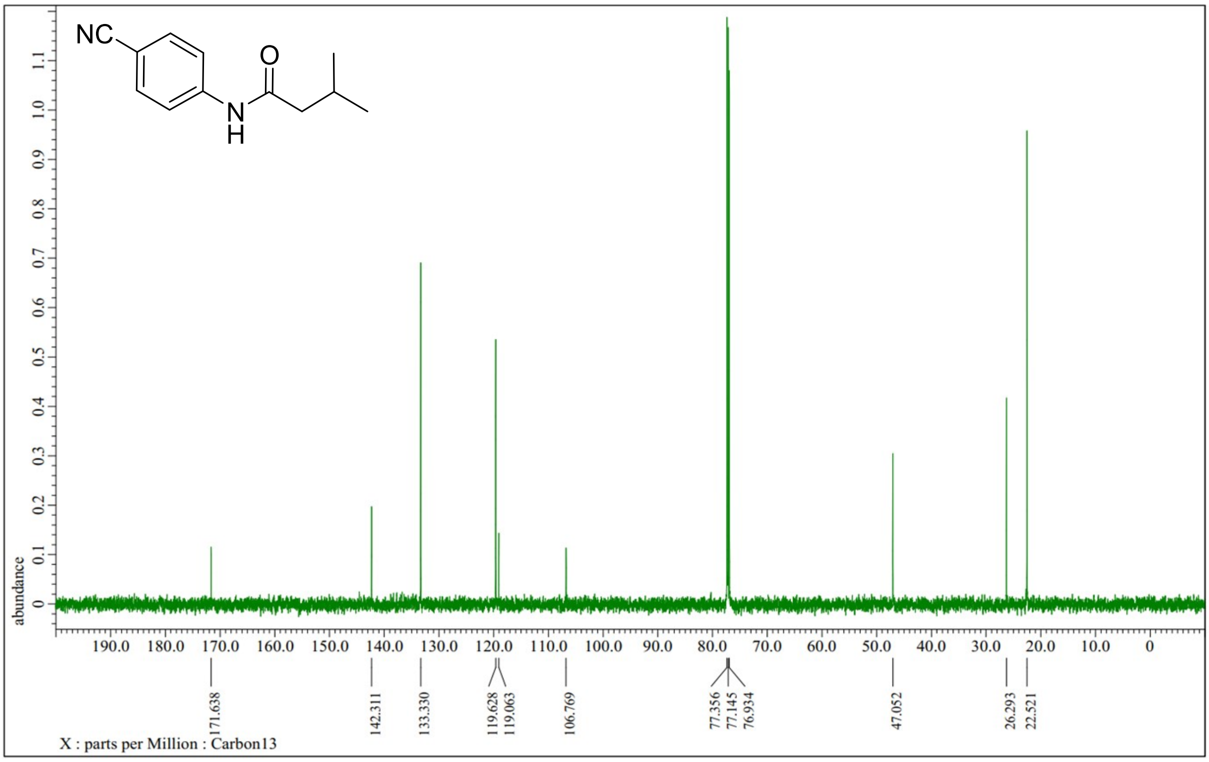

^{13}C NMR spectrum of *N*-(4-cyanophenyl)-3-methylbutanamide (3q)

***N*-(4-((methoxymethoxy)methyl)phenyl)butyramide (3r)**

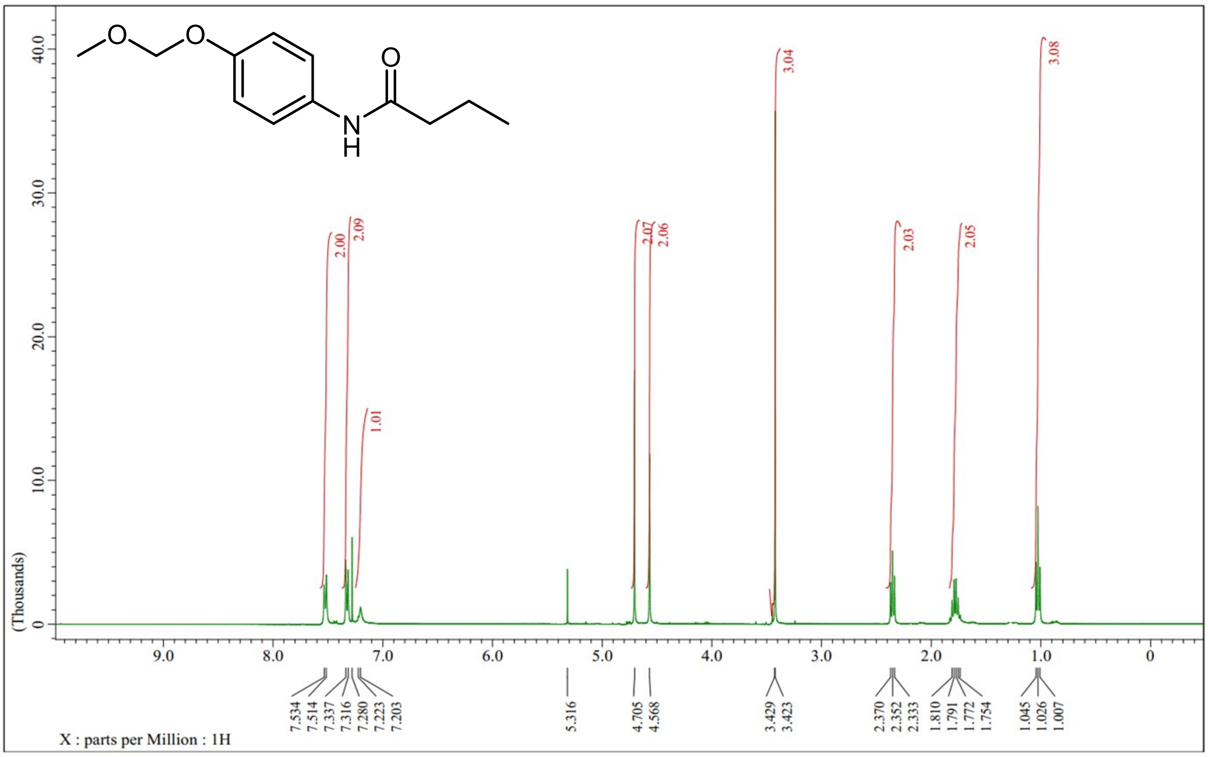

^{1}H NMR spectrum of *N*-(4-((methoxymethoxy)methyl)phenyl)butyramide **(3r)**

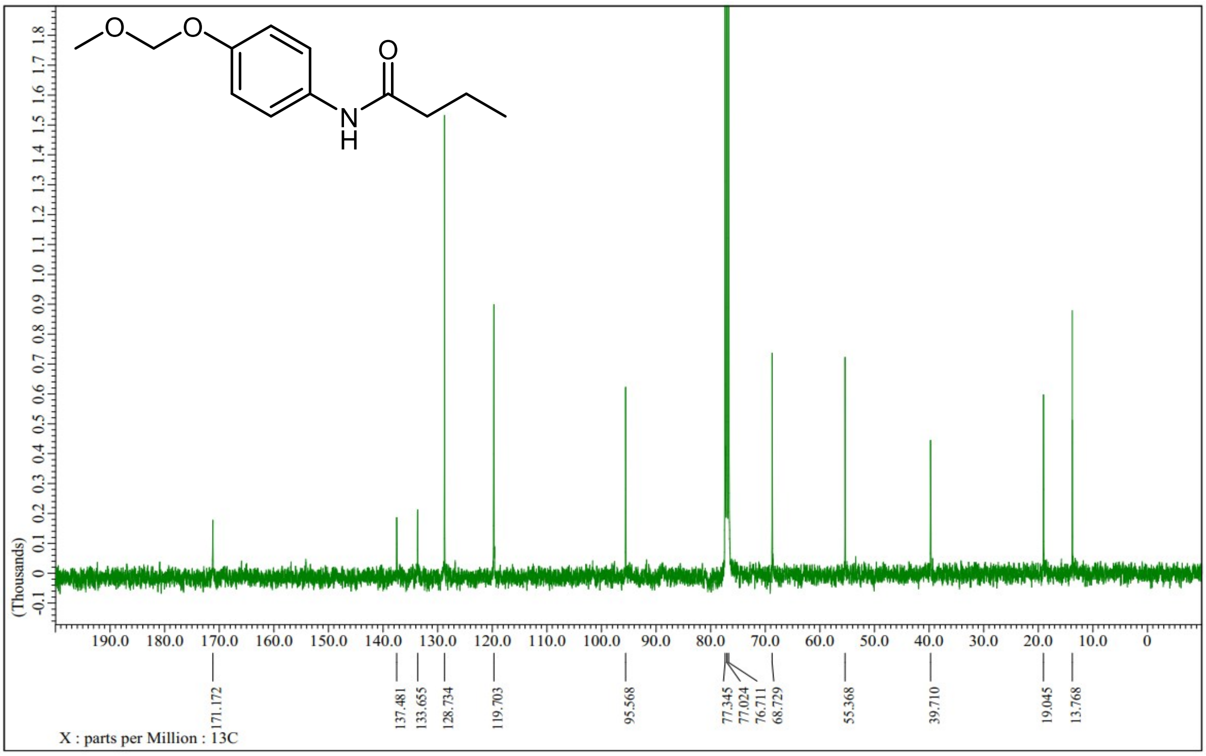

^{13}C NMR spectrum of *N*-(4-((methoxymethoxy)methyl)phenyl)butyramide **(3r)**

N-(4-butyramidophenyl)benzamide (3s)

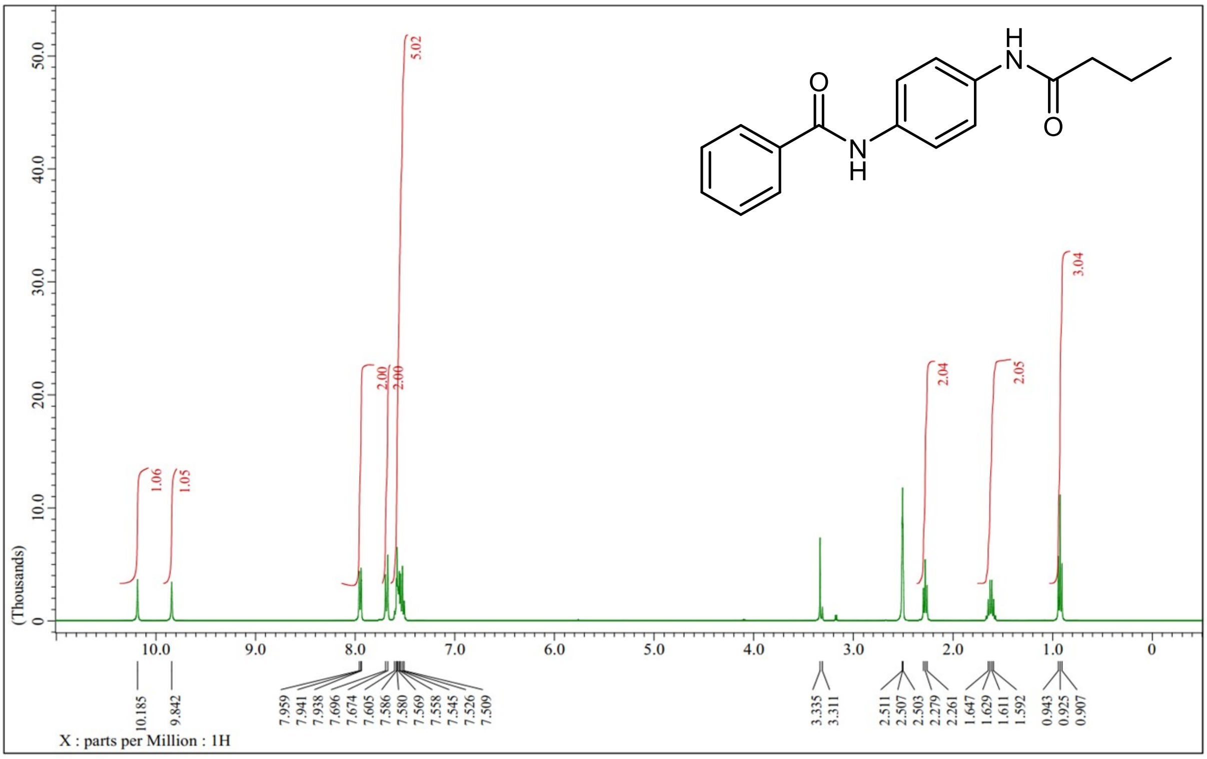

^{1}H NMR spectrum of *N*-(4-butyramidophenyl)benzamide **(3s)**

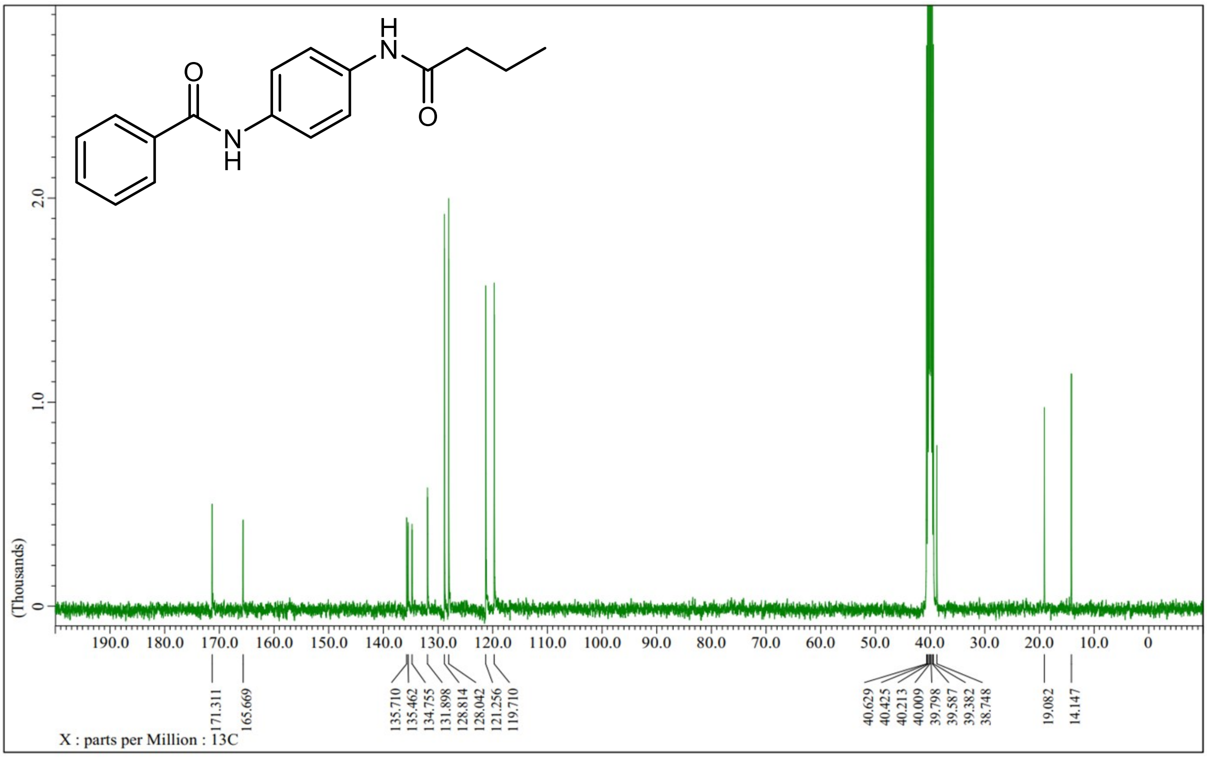

^{13}C NMR spectrum of *N*-(4-butyramidophenyl)benzamide **(3s)**

Methyl 4-benzamidobenzoate (3t)

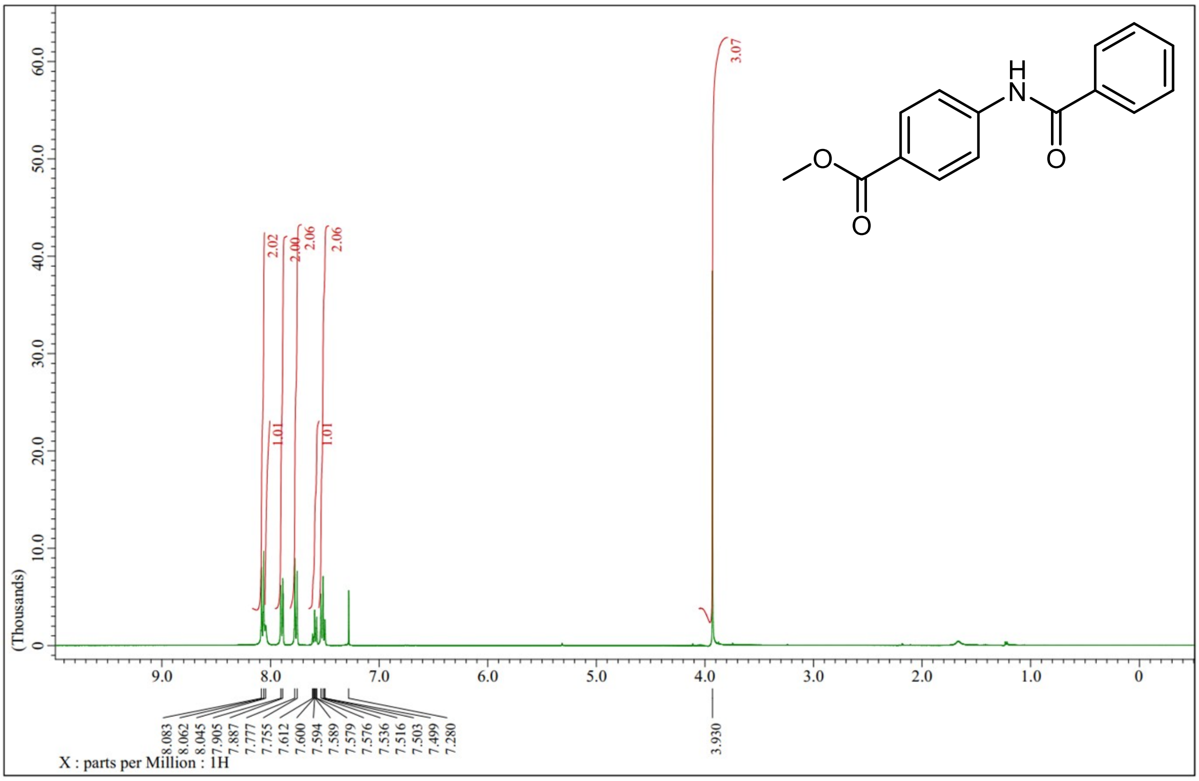

^{1}H NMR spectrum of methyl 4-benzamidobenzoate **(3t)**

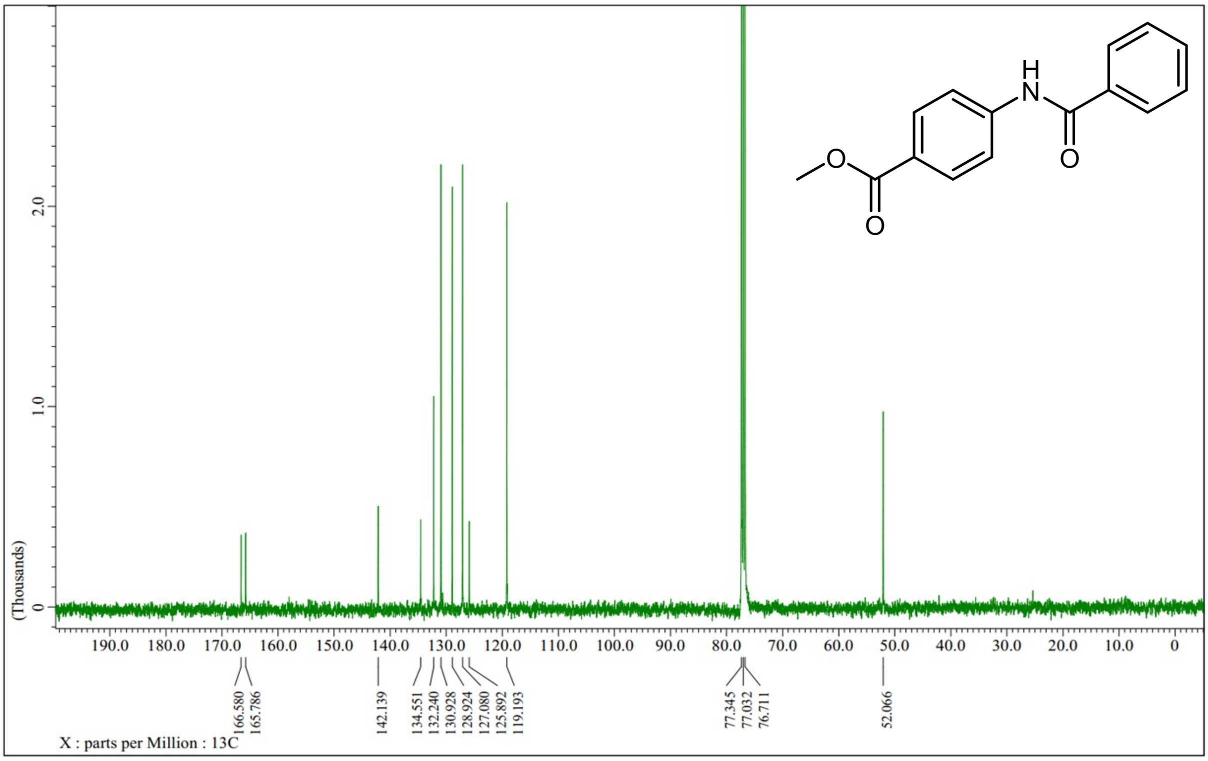

^{13}C NMR spectrum of methyl 4-benzamidobenzoate **(3)**

***N*-benzylbenzamide (3u)**

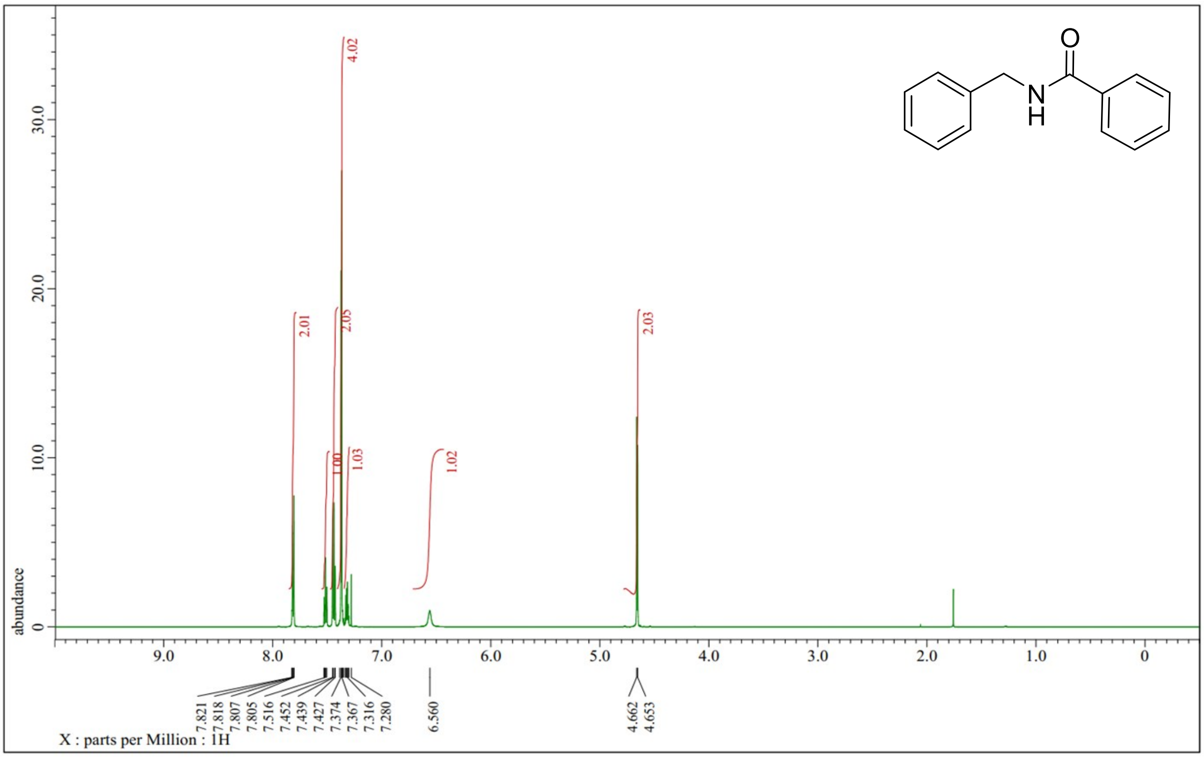

^{1}H NMR spectrum of *N*-benzylbenzamide (3u)

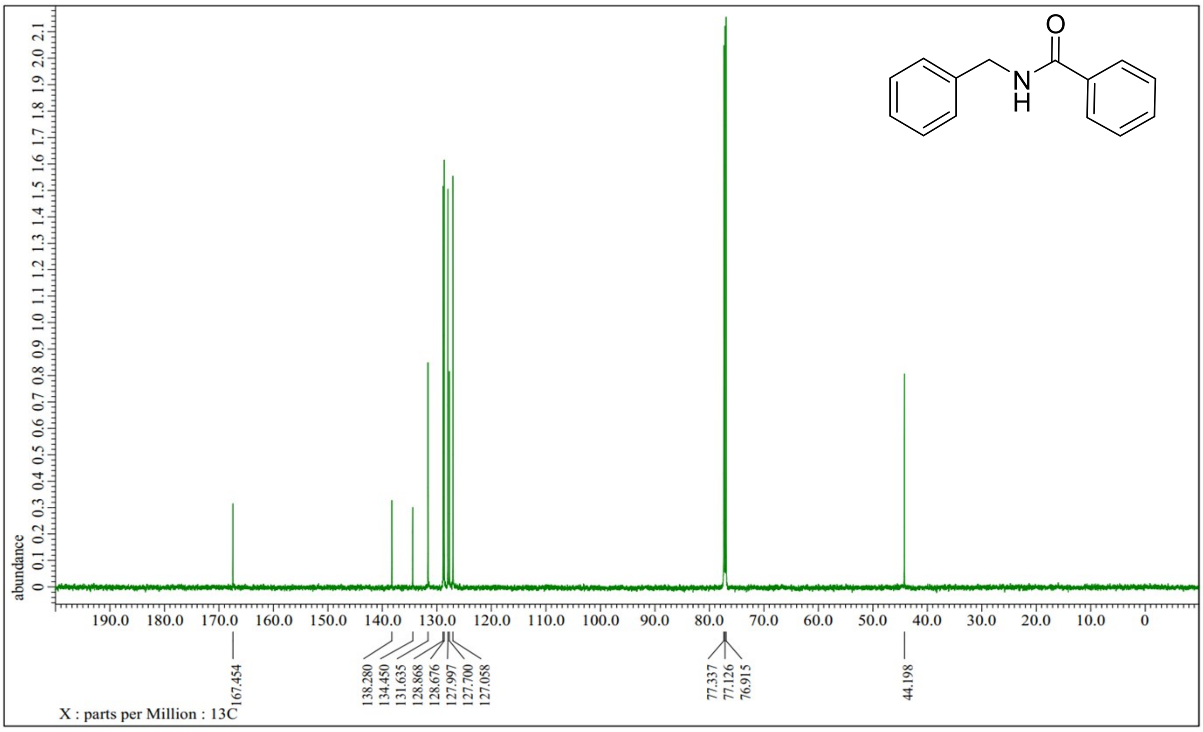

^{13}C NMR spectrum of *N*-benzylbenzamide (3u)

***N*-benzyl-3-methylbutanamide (3v)**

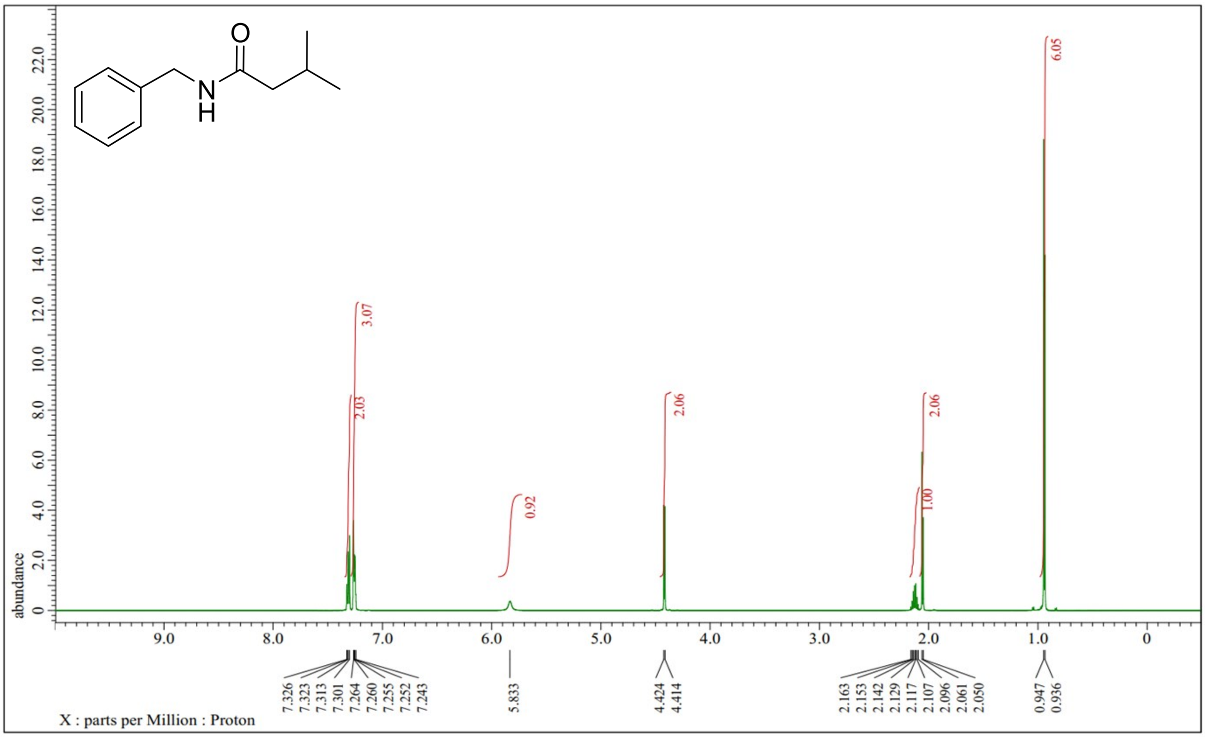

^{1}H NMR spectrum of *N*-benzyl-3-methylbutanamide (3v)

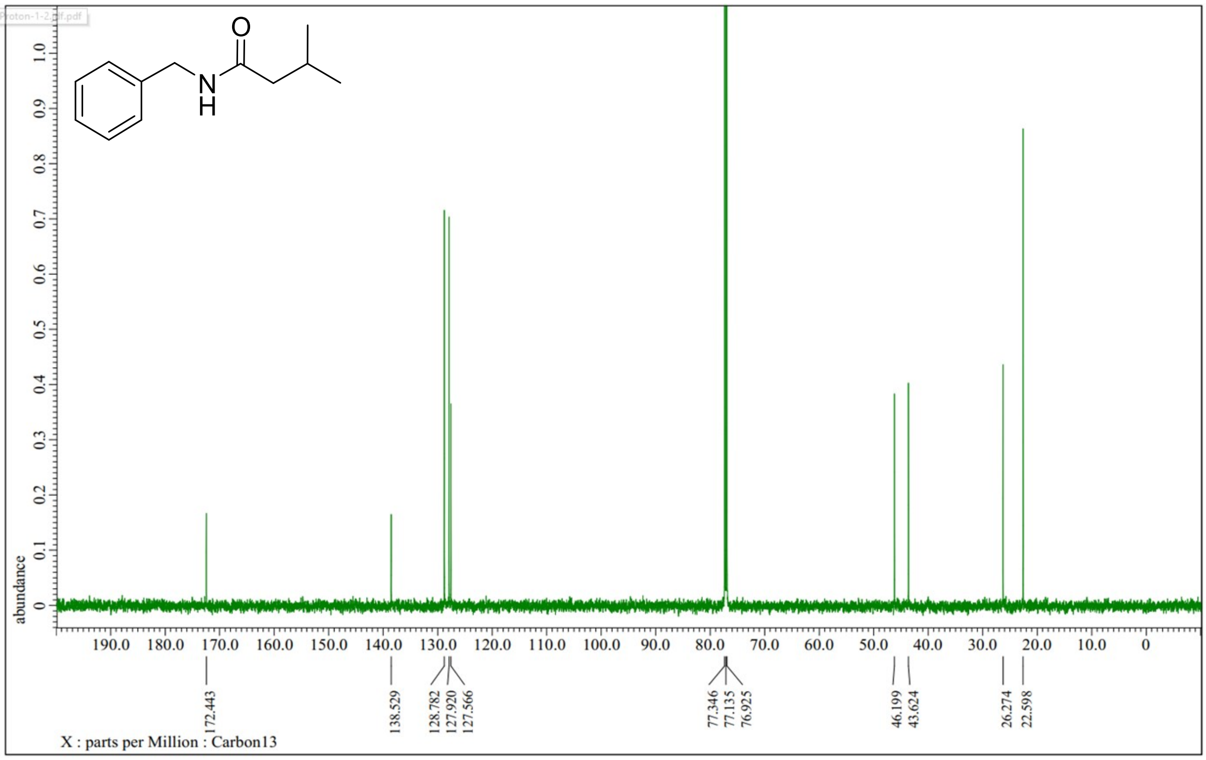

^{13}C NMR spectrum of *N*-benzyl-3-methylbutanamide (3v)

N-benzyl-1-naphthamide (3w)

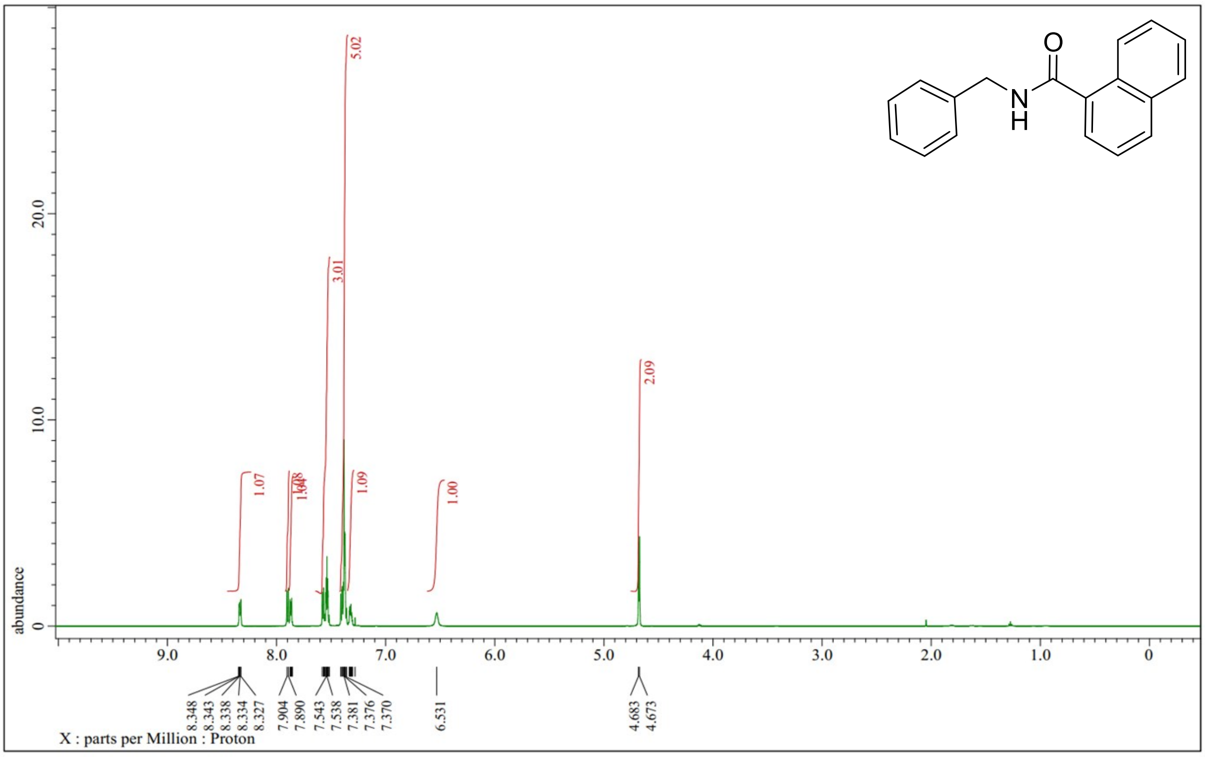

^{1}H NMR spectrum of N-benzyl-1-naphthamide (3w)

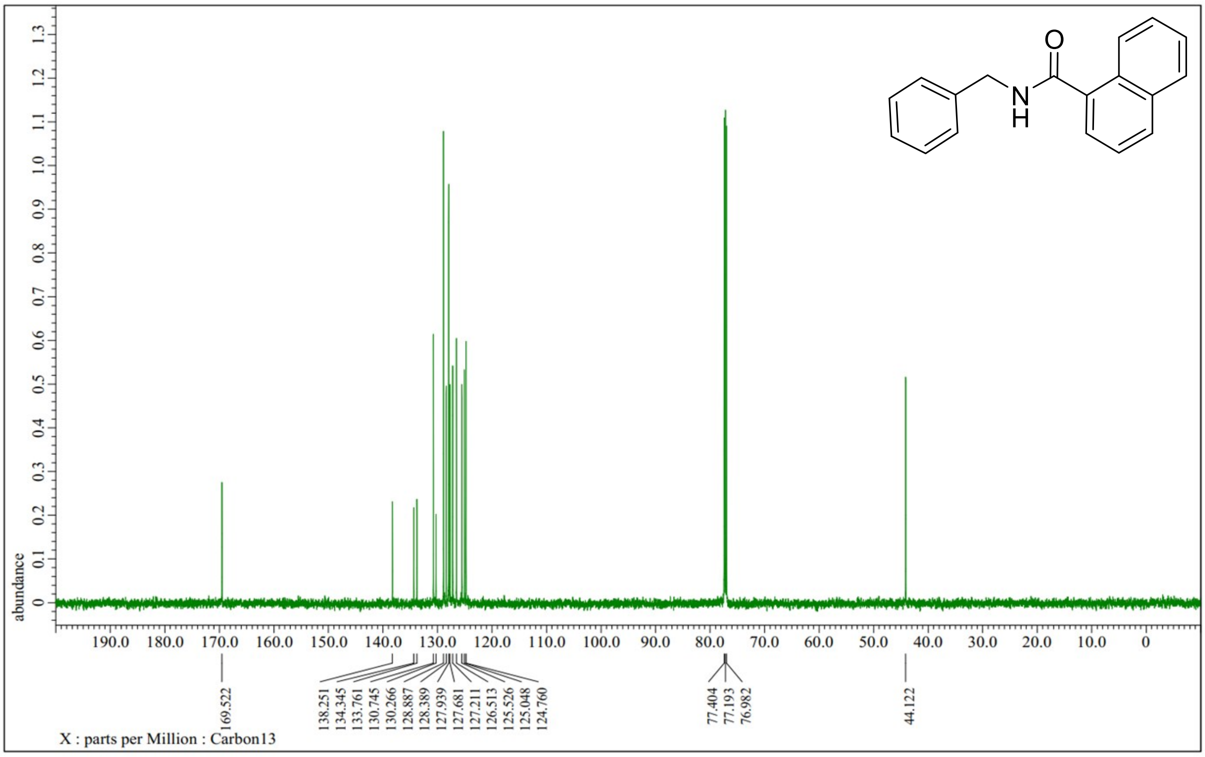

^{13}C NMR spectrum of N-benzyl-1-naphthamide (3w)

***N*-butylbenzamide (3x)**

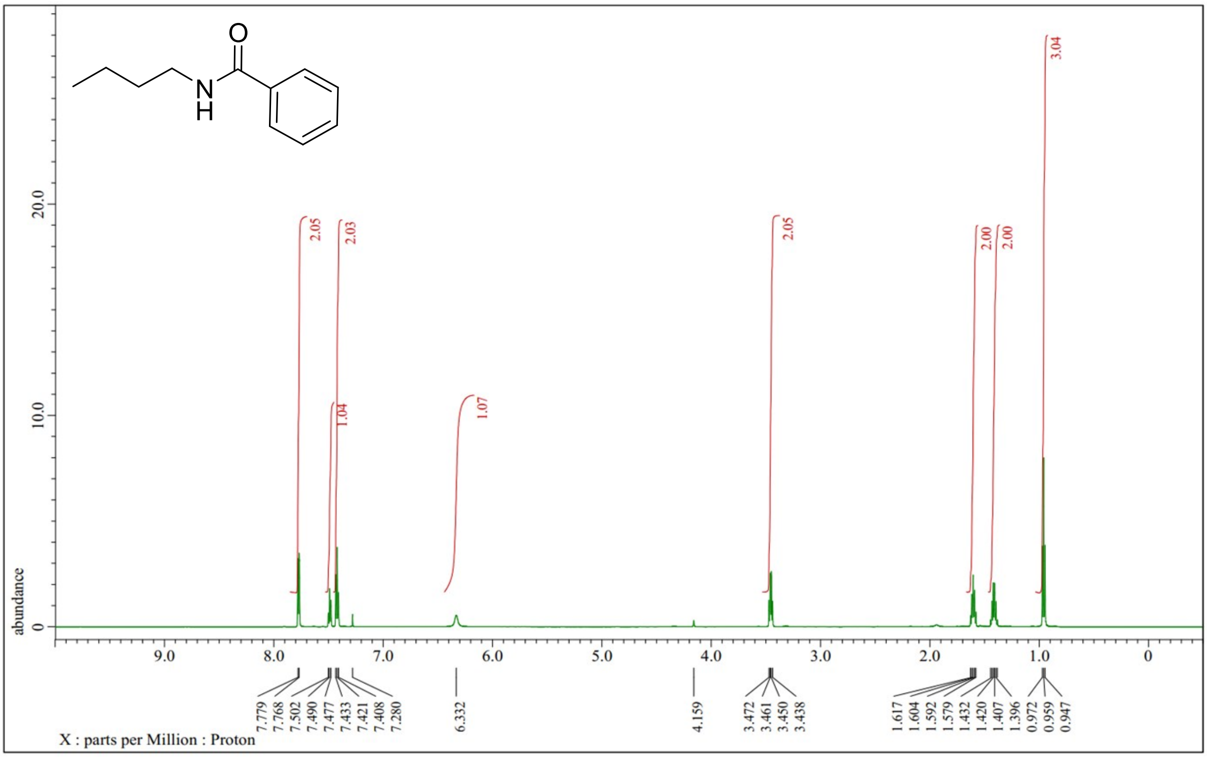

^{1}H NMR spectrum of *N*-butylbenzamide (3x)

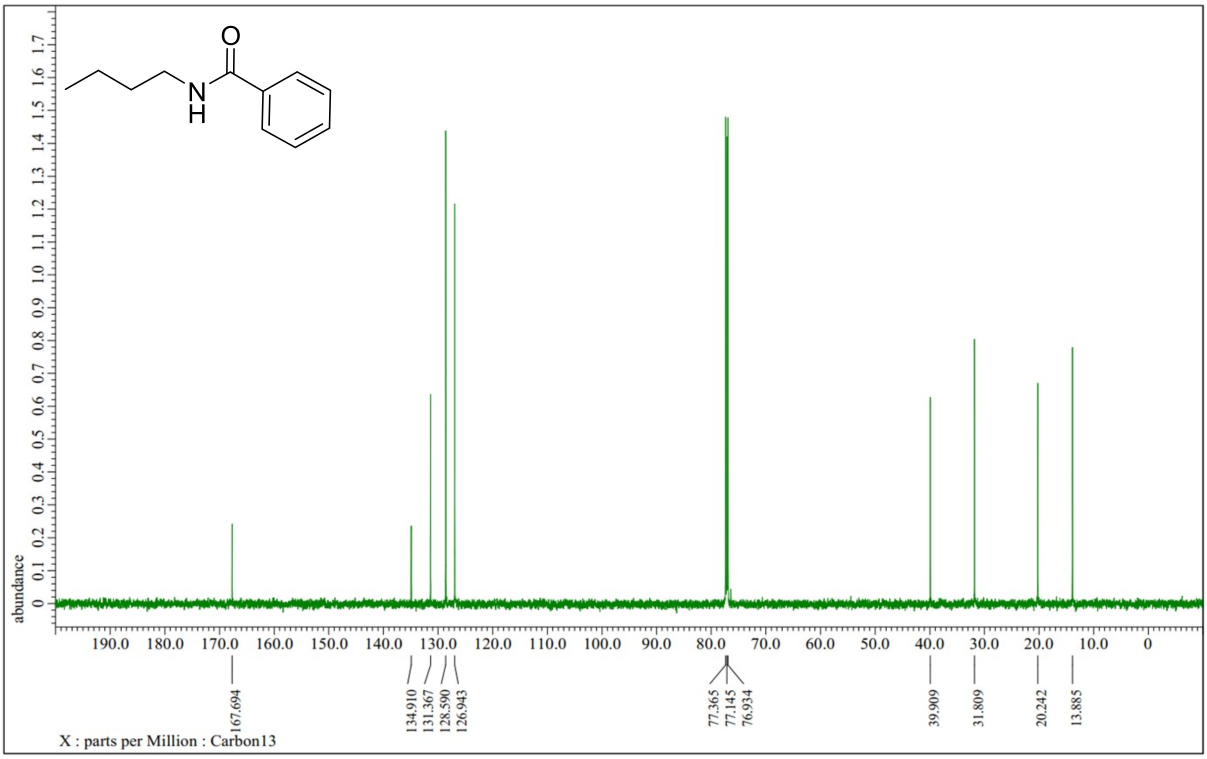

^{13}C NMR spectrum of *N*-butylbenzamide (3x)

***N*-butylcyclohexanecarboxamide (3y)**

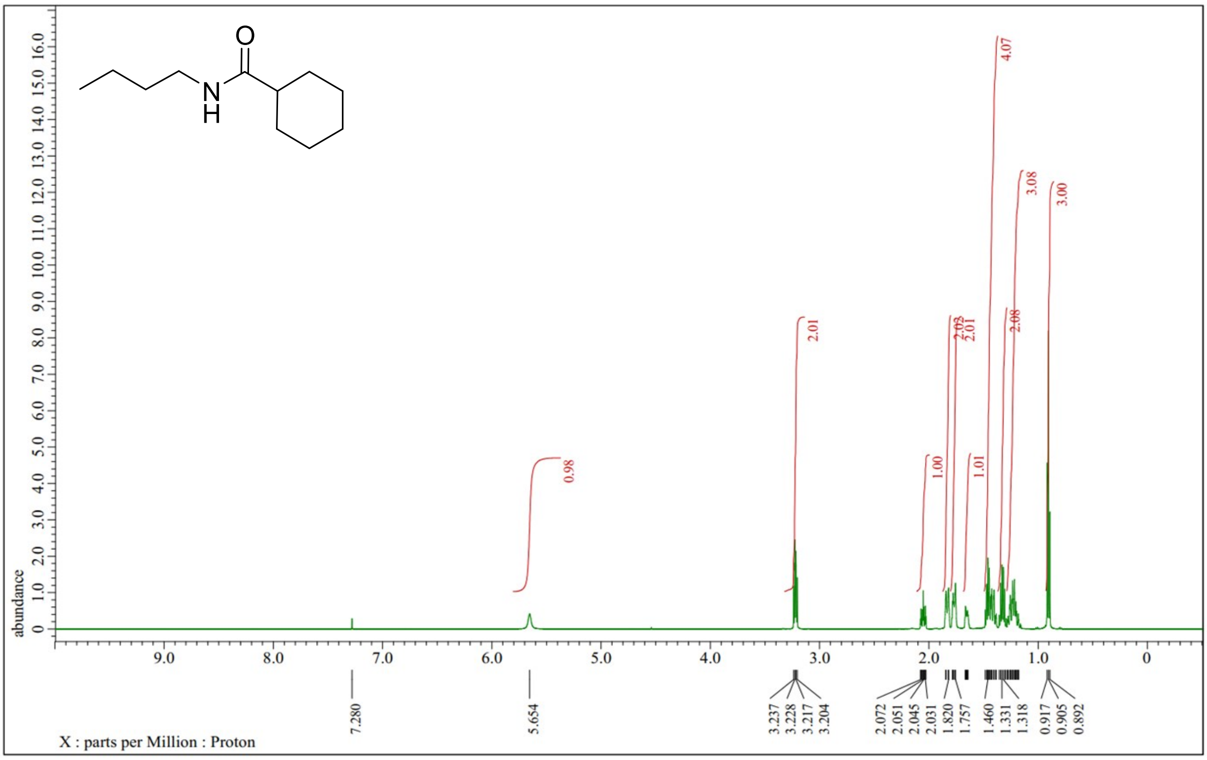

^{1}H NMR spectrum of *N*-butylcyclohexanecarboxamide (3y)

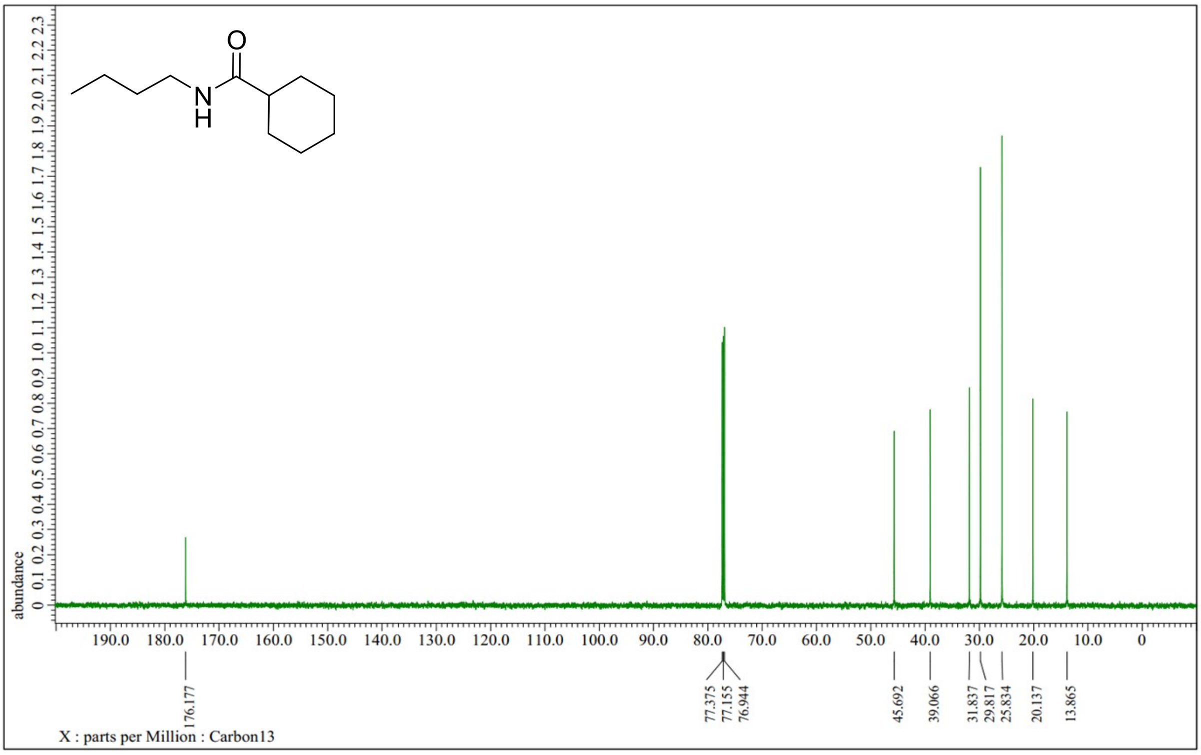

^{13}C NMR spectrum of *N*-butylcyclohexanecarboxamide (3y)

N-isobutylbenzamide (3z)

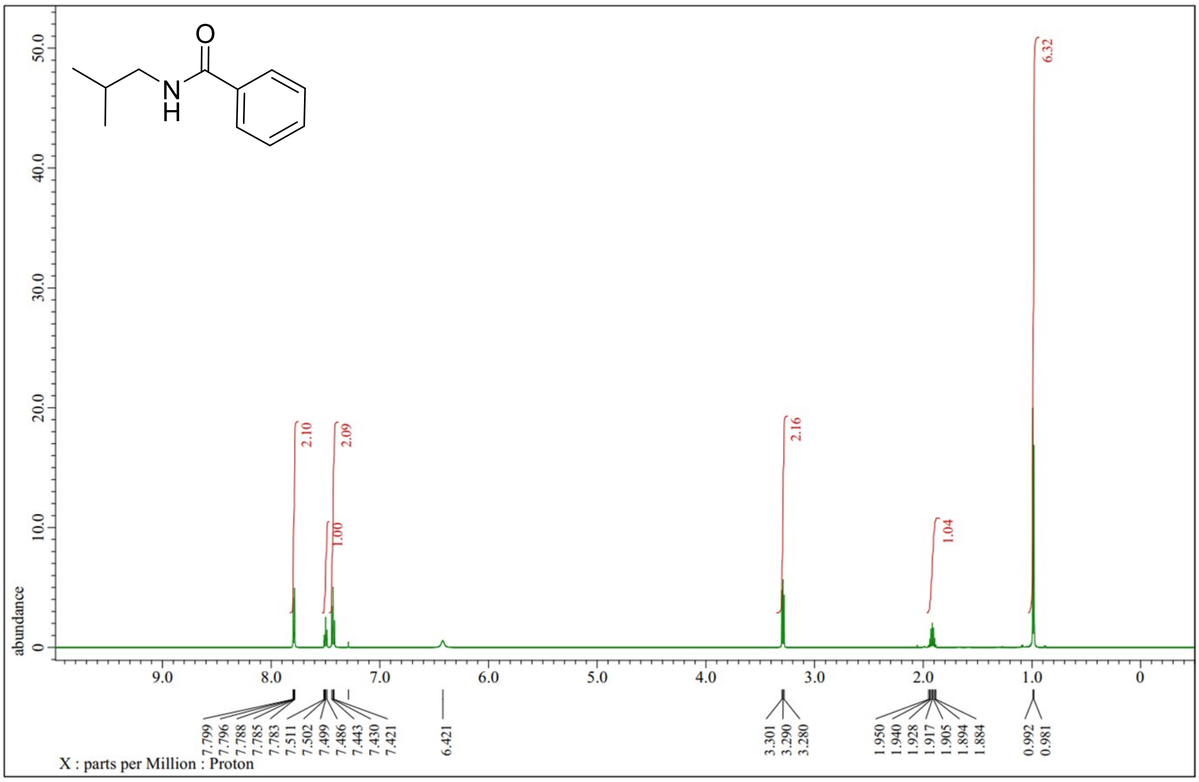

^{1}H NMR spectrum of *N*-isobutylbenzamide (3z)

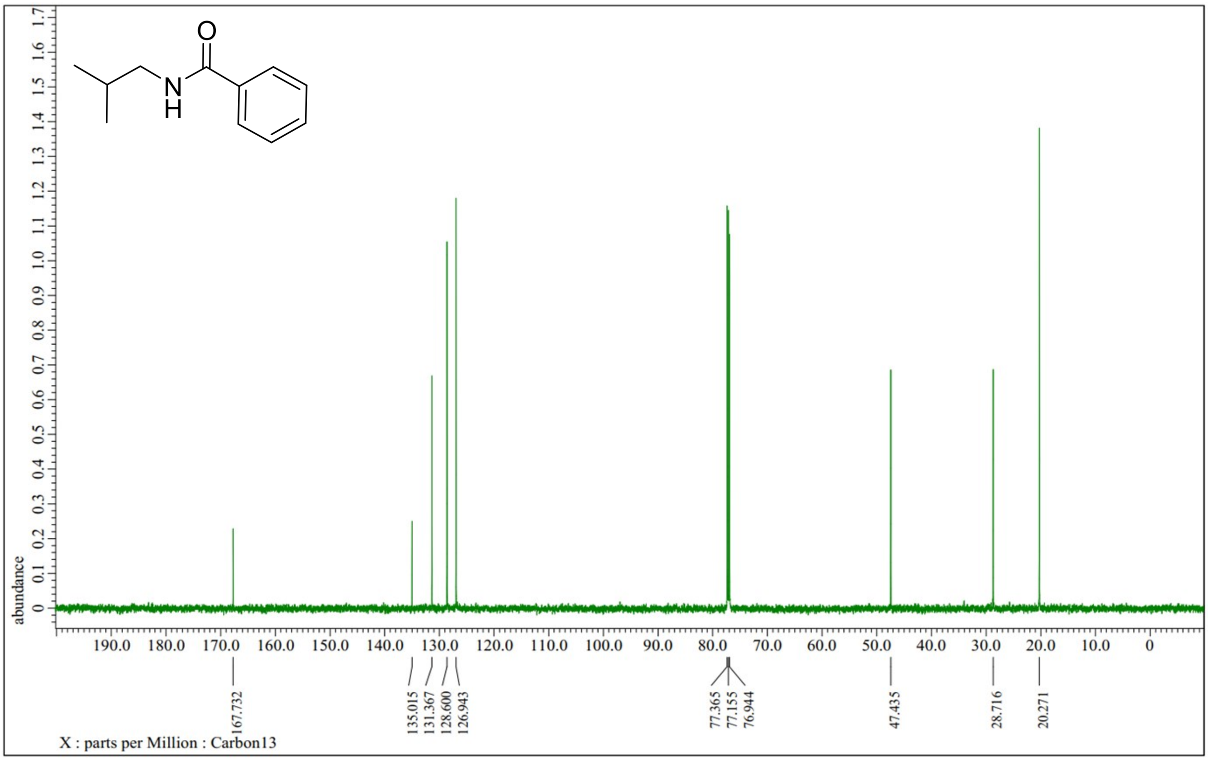

^{13}C NMR spectrum of *N*-isobutylbenzamide (3z)

***N*-isobutyl-3-phenylpropanamide (3aa)**

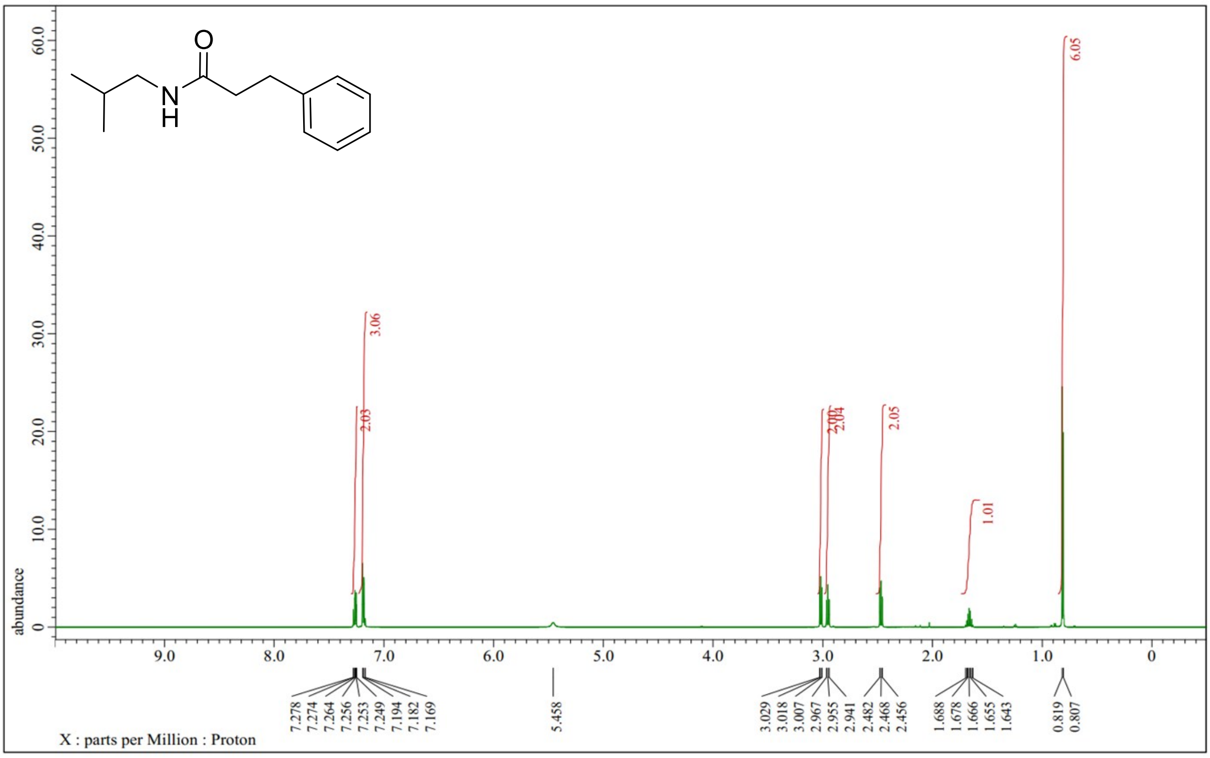

^{1}H NMR spectrum of *N*-isobutyl-3-phenylpropanamide (3aa)

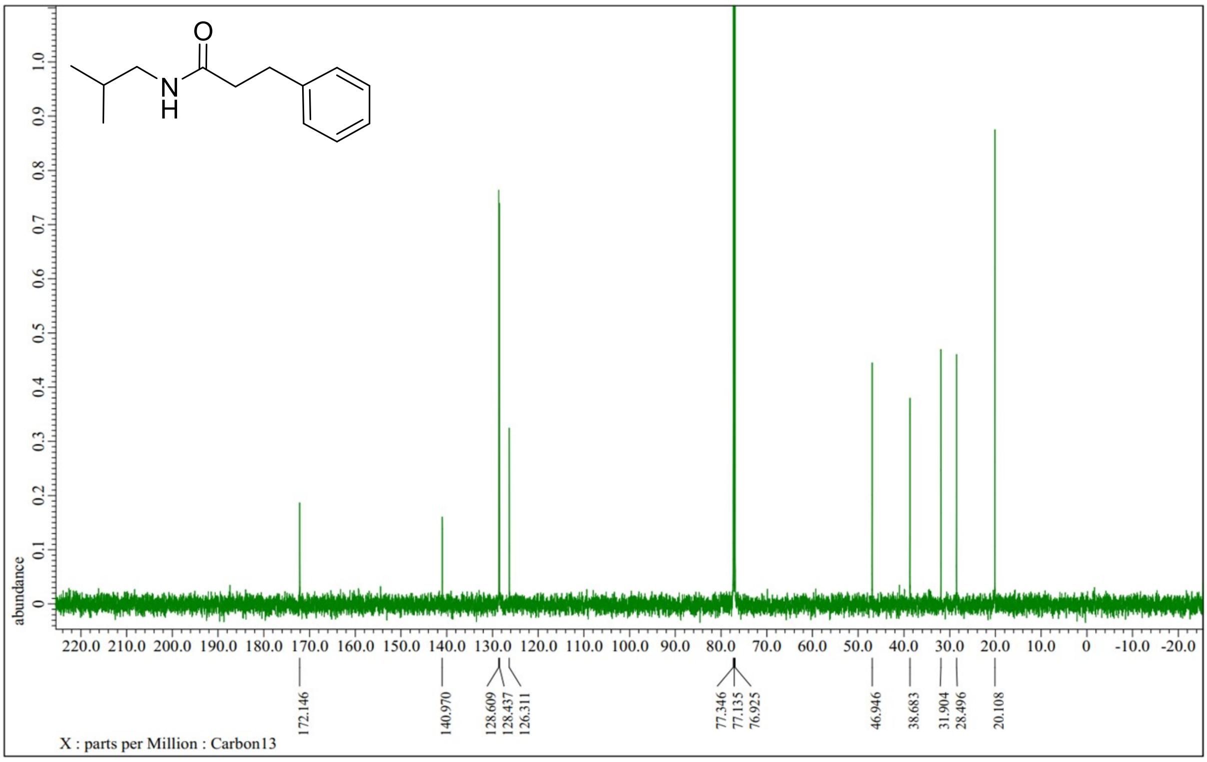

^{13}C NMR spectrum of *N*-isobutyl-3-phenylpropanamide (3aa)

***N*-cyclohexylbenzamide (3ab)**

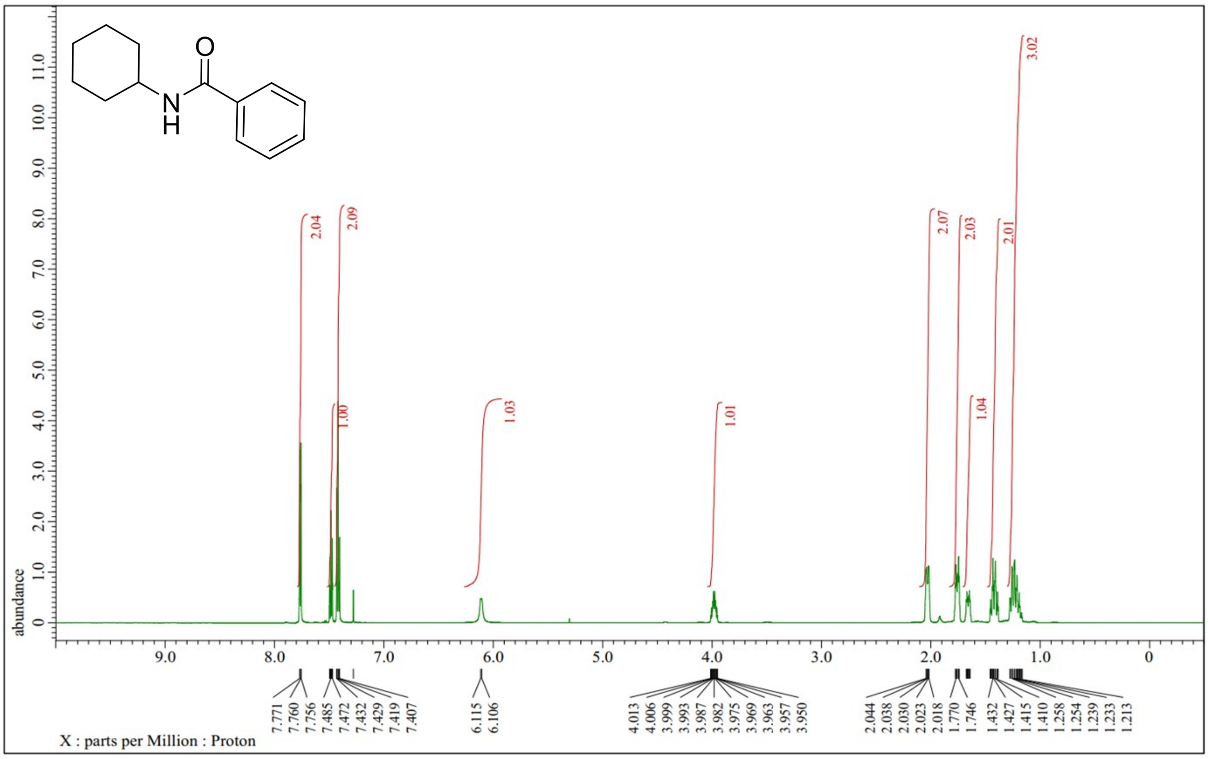

^{1}H NMR spectrum of *N*-cyclohexylbenzamide (3ab)

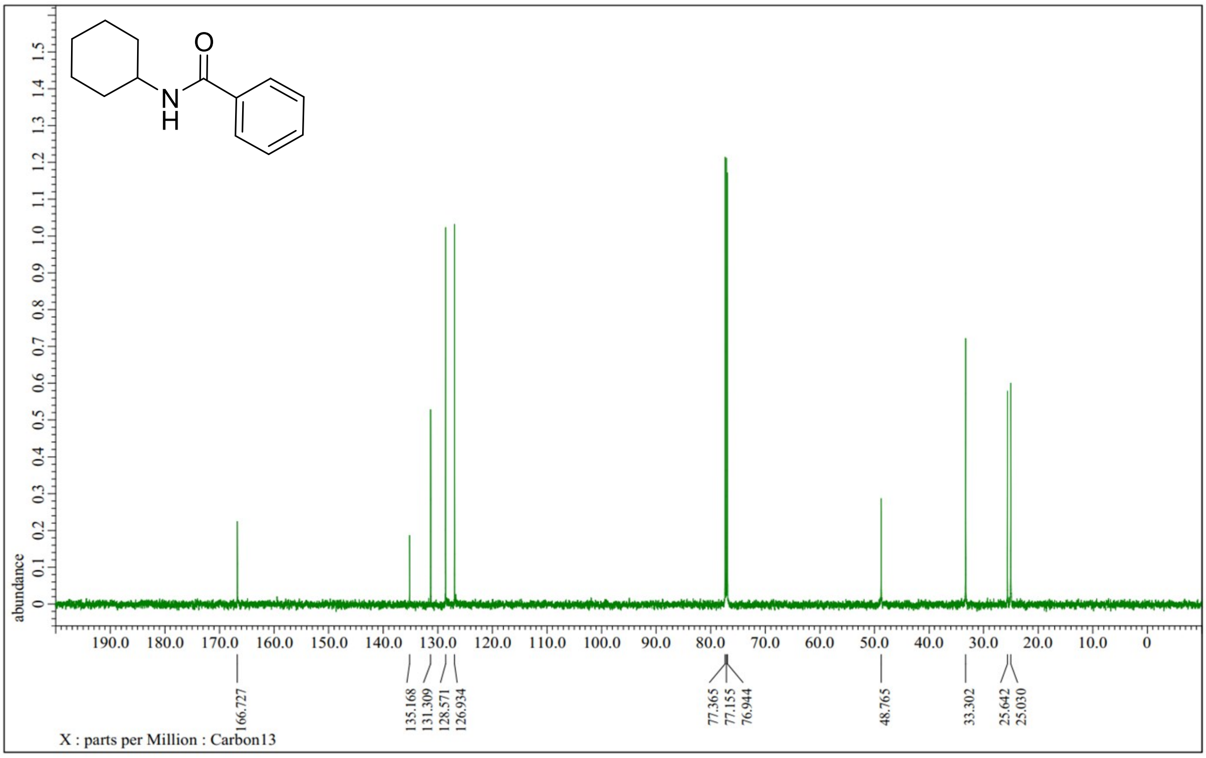

^{13}C NMR spectrum of *N*-cyclohexylbenzamide (3ab)

N-cyclohexyl-1-naphthamide (3ac)

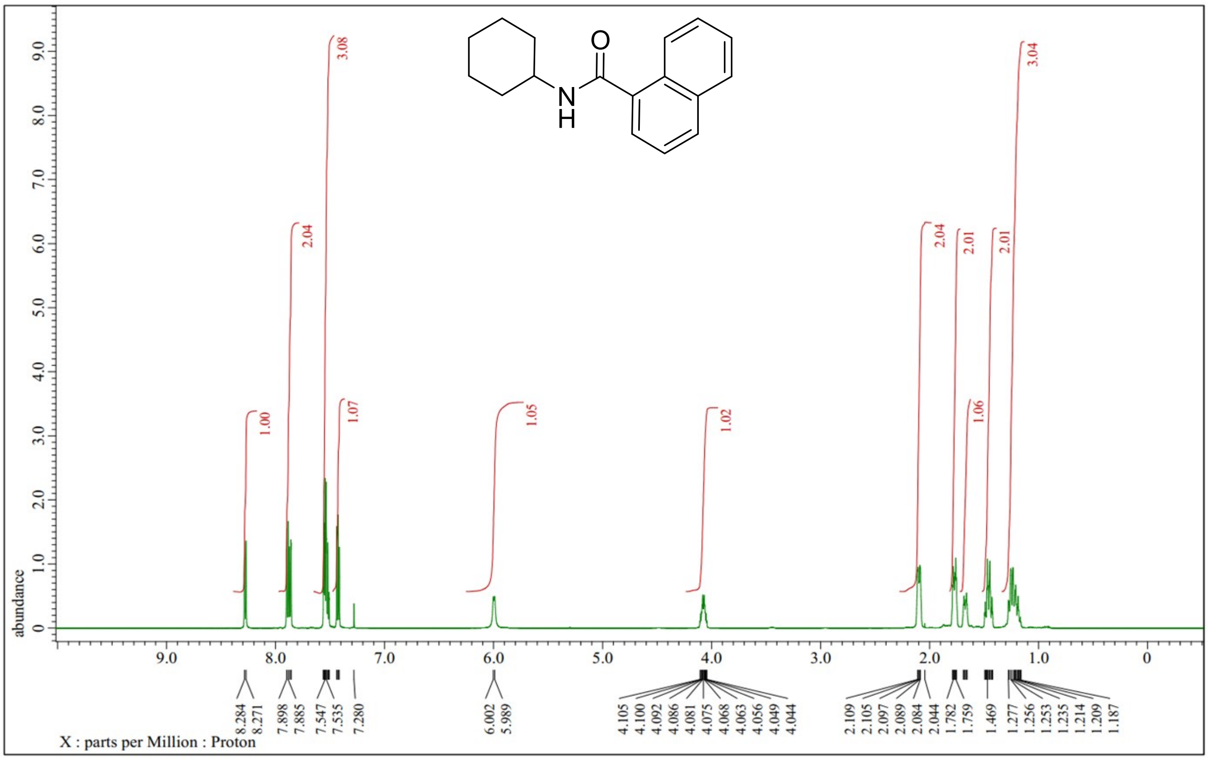

^{1}H NMR spectrum of *N*-cyclohexyl-1-naphthamide (3ac)

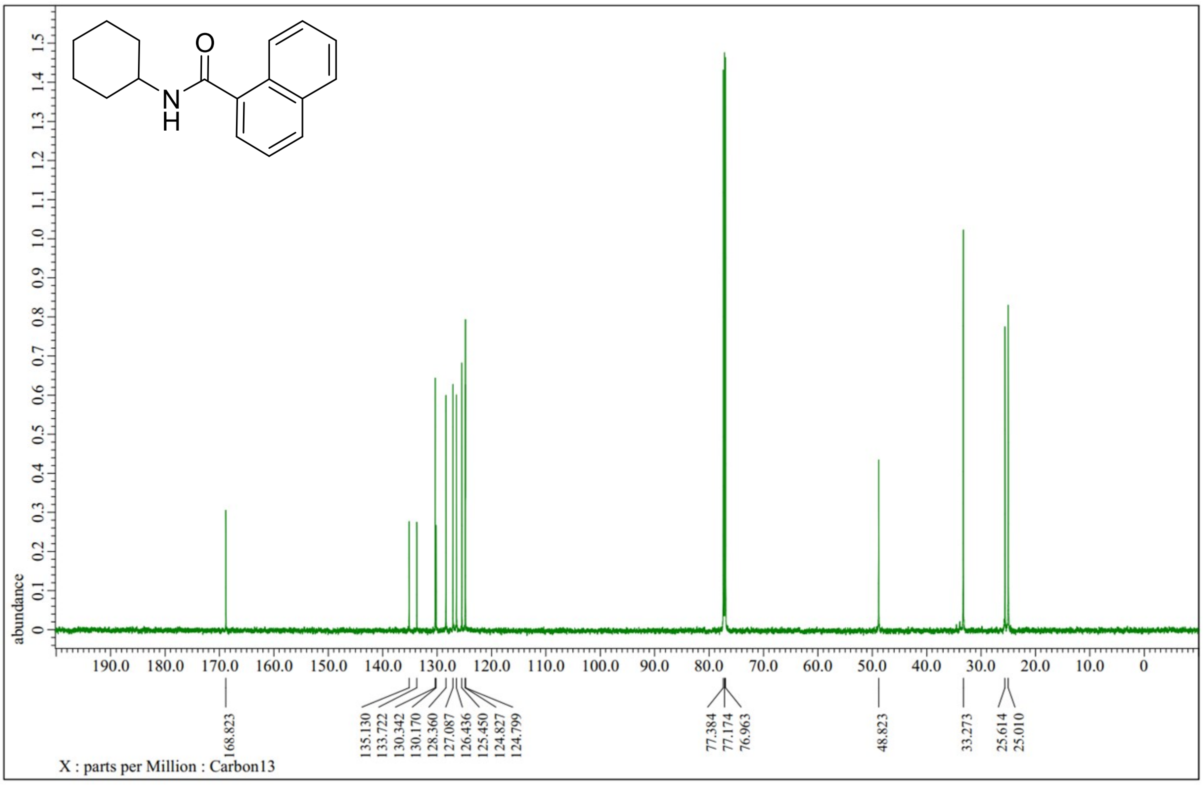

^{13}C NMR spectrum of *N*-cyclohexyl-1-naphthamide (3ac)

Phenyl(piperidin-1-yl)methanone (3ad)

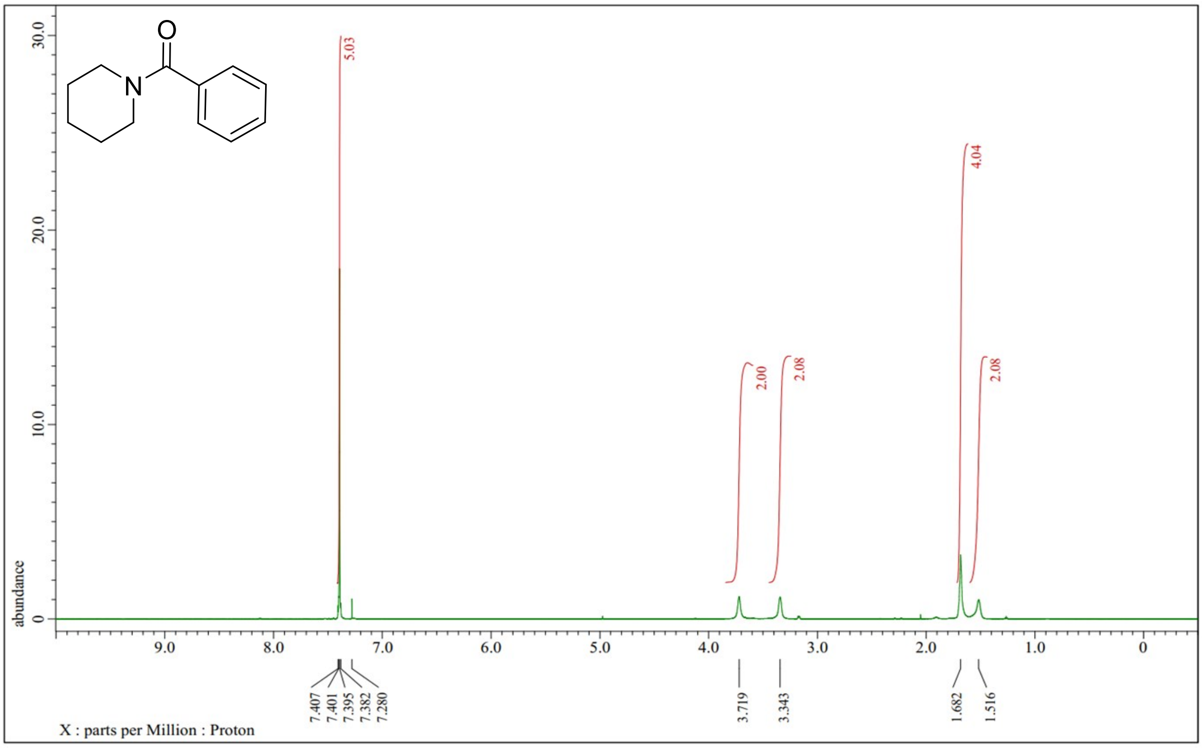

^{1}H NMR spectrum of phenyl(piperidin-1-yl)methanone (3ad)

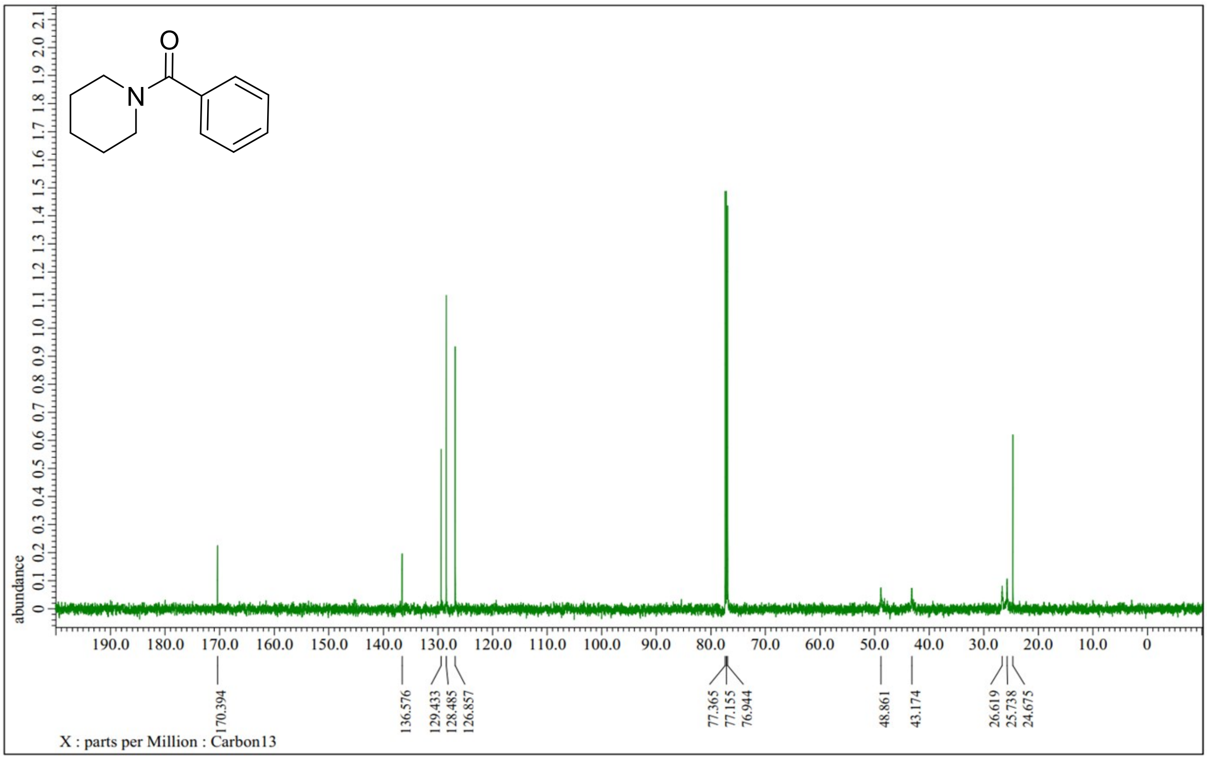

^{13}C NMR spectrum of phenyl(piperidin-1-yl)methanone (3ad)

3-phenyl-1-(piperidin-1-yl)propan-1-one (3ae)

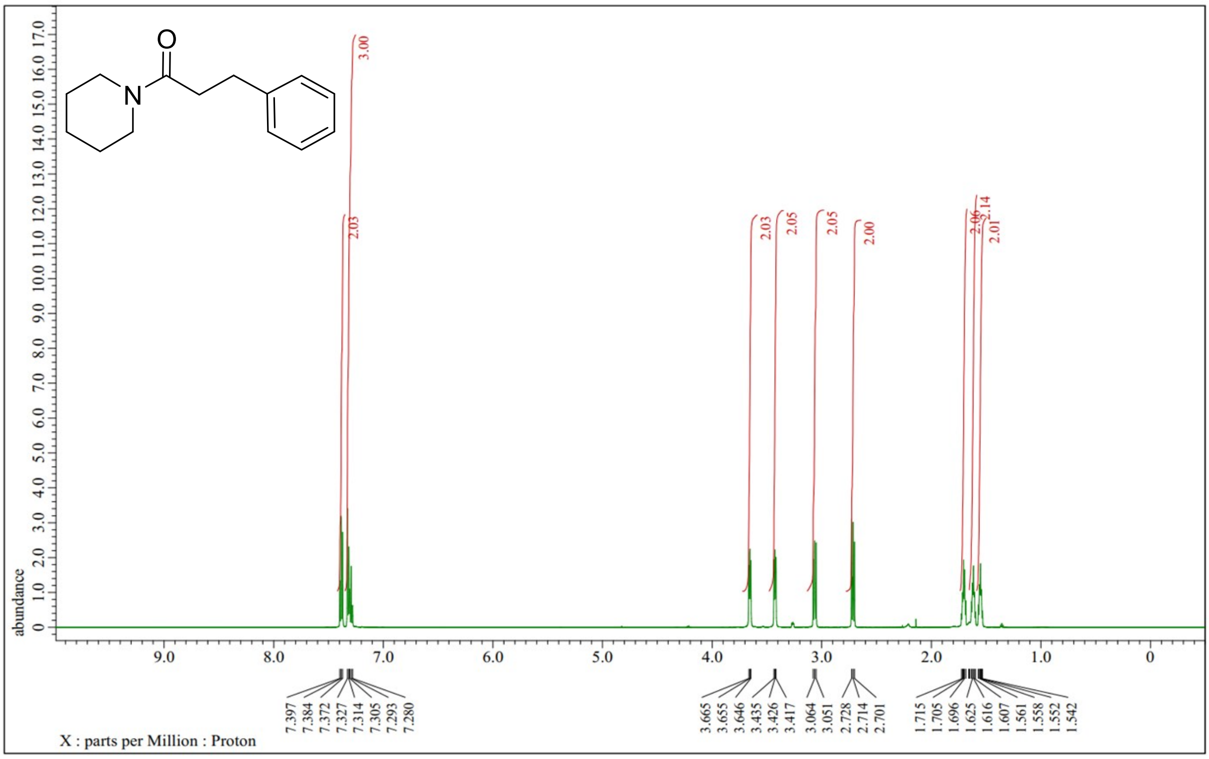

^{1}H NMR spectrum of 3-phenyl-1-(piperidin-1-yl)propan-1-one (3ae)

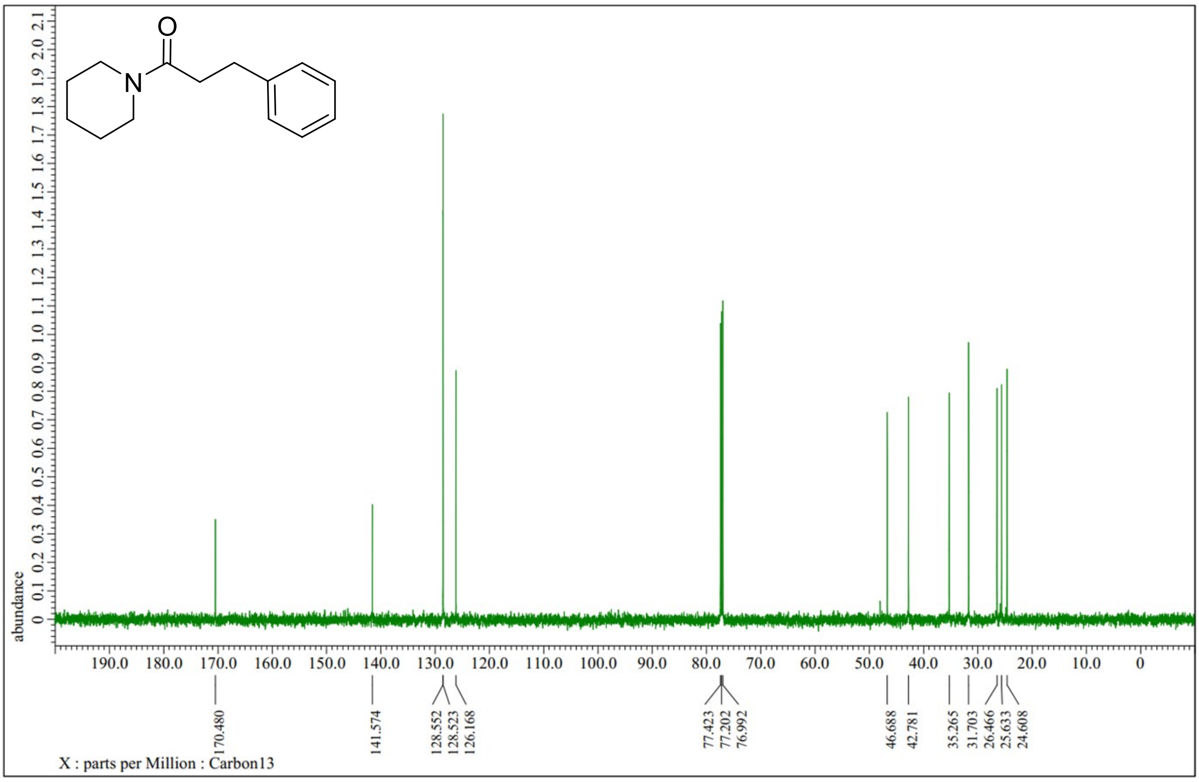

^{13}C NMR spectrum of 3-phenyl-1-(piperidin-1-yl)propan-1-one (3ae)

***N*-allylbenzamide (3af)**

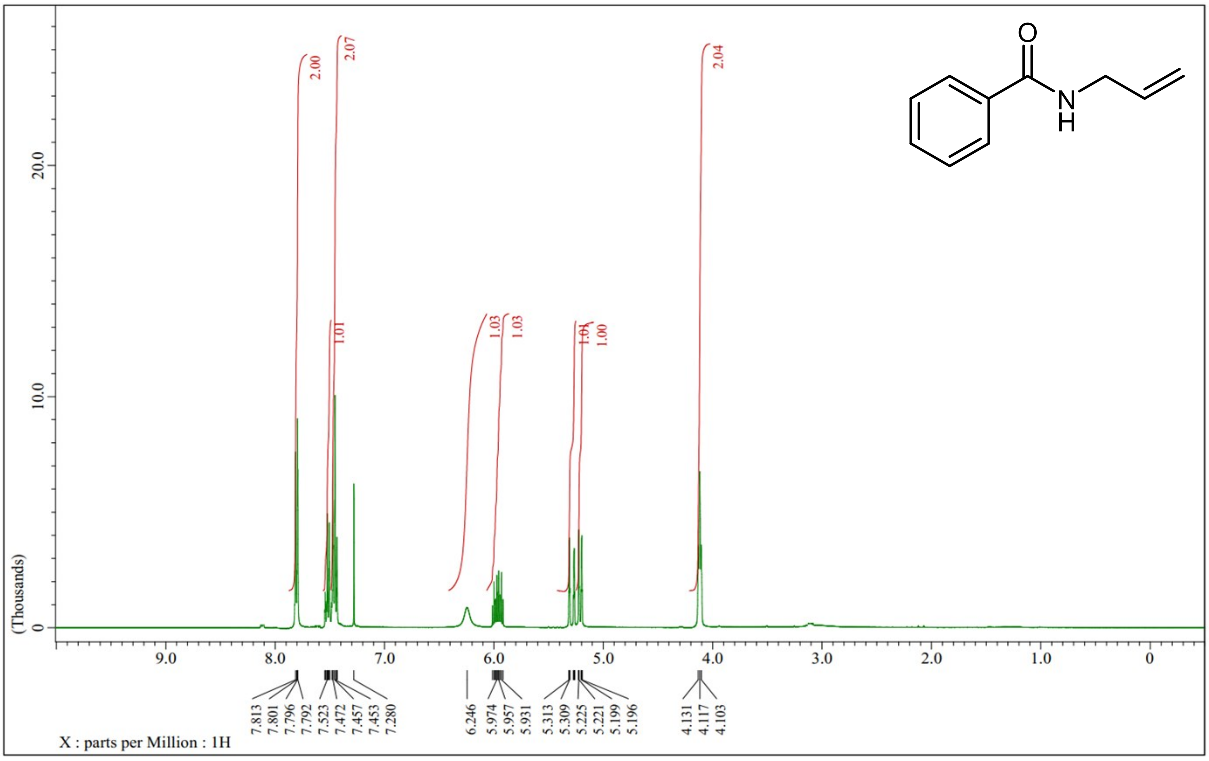

^{1}H NMR spectrum of *N*-allylbenzamide **(3af)**

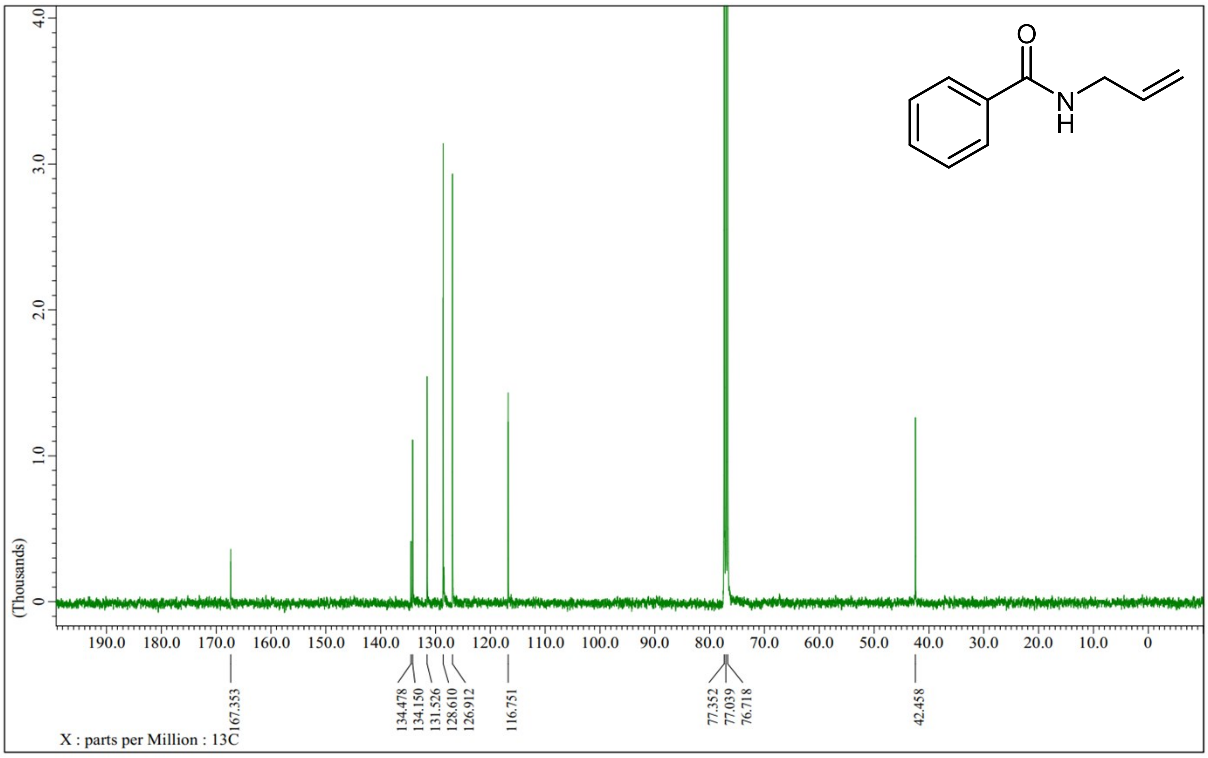

^{13}C NMR spectrum of *N*-allylbenzamide **(3af)**

***N*-(prop-2-yn-1-yl)benzamide (3ag)**

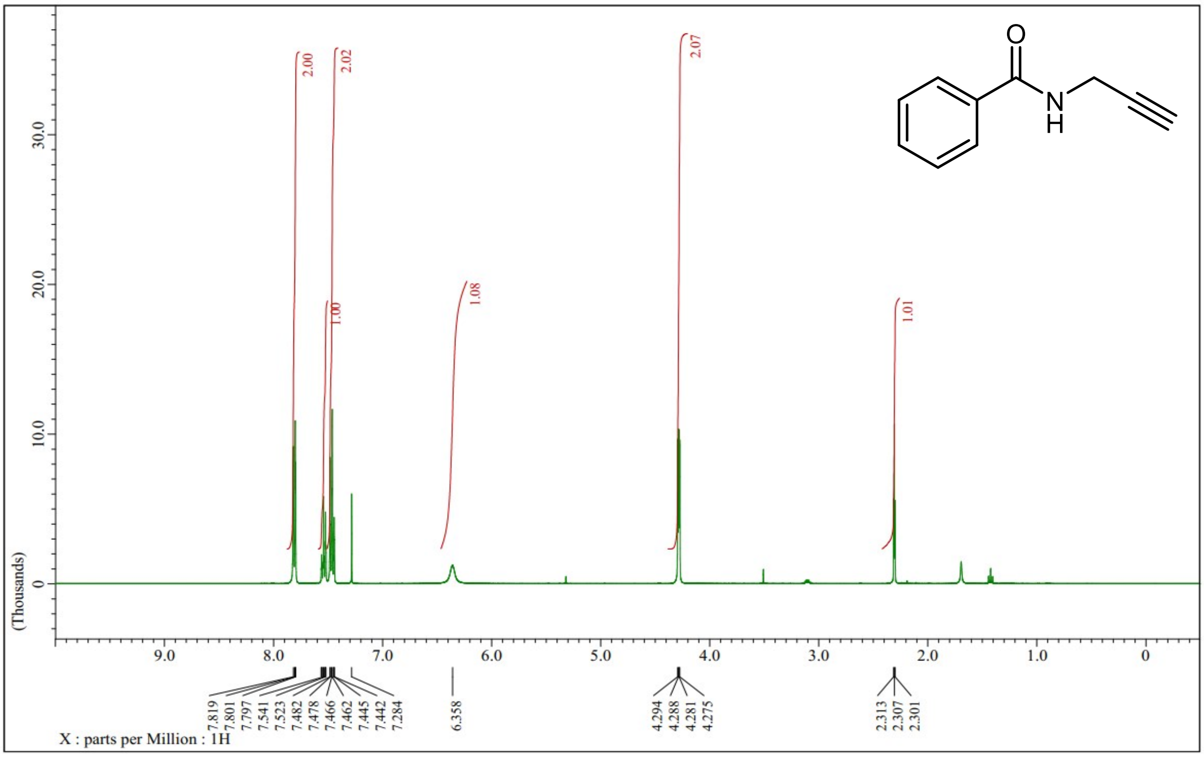

^{1}H NMR spectrum of *N*-(prop-2-yn-1-yl)benzamide (3ag)

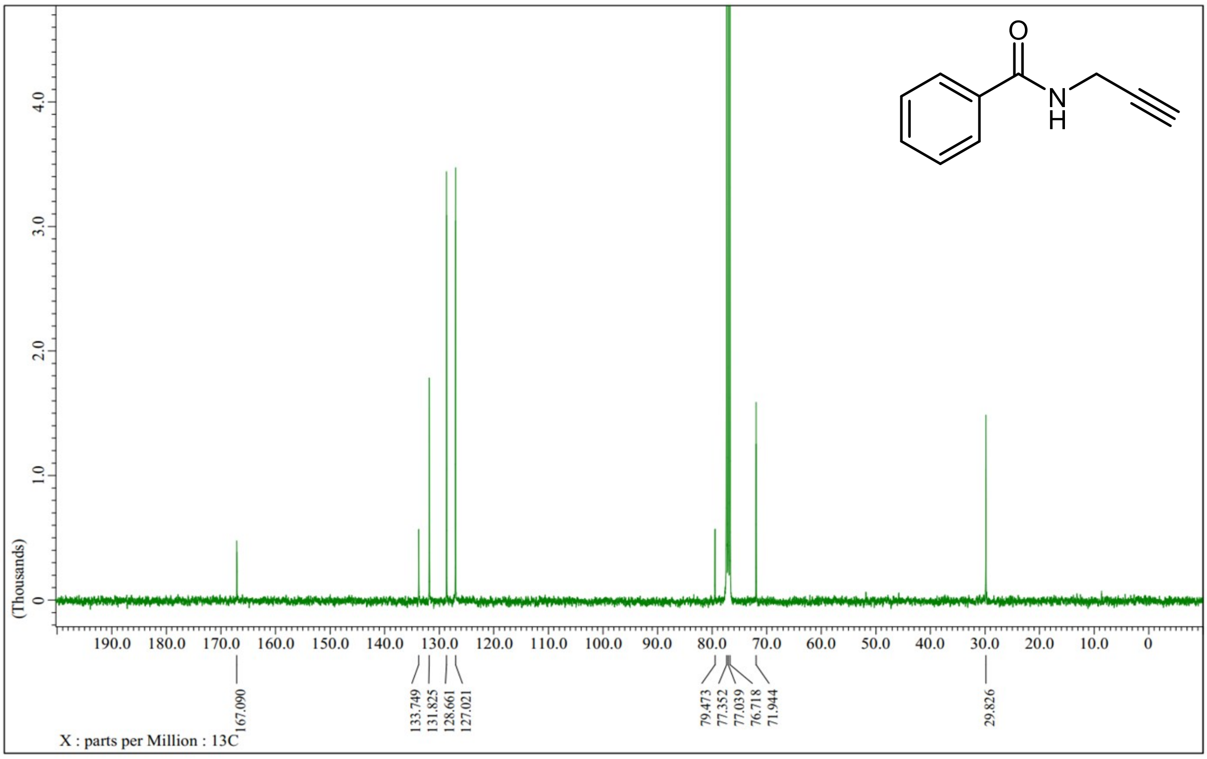

^{13}C NMR spectrum of *N*-(prop-2-yn-1-yl)benzamide (3ag)

2-methyl-*N*-phenylbenzamide (3ah)

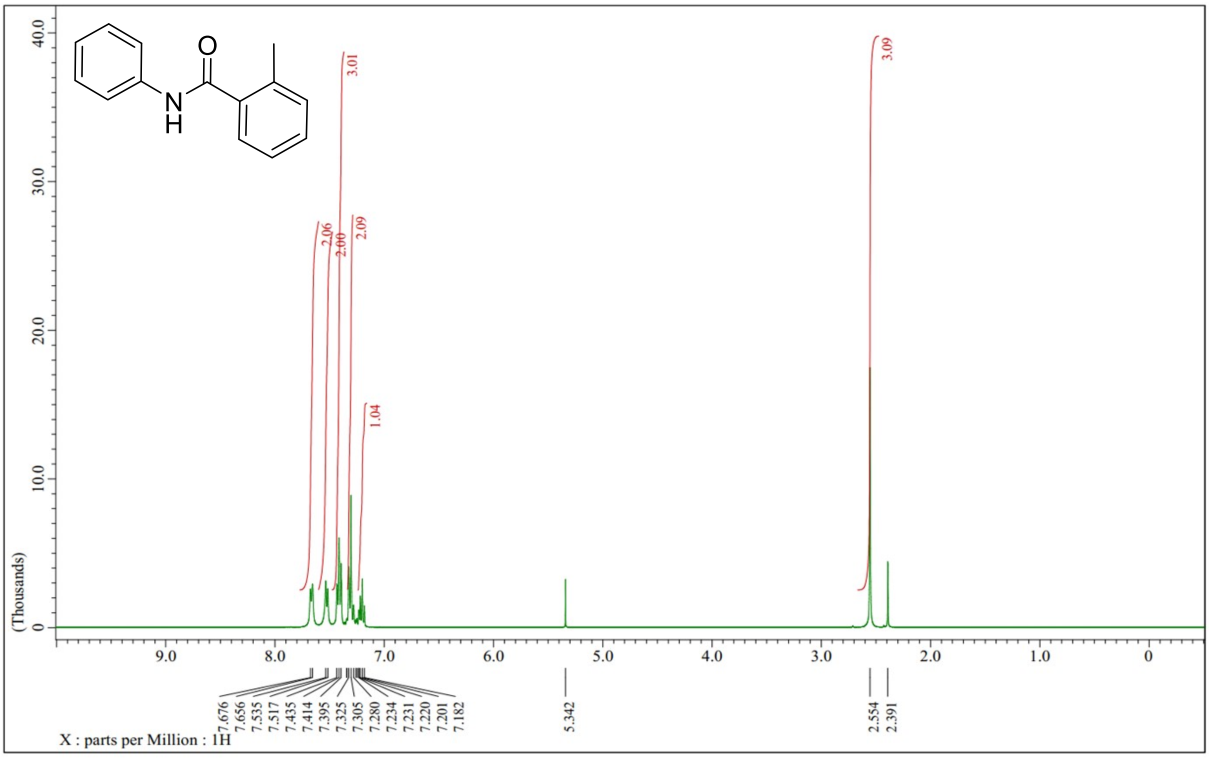

^{1}H NMR spectrum of 2-methyl-*N*-phenylbenzamide (3ah)

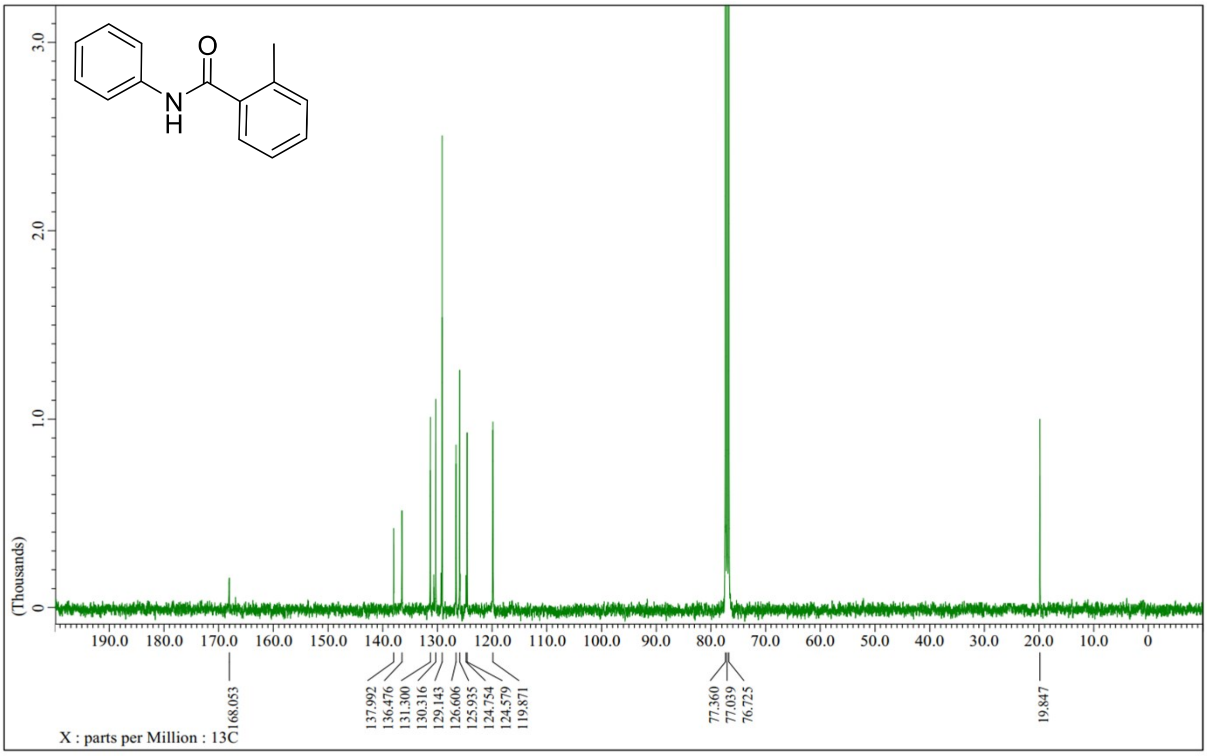

^{13}C NMR spectrum of 2-methyl-*N*-phenylbenzamide (3ah)

3-methoxy-*N*-phenylbenzamide (3ai)

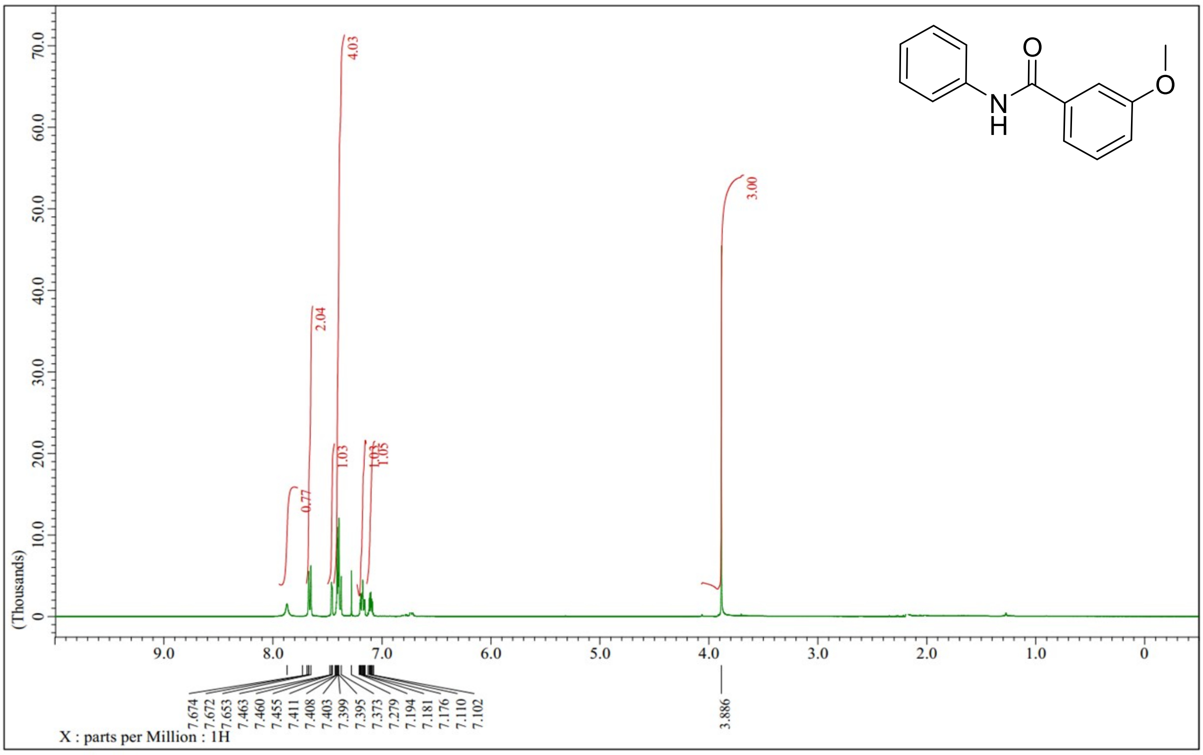

^{1}H NMR spectrum of 3-methoxy-*N*-phenylbenzamide (3ai)

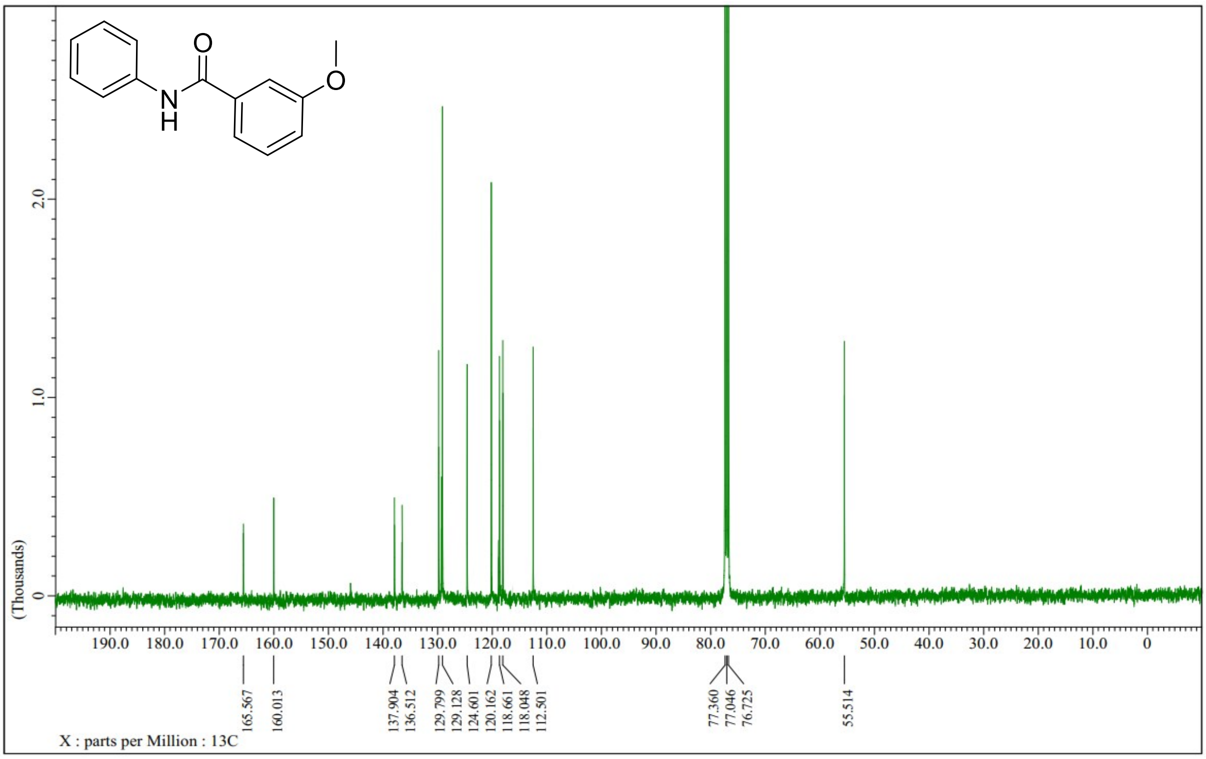

^{13}C NMR spectrum of 3-methoxy-*N*-phenylbenzamide (3ai)

N-phenyl-3-(trifluoromethyl)benzamide (3aj)

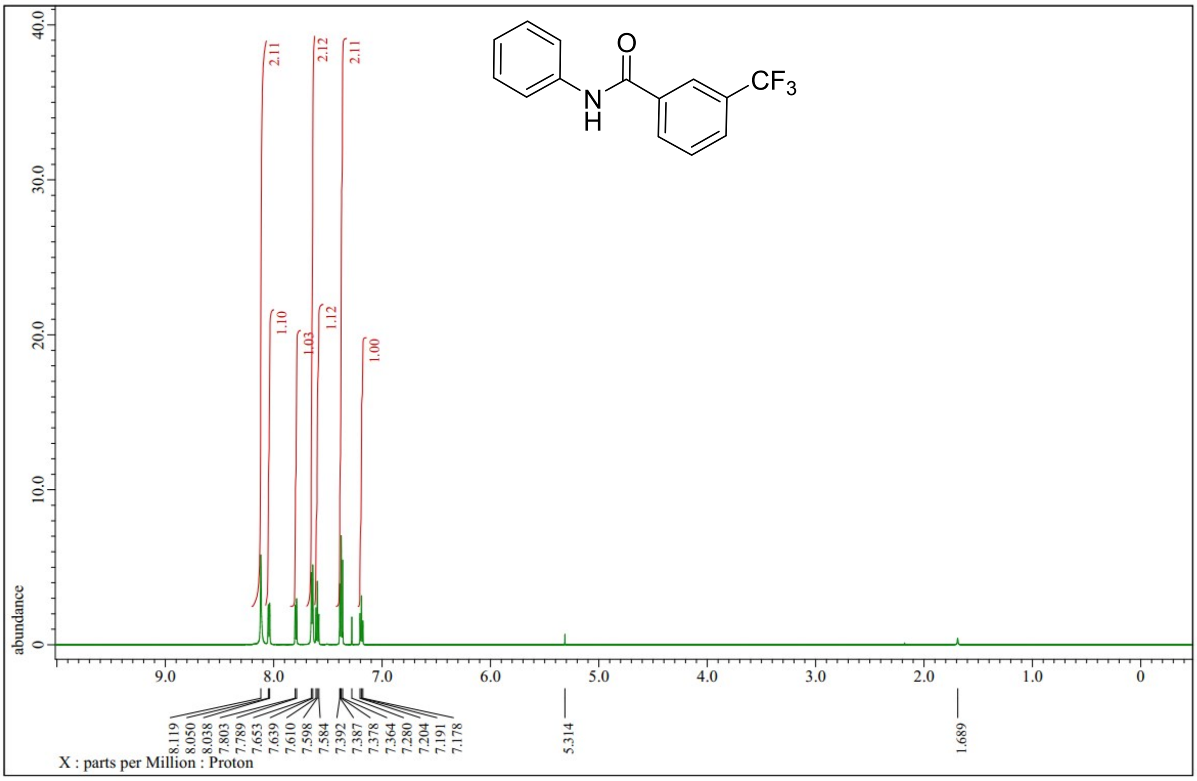

^{1}H NMR spectrum of ***N***-phenyl-3-(trifluoromethyl)benzamide (3aj)

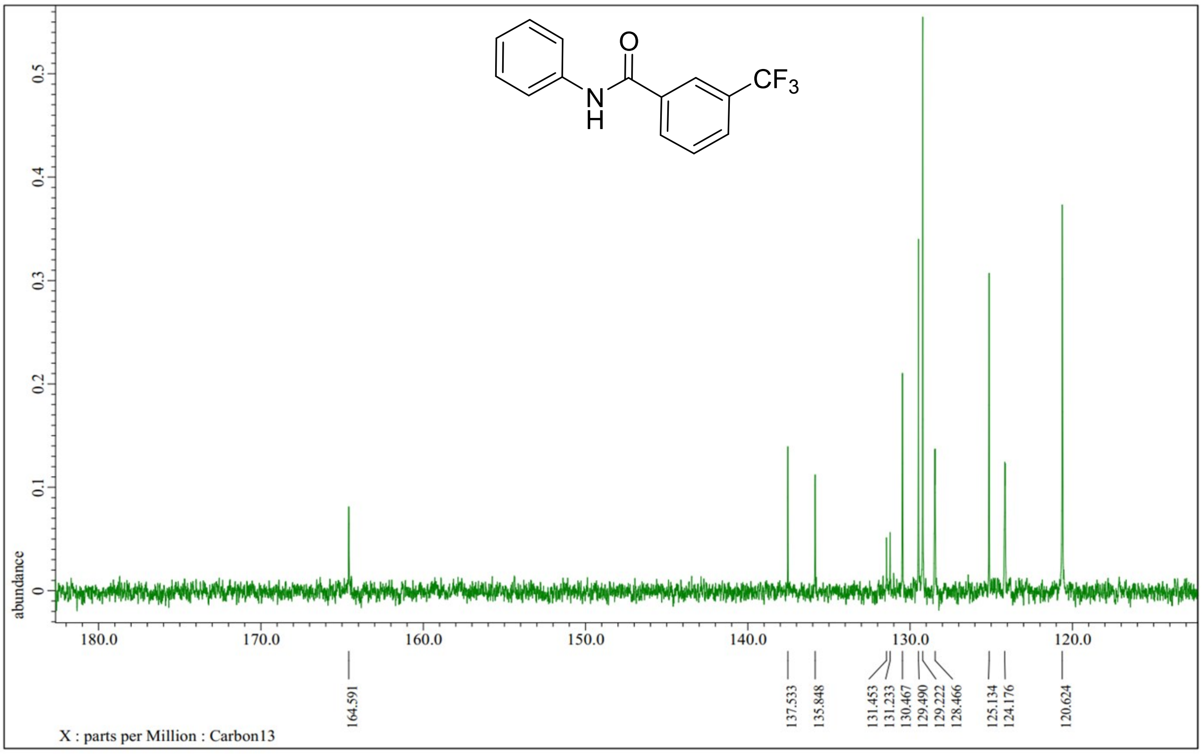

^{13}C NMR spectrum of *N*-phenyl-3-(trifluoromethyl)benzamide (3aj)

3-methyl-*N*-phenylbutanamide (3ak)

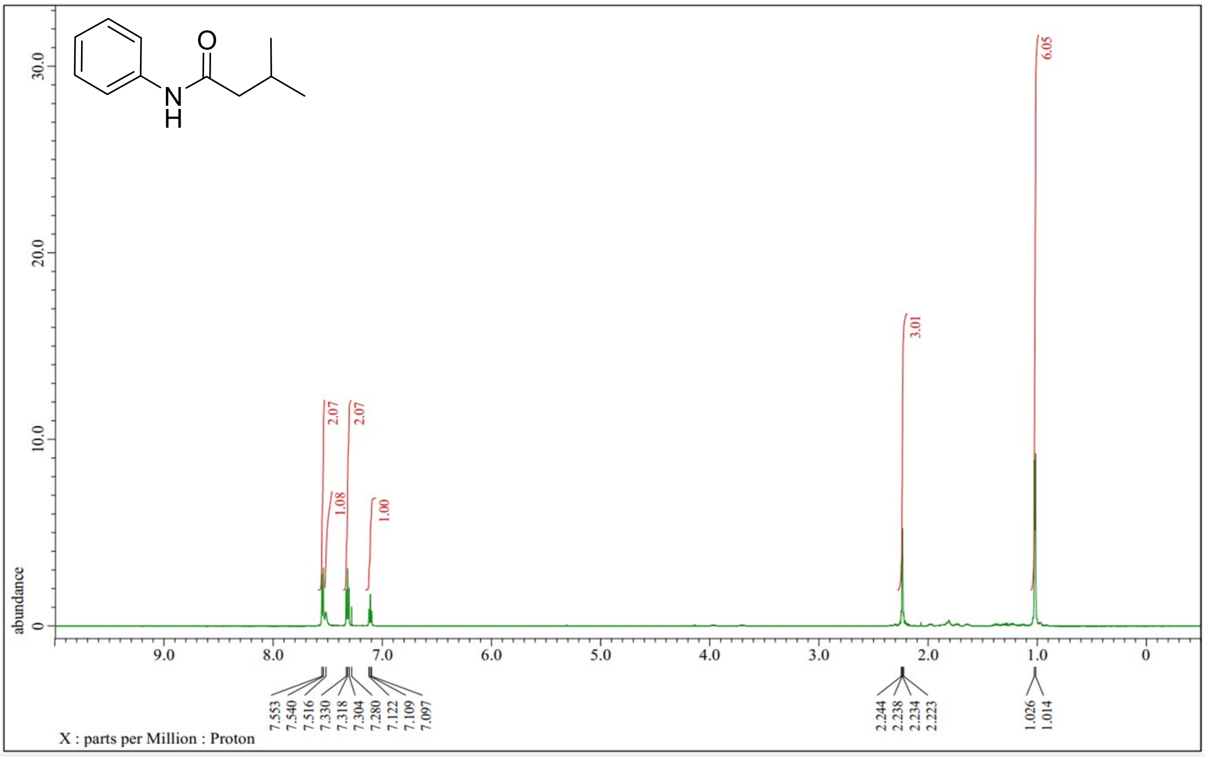

^{1}H NMR spectrum of 3-methyl-*N*-phenylbutanamide (3ak)

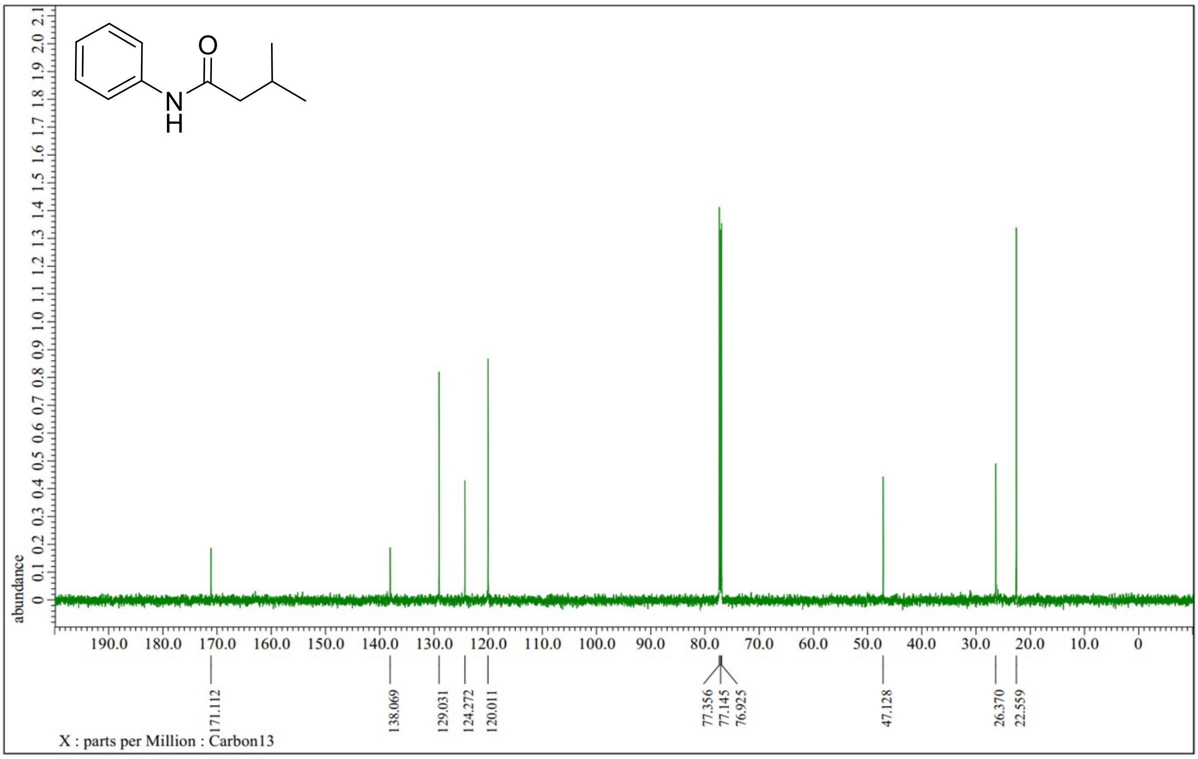

^{13}C NMR spectrum of 3-methyl-*N*-phenylbutanamide (3ak)

_N_-(3,5-dimethylphenyl)-3-methylbutanamide (3al)

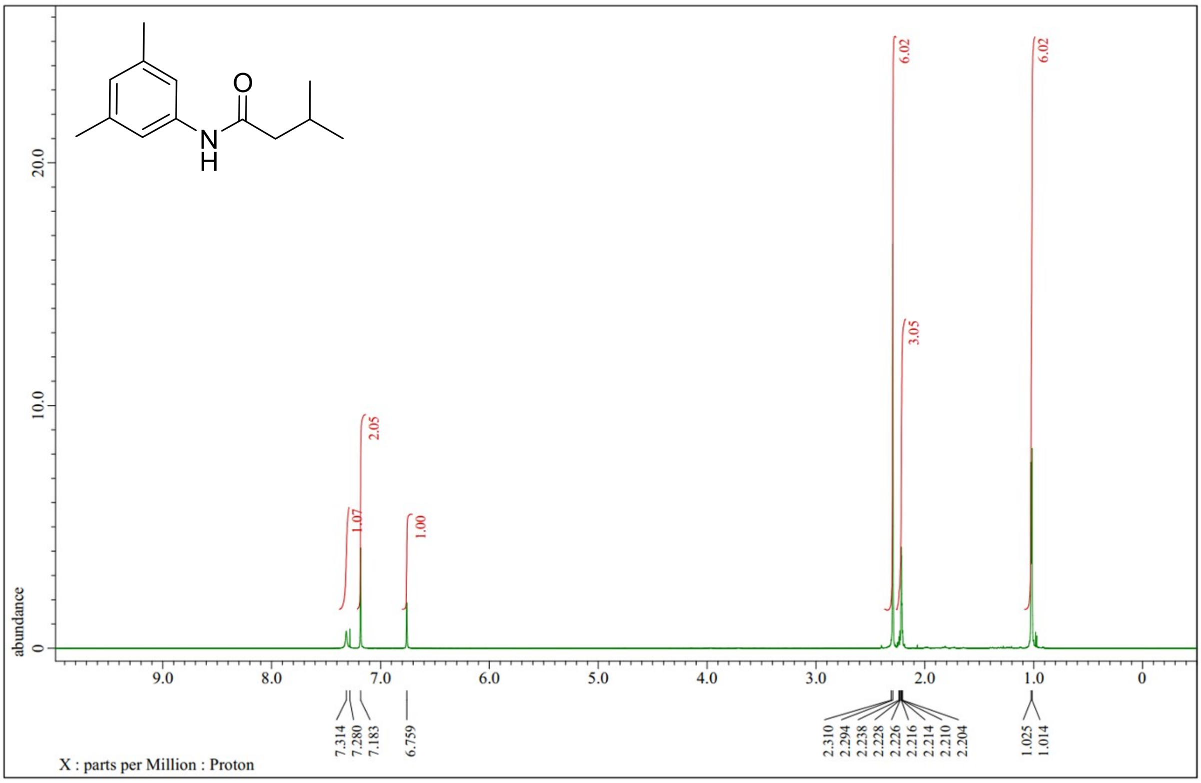

^{1}H NMR spectrum of *N*-(3,5-dimethylphenyl)-3-methylbutanamide (3al)

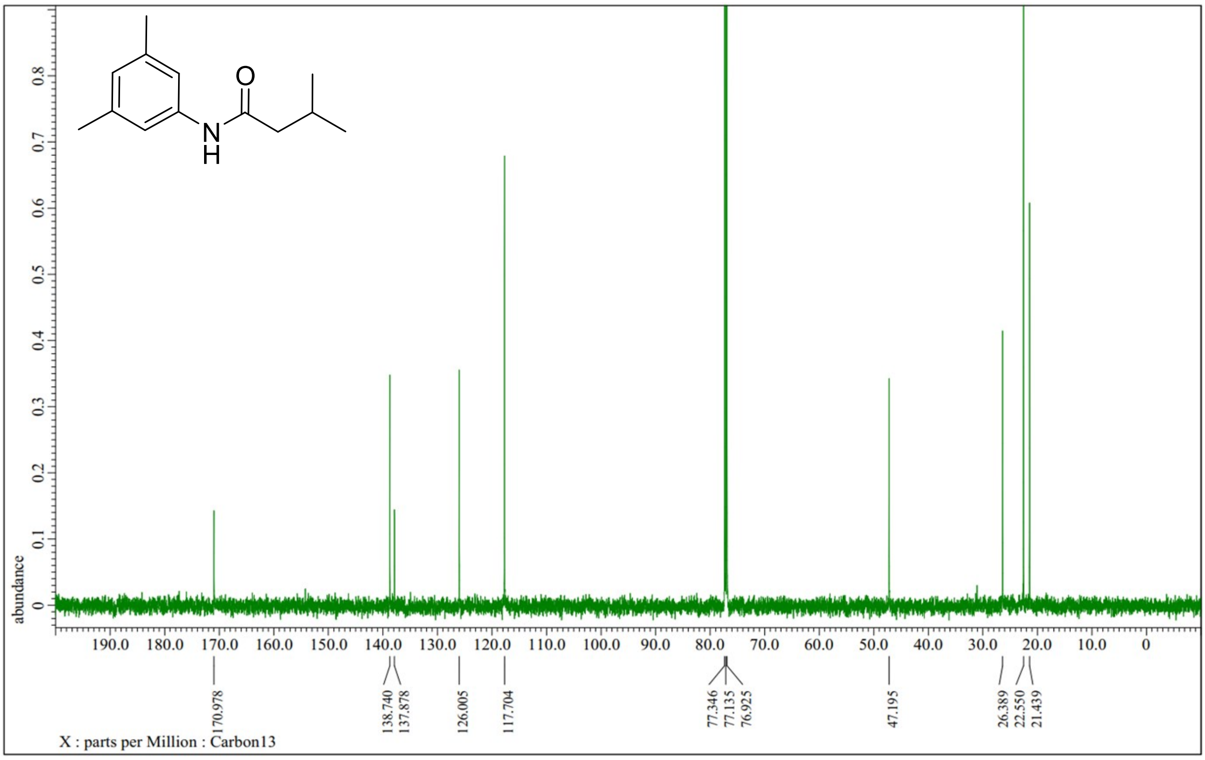

^{13}C NMR spectrum of *N*-(3,5-dimethylphenyl)-3-methylbutanamide (3al)

***N*-(*p*-tolyl)cyclohexanecarboxamide (3am)**

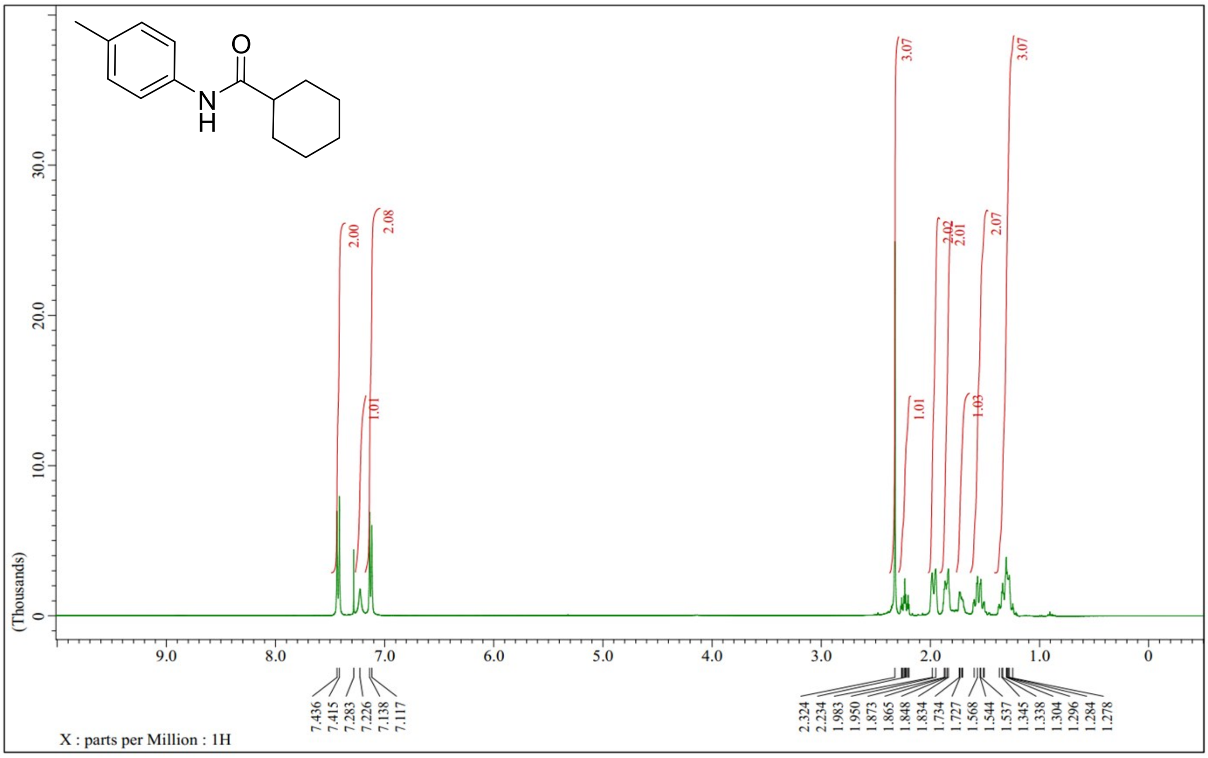

^{1}H NMR spectrum of *N*-(*p*-tolyl)cyclohexanecarboxamide (3am)

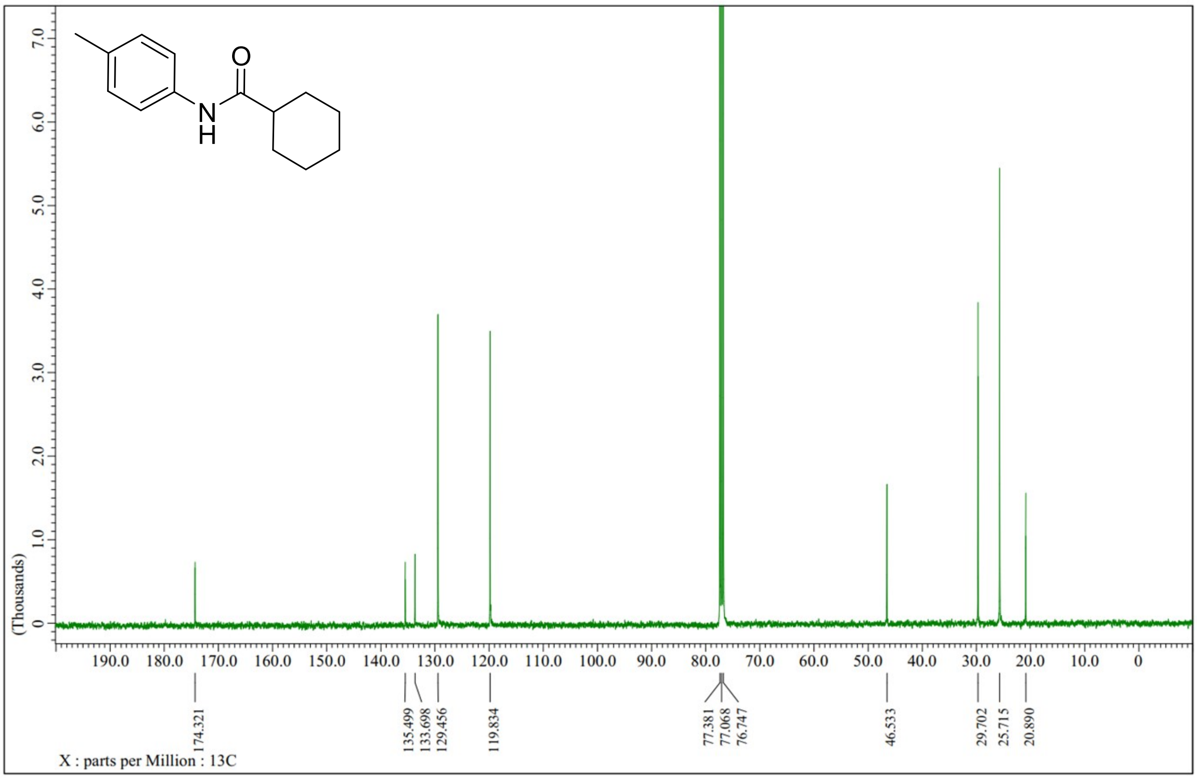

^{13}C NMR spectrum of *N*-(*p*-tolyl)cyclohexanecarboxamide (3am)

***N*-(4-chlorophenyl)-3-methylbutanamide (3an)**

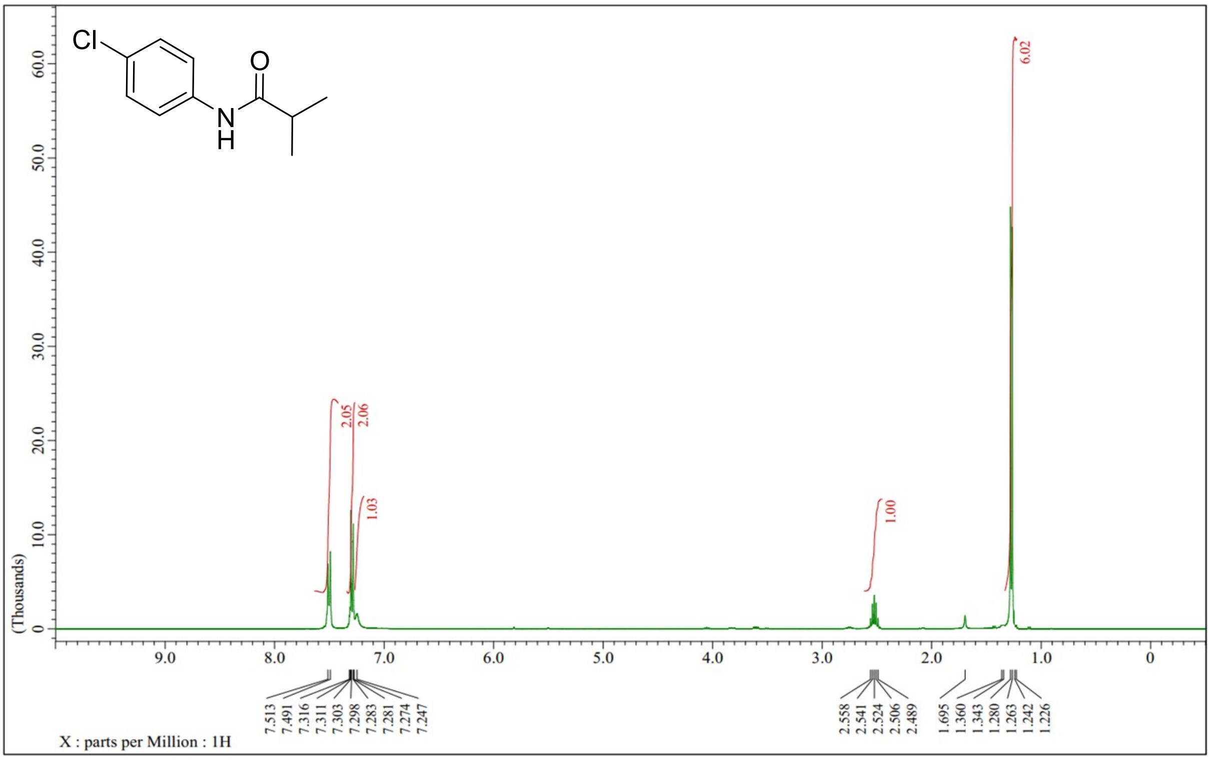

^{1}H NMR spectrum of *N*-(4-chlorophenyl)-3-methylbutanamide (3an)

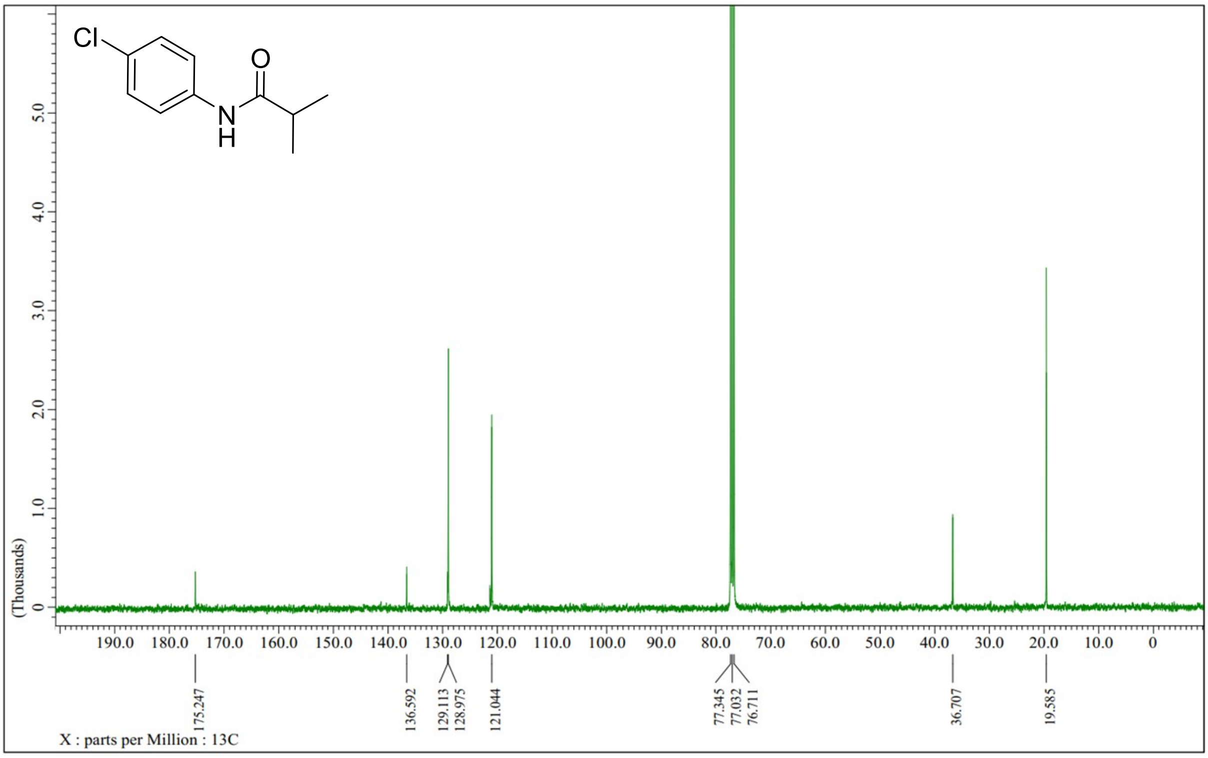

^{13}C NMR spectrum of *N*-(4-chlorophenyl)-3-methylbutanamide (5k)

***N*-(4-cyanophenyl)butyramide (3ao)**

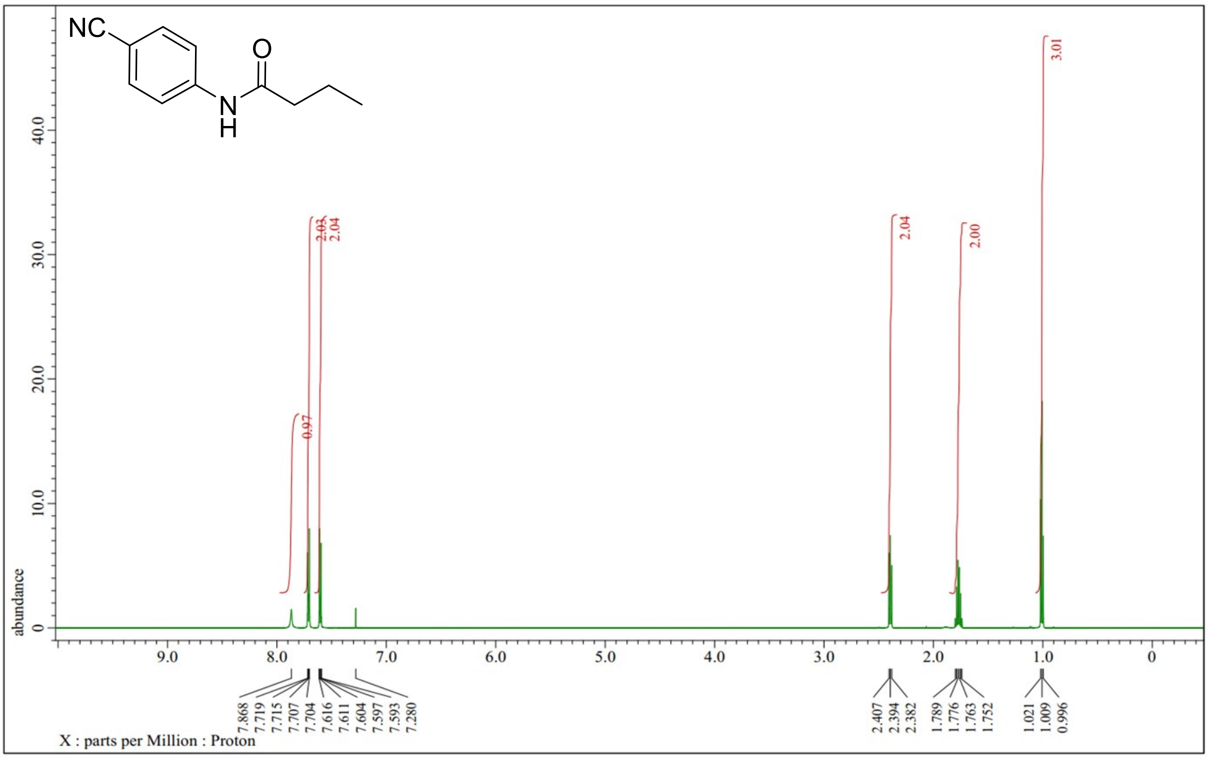

^{1}H NMR spectrum of *N*-(4-cyanophenyl)butyramide (3ao)

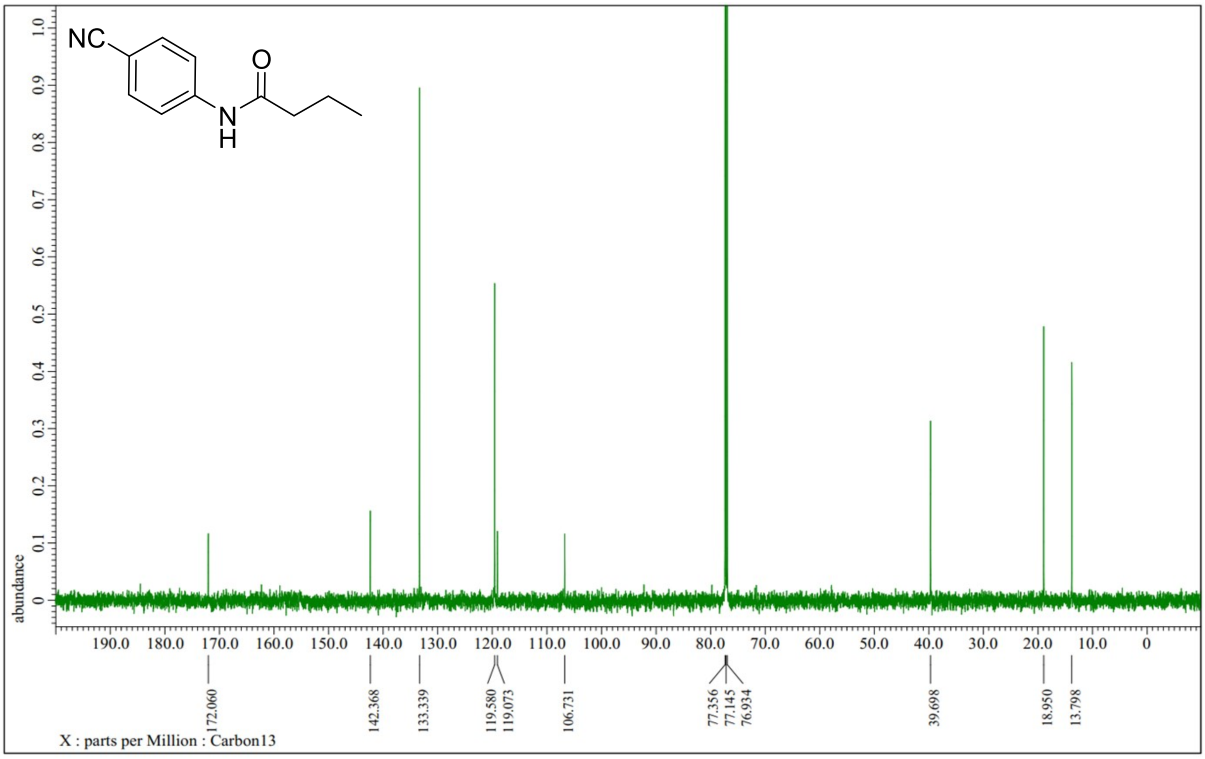

^{13}C NMR spectrum of *N*-(4-cyanophenyl)butyramide (3ao)

***N*-benzyl-3-phenylpropanamide (3ap)**

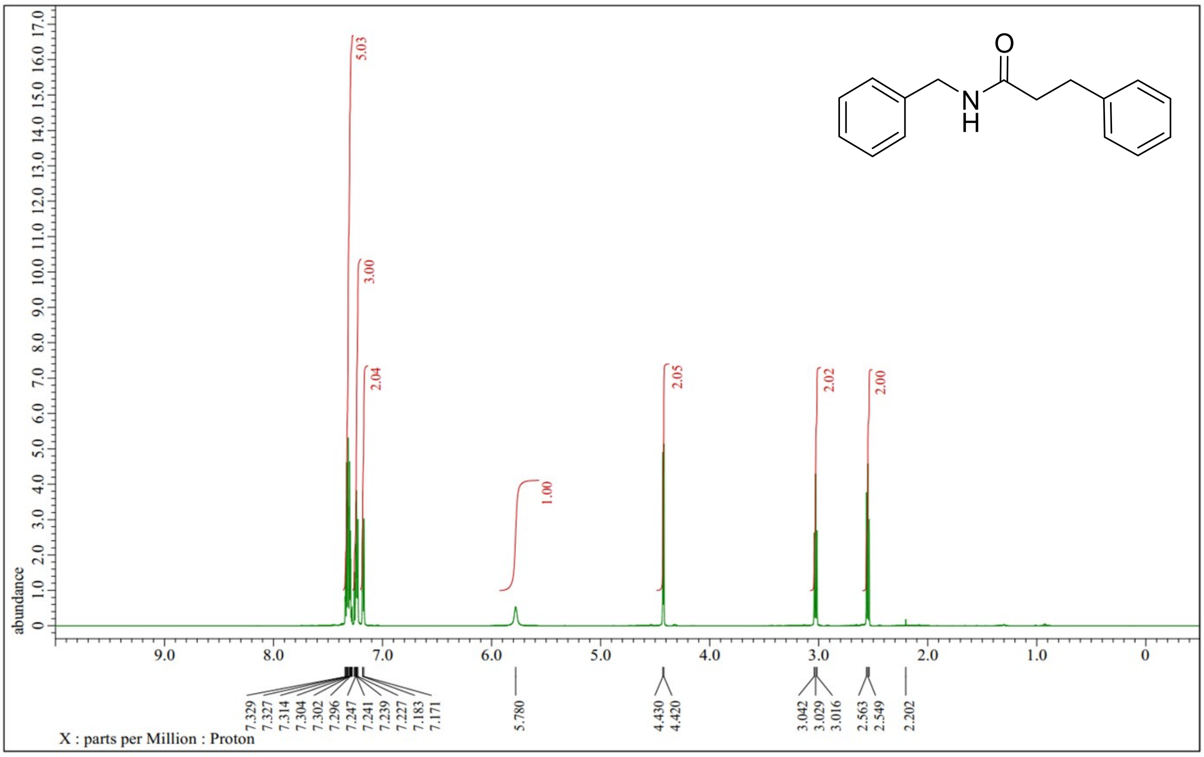

^{1}H NMR spectrum of *N*-benzyl-3-phenylpropanamide (3ap)

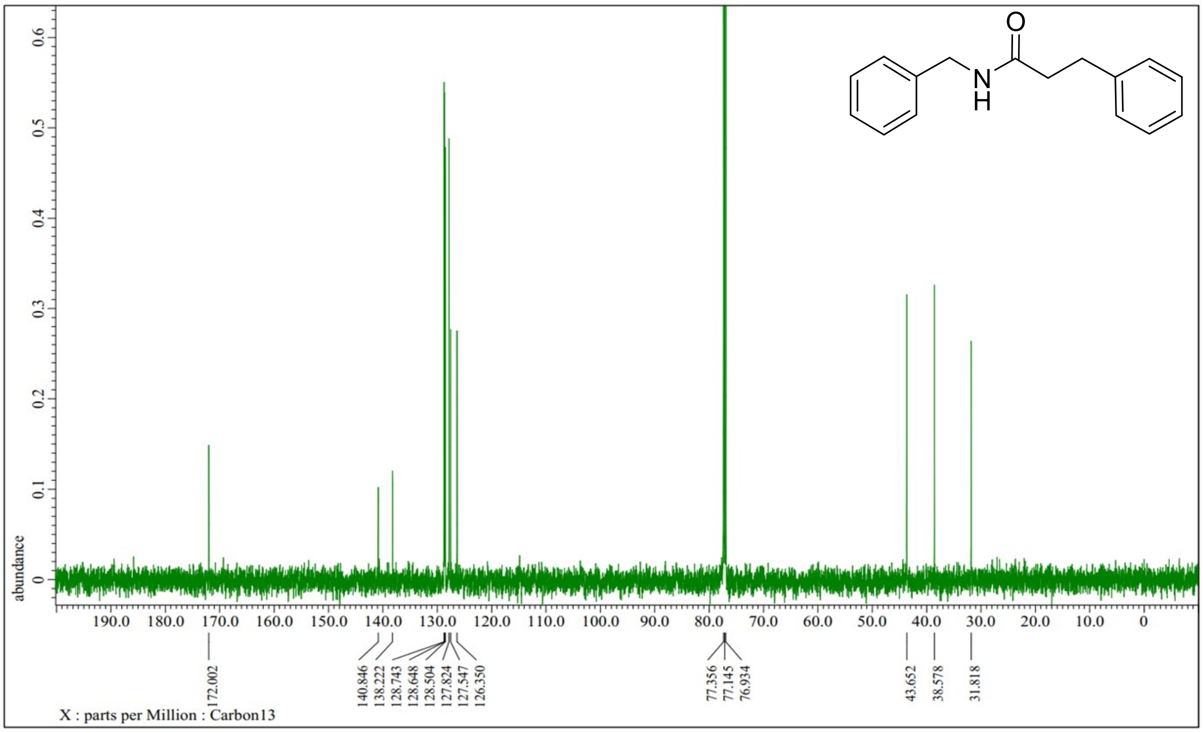

^{13}C NMR spectrum of *N*-benzyl-3-phenylpropanamide (3ap)

N-isobutyl-1-naphthamide (3aq)

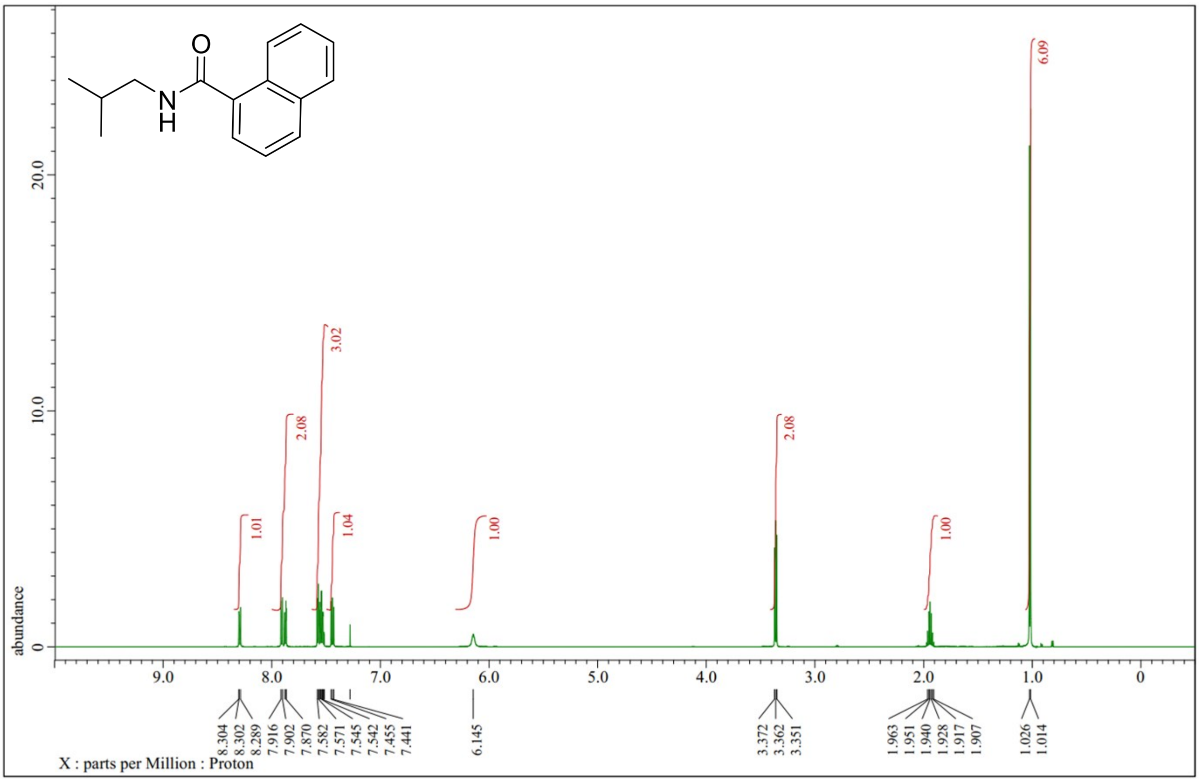

^{1}H NMR spectrum of N-isobutyl-1-naphthamide (3aq)

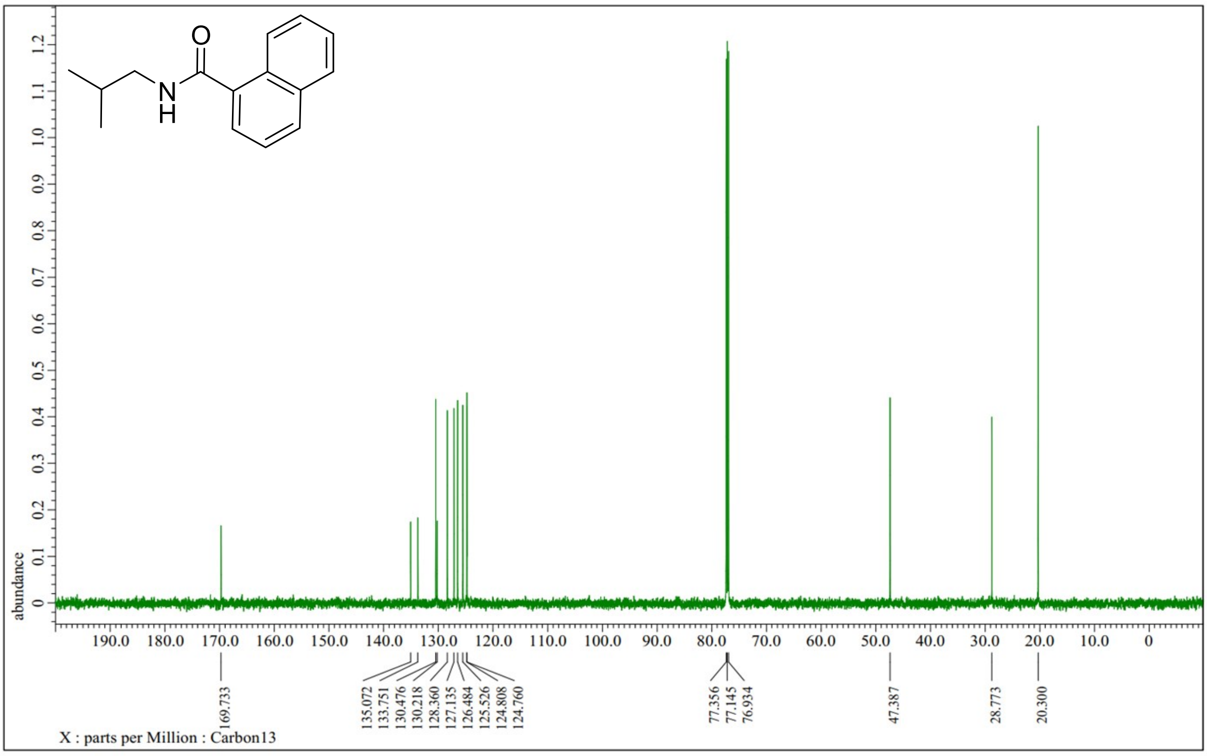

^{13}C NMR spectrum of N-isobutyl-1-naphthamide (3aq)

***N*-cyclohexyl-3-phenylpropanamide (3ar)**

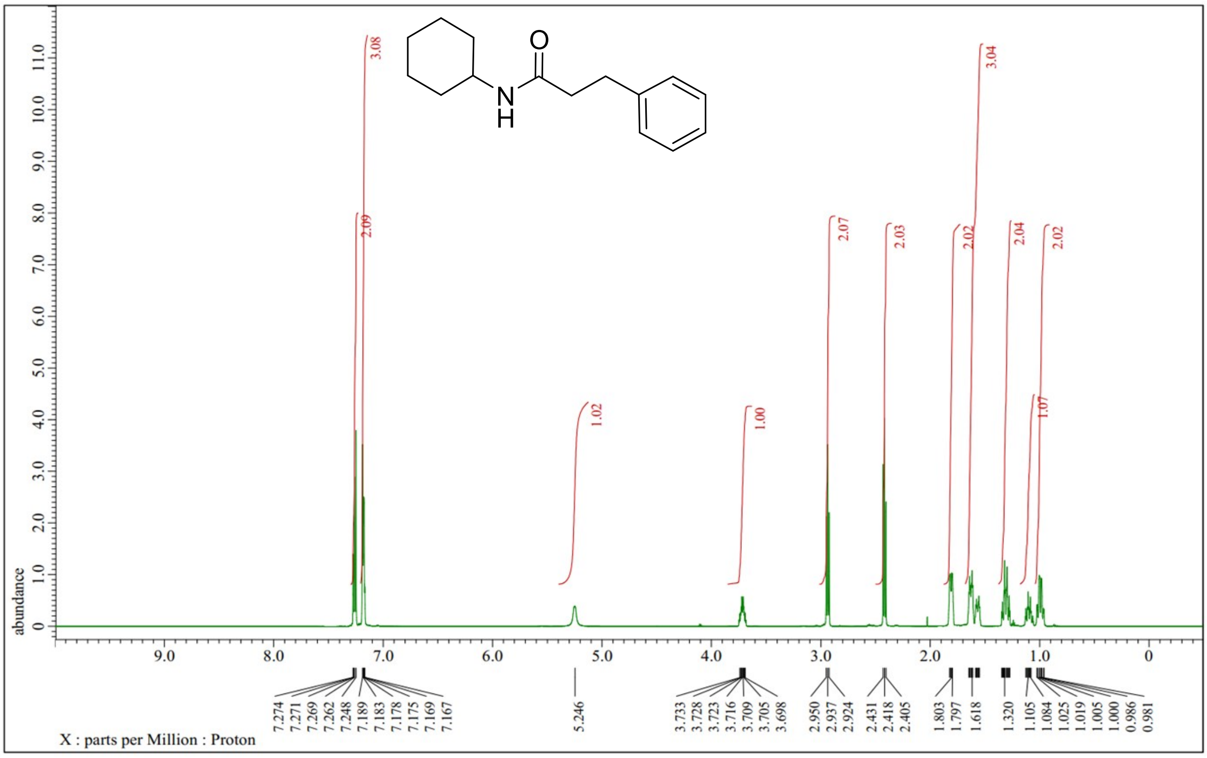

^{1}H NMR spectrum of *N*-cyclohexyl-3-phenylpropanamide (3ar)

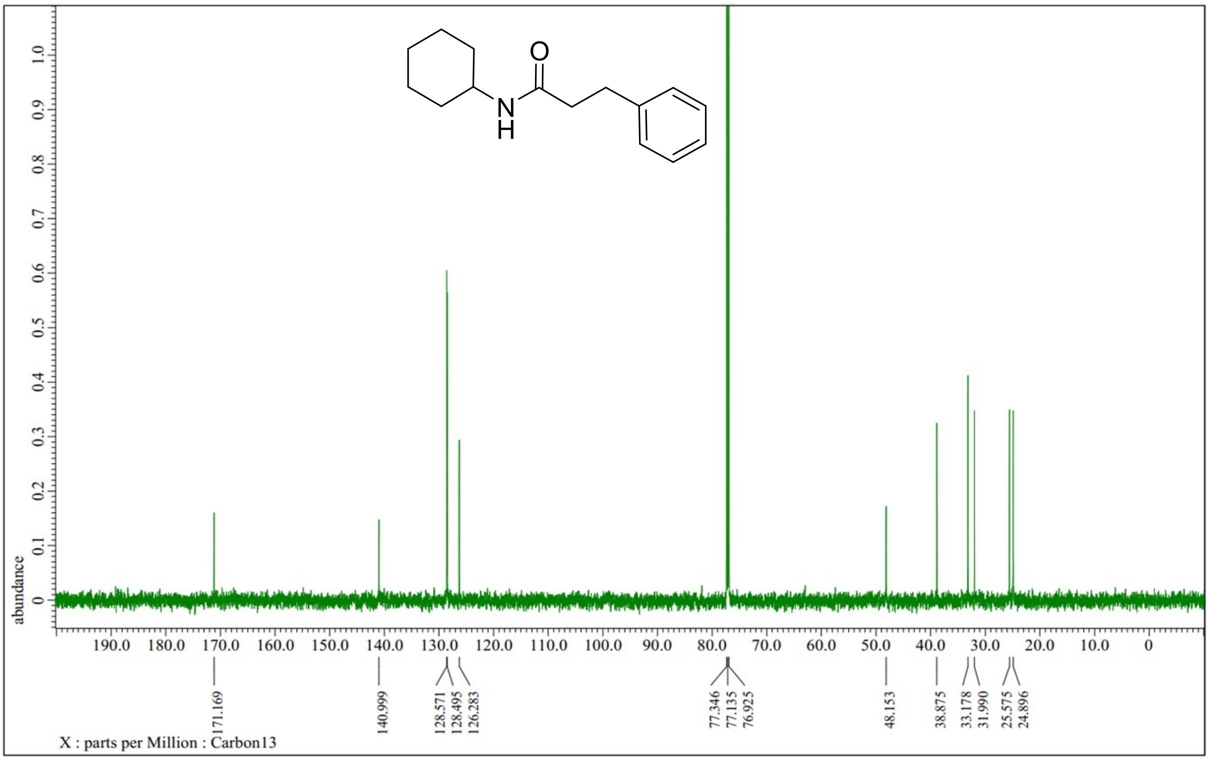

^{13}C NMR spectrum of *N*-cyclohexyl-3-phenylpropanamide (3ar)

***N*-3-diphenylpropanamide (3as)**

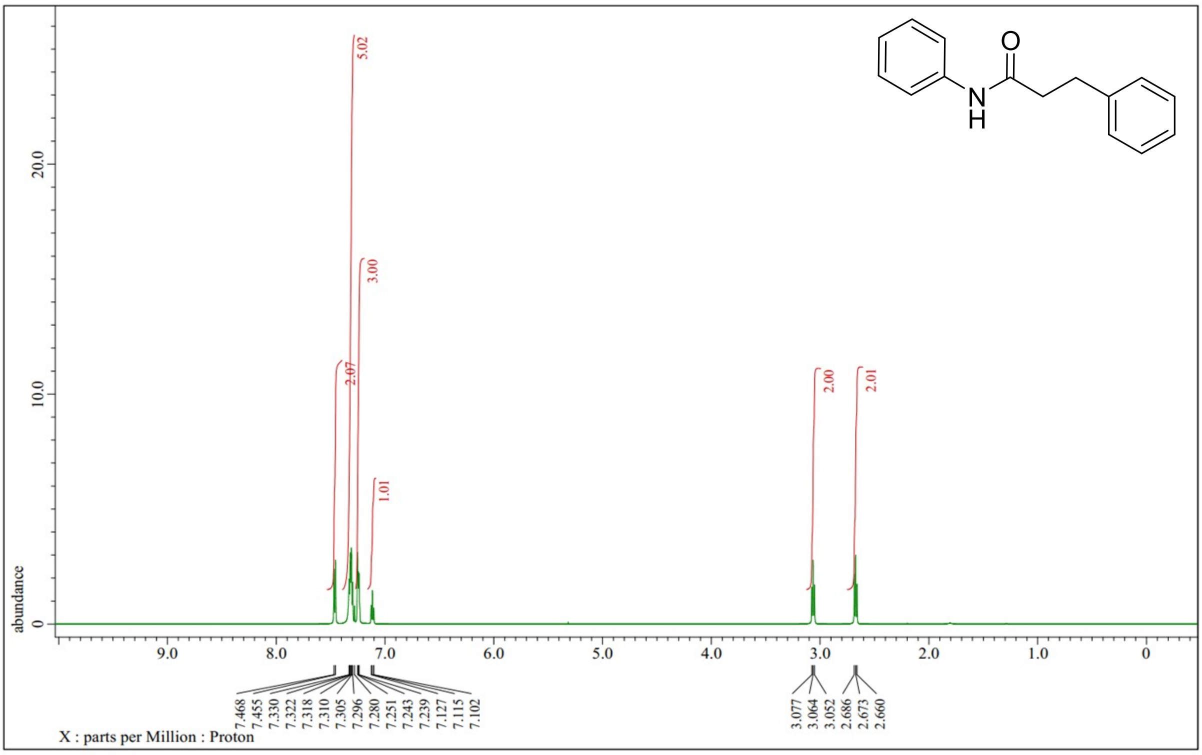

^{1}H NMR spectrum of *N*,3-diphenylpropanamide (3as)

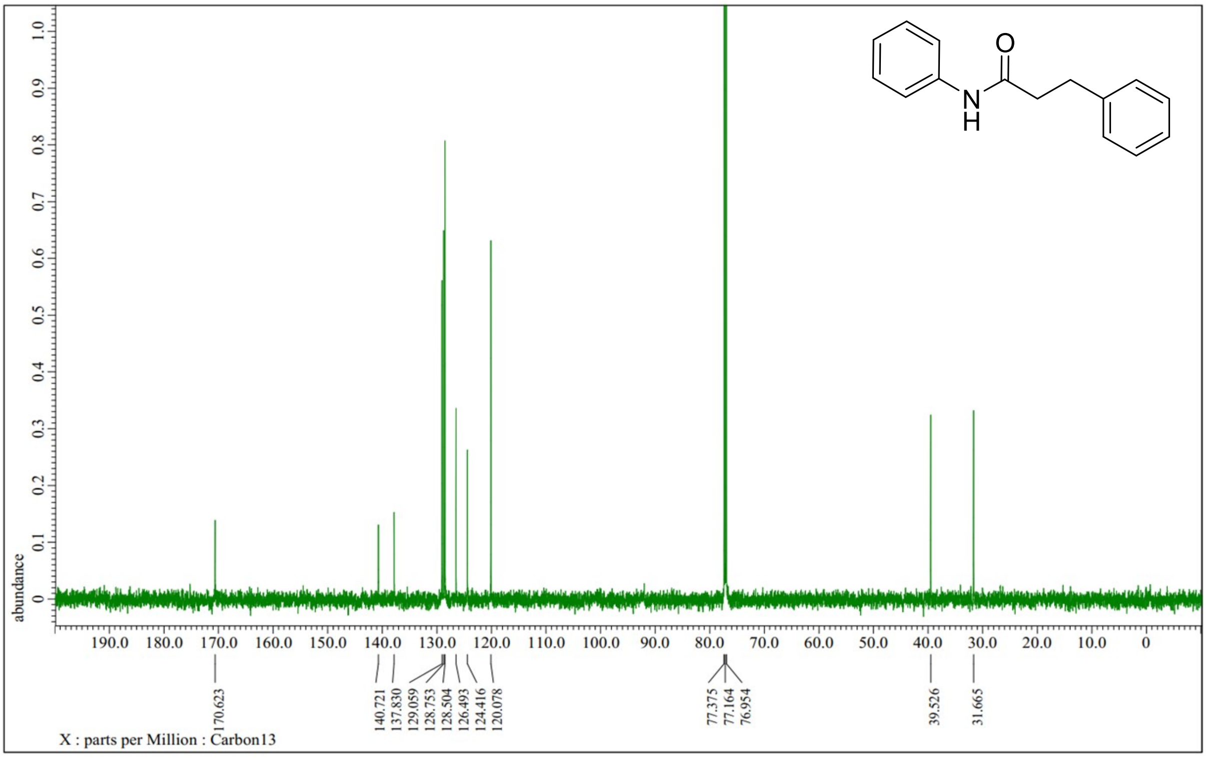

^{13}C NMR spectrum of *N*,3-diphenylpropanamide (3as)

***N*-(3,5-dimethylphenyl)-2-methylbenzamide (3at)**

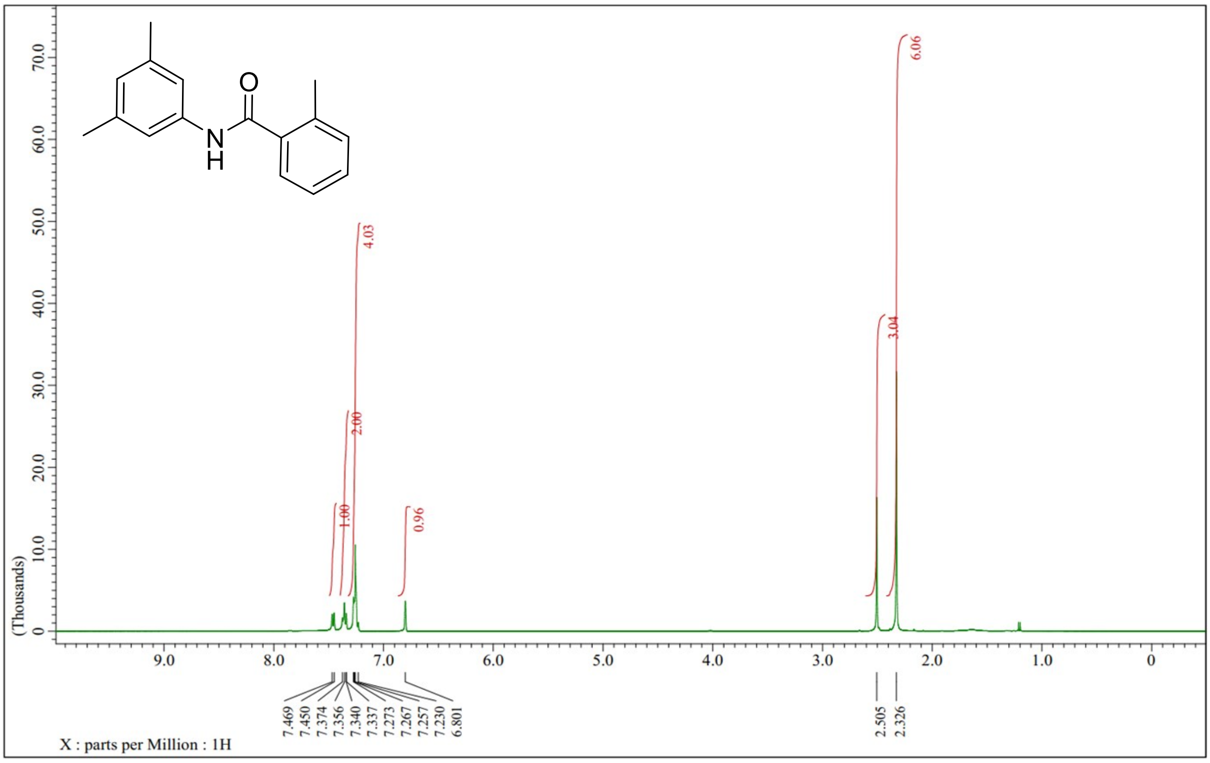

^{1}H NMR spectrum of *N*-(3,5-dimethylphenyl)-2-methylbenzamide (3at)

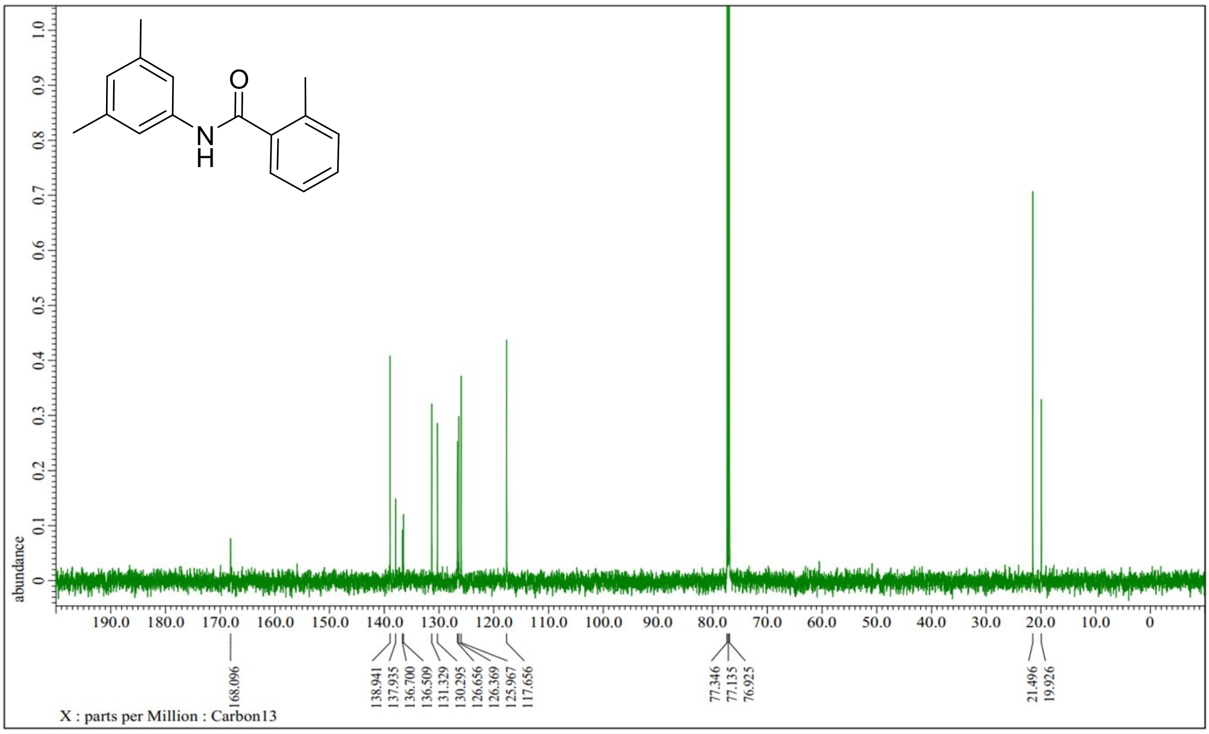

^{13}C NMR spectrum of *N*-(3,5-dimethylphenyl)-2-methylbenzamide (3at)

***N*-(3,5-dimethylphenyl)-3-methoxybenzamide (3au)**

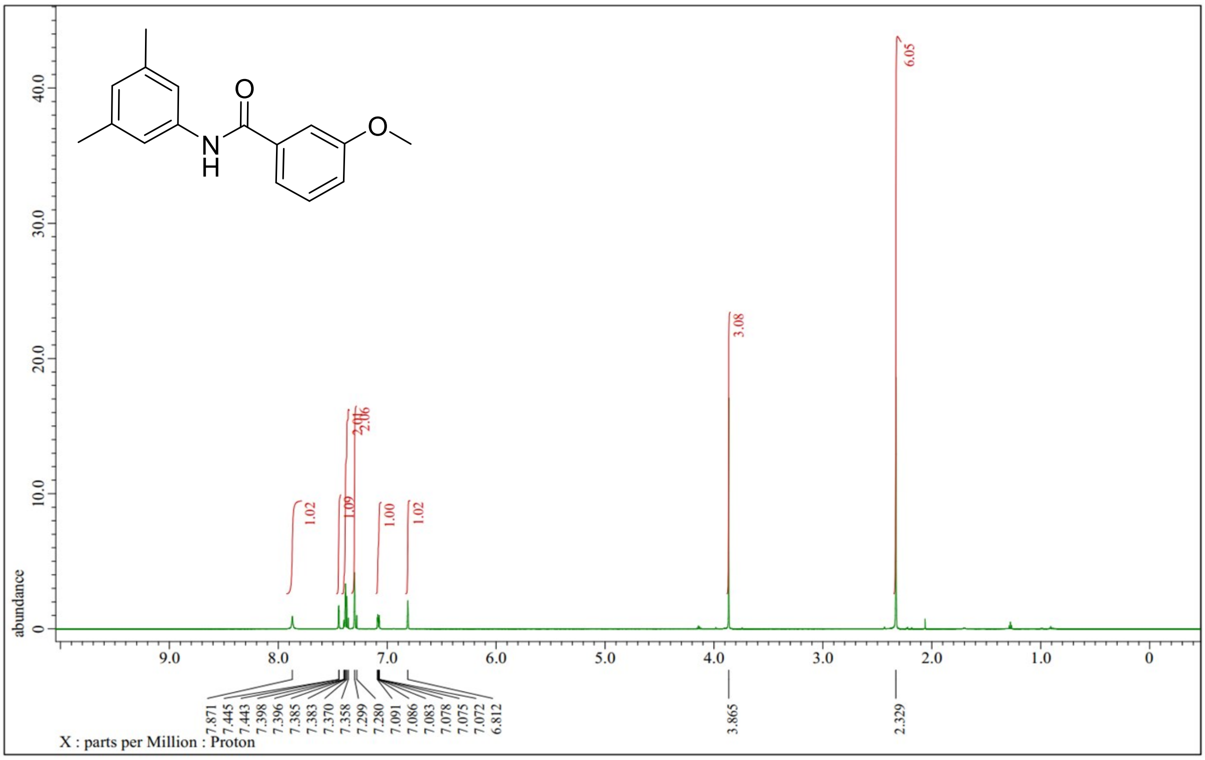

^{1}H NMR spectrum of *N*-(3,5-dimethylphenyl)-3-methoxybenzamide (3au)

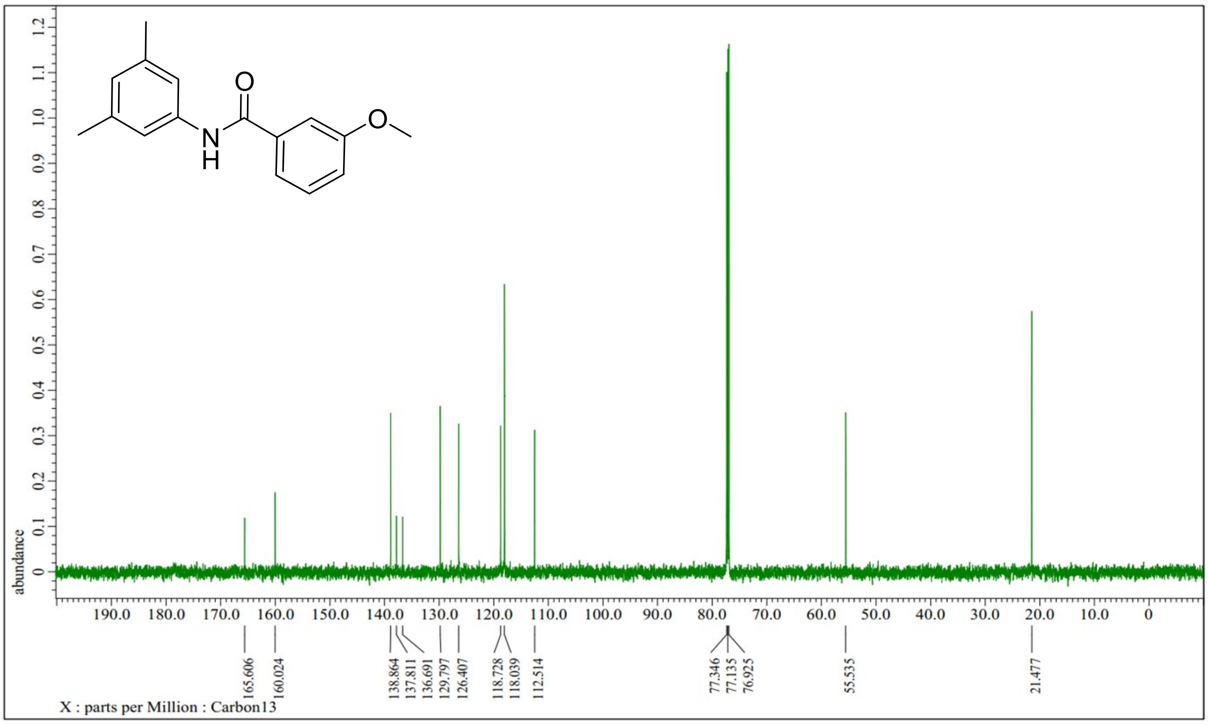

^{13}C NMR spectrum of *N*-(3,5-dimethylphenyl)-3-methoxybenzamide (3au)

4-chloro-*N*-(3,5-dimethylphenyl)benzamide (3av)

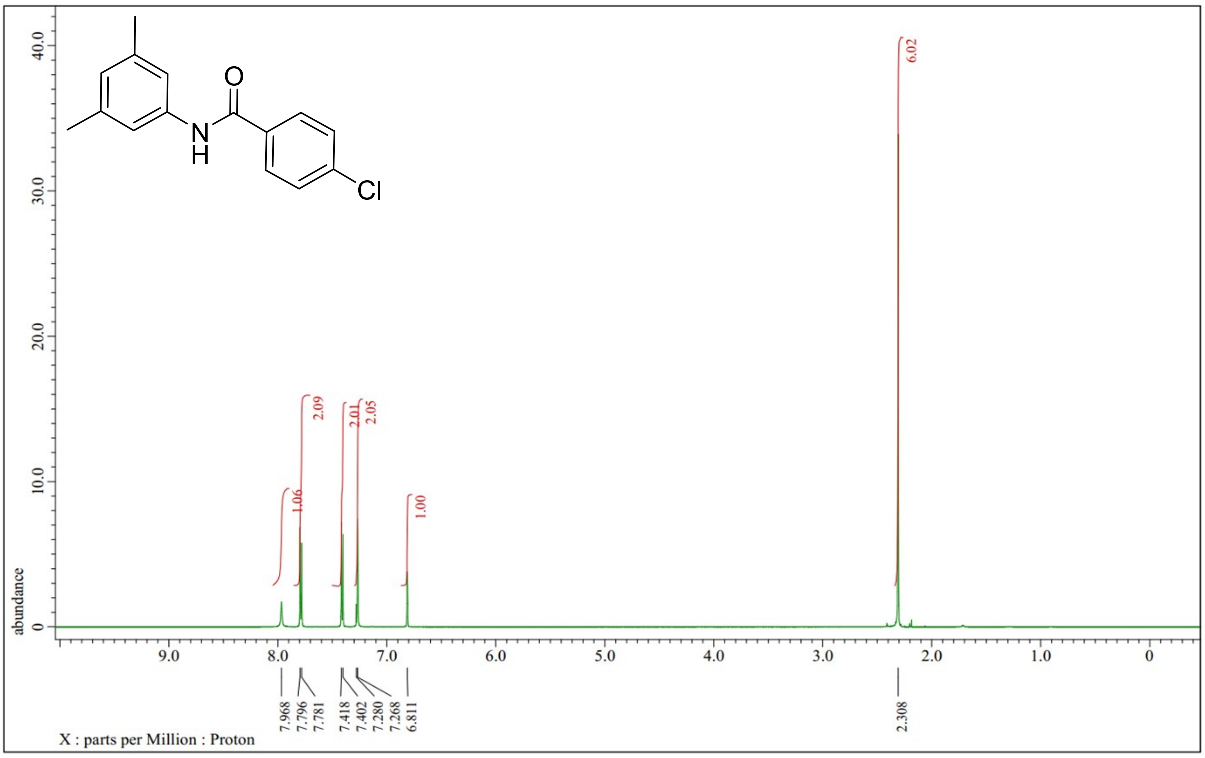

^{1}H NMR spectrum of 4-chloro-*N*-(3,5-dimethylphenyl)benzamide (3av)

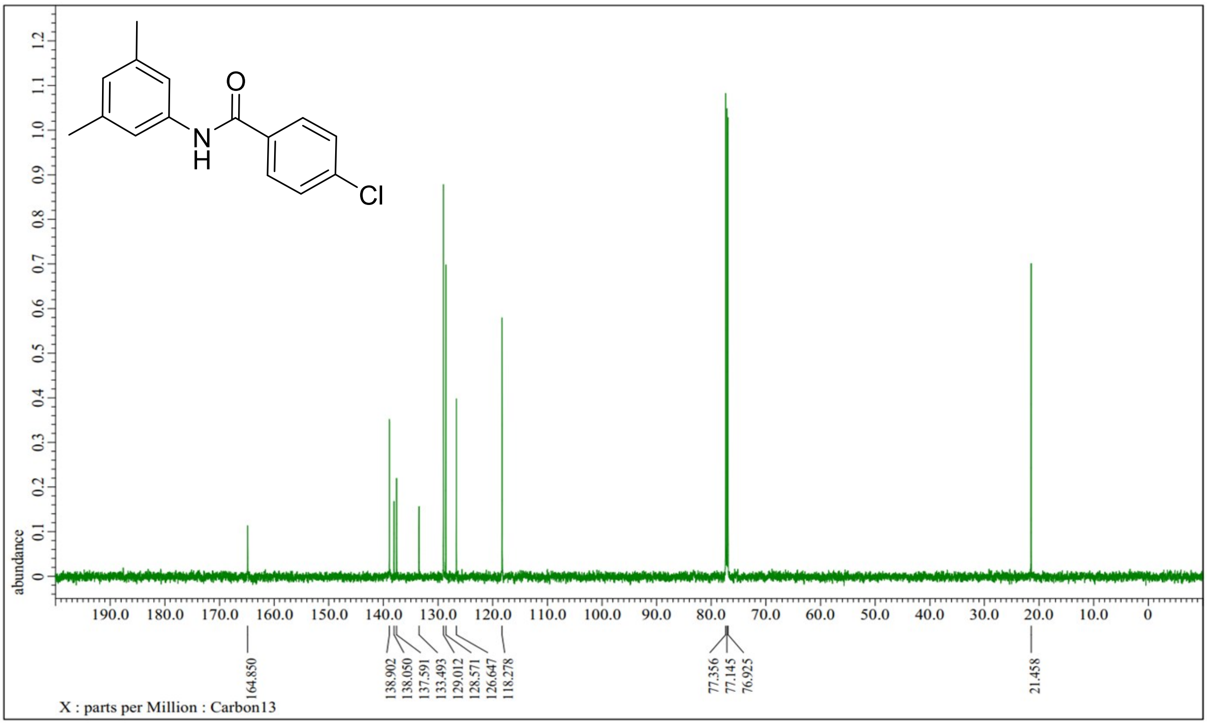

^{13}C NMR spectrum of 4-chloro-*N*-(3,5-dimethylphenyl)benzamide (3av)

***N*-(*p*-tolyl)butyramide (3aw)**

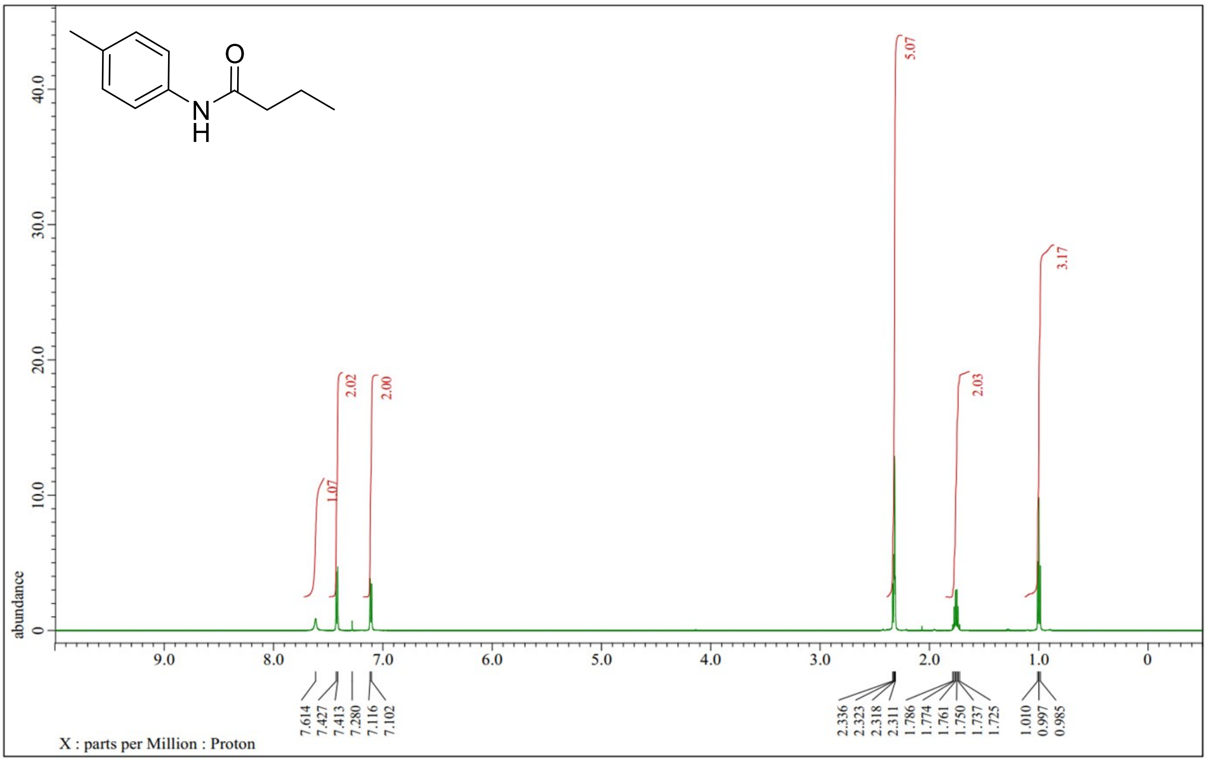

^{1}H NMR spectrum of *N*-(*p*-tolyl)butyramide (3aw)

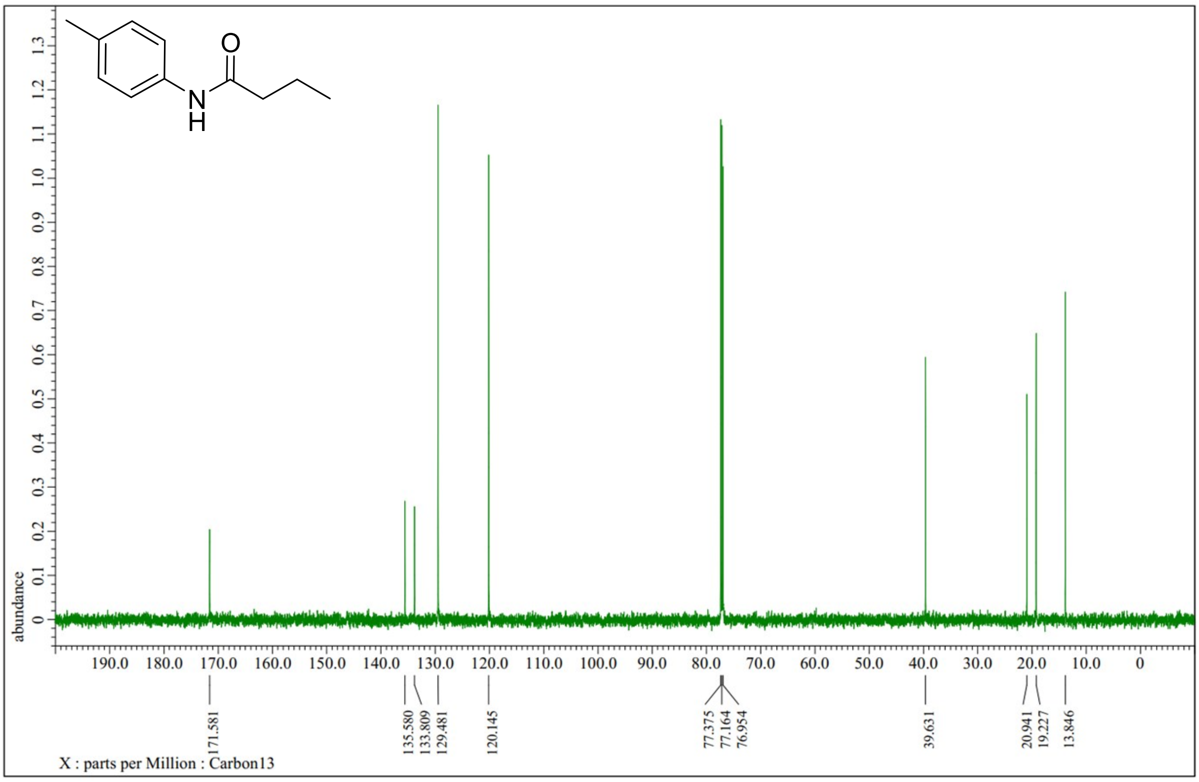

^{13}C NMR spectrum of *N*-(*p*-tolyl)butyramide (3aw)

N-(4-cyanophenyl)-3-phenylpropanamide (3ax)

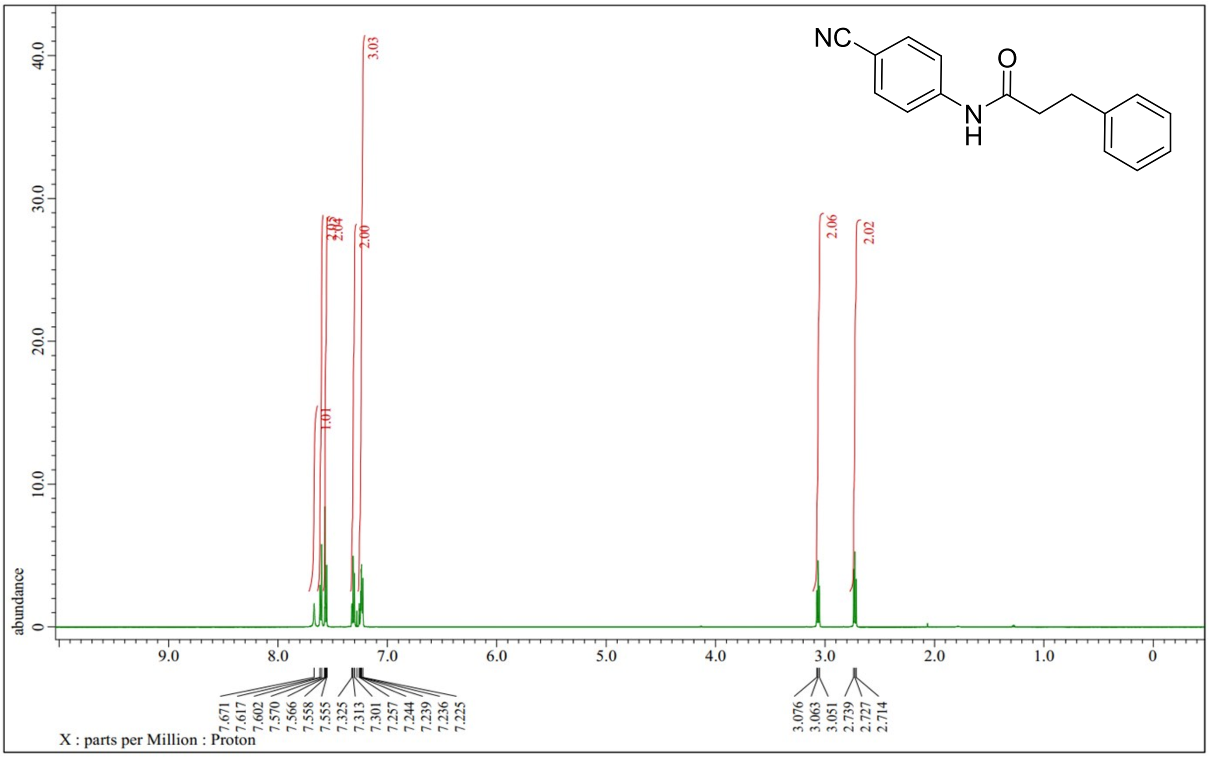

^{1}H NMR spectrum of *N*-(4-cyanophenyl)-3-phenylpropanamide (3ax)

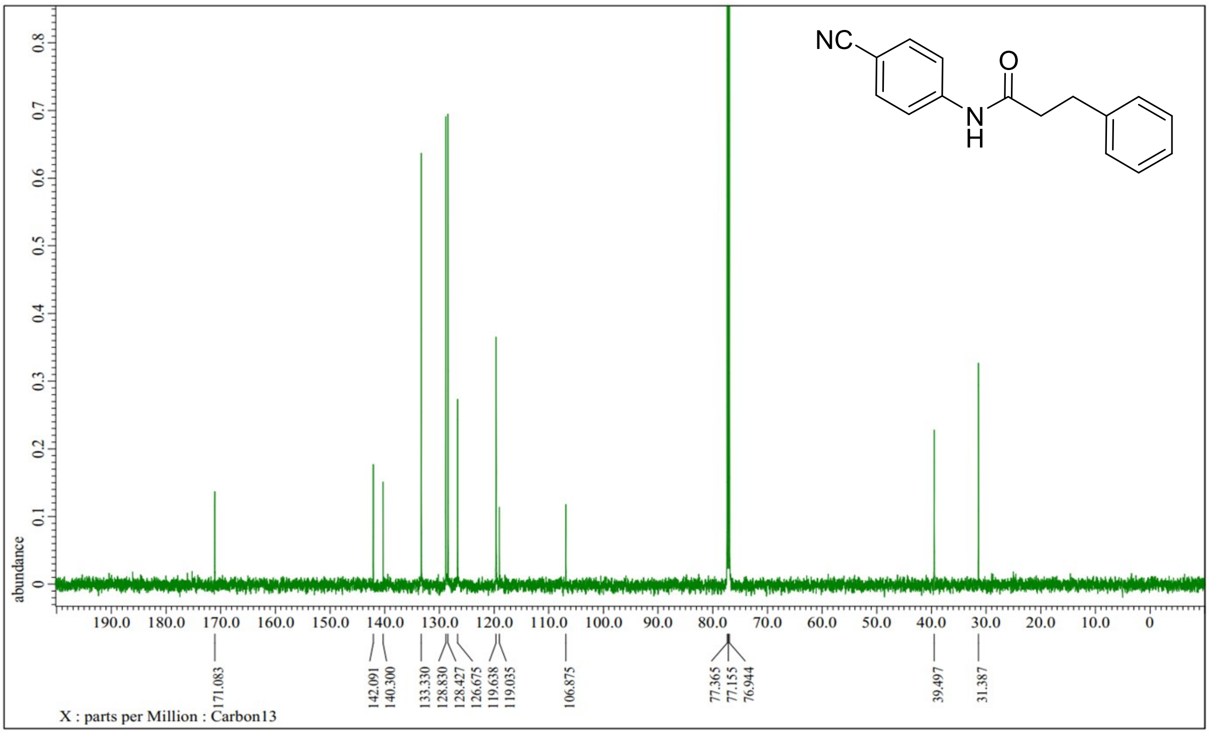

^{13}C NMR spectrum of *N*-(4-cyanophenyl)-3-phenylpropanamide (3ax)

***N*-benzylbutyramide (3ay)**

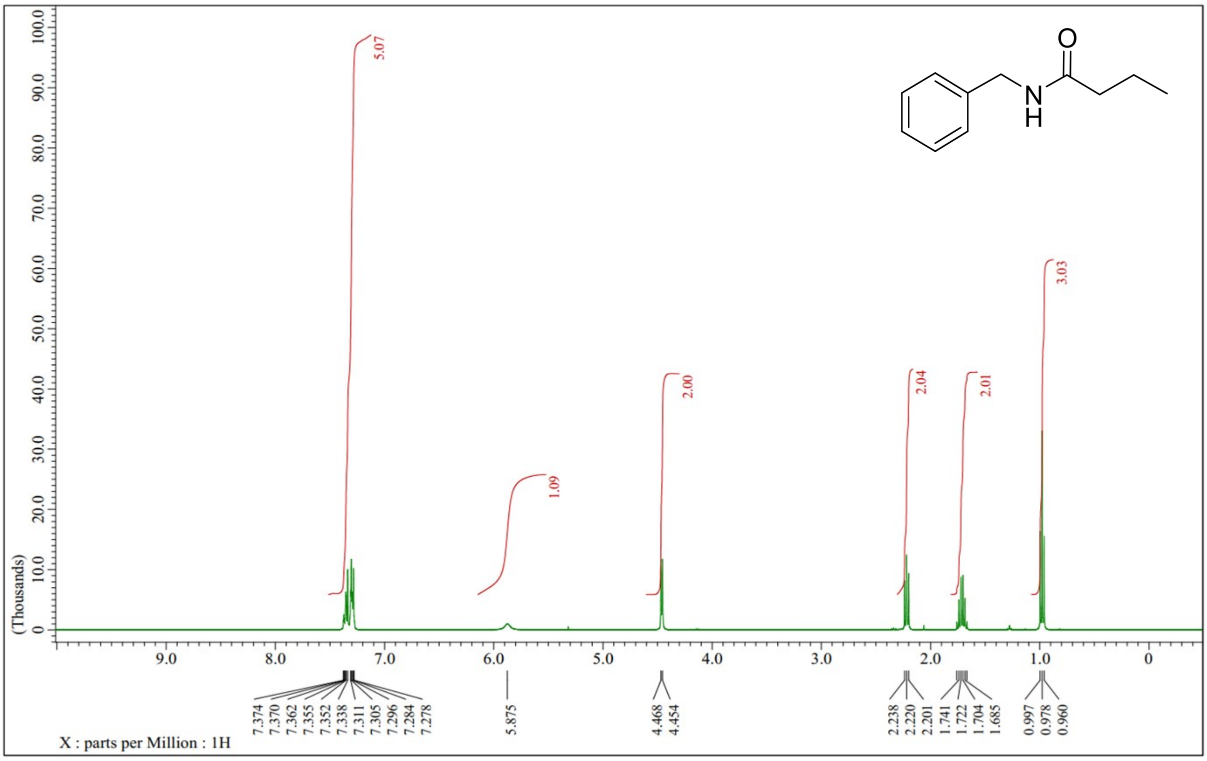

^{1}H NMR spectrum of *N*-benzylbutyramide (3ay)

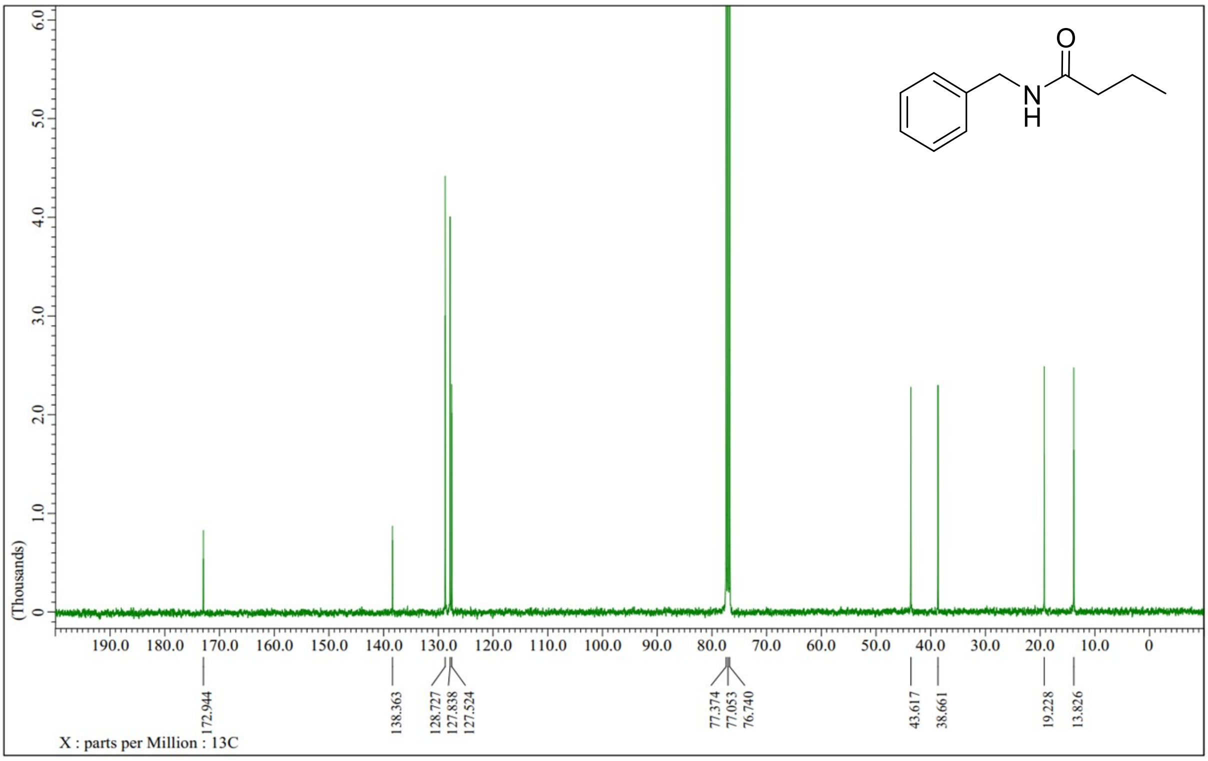

^{13}C NMR spectrum of *N*-benzylbutyramide (3ay)

N-cyclohexyl-4-methylbenzamide (3az)

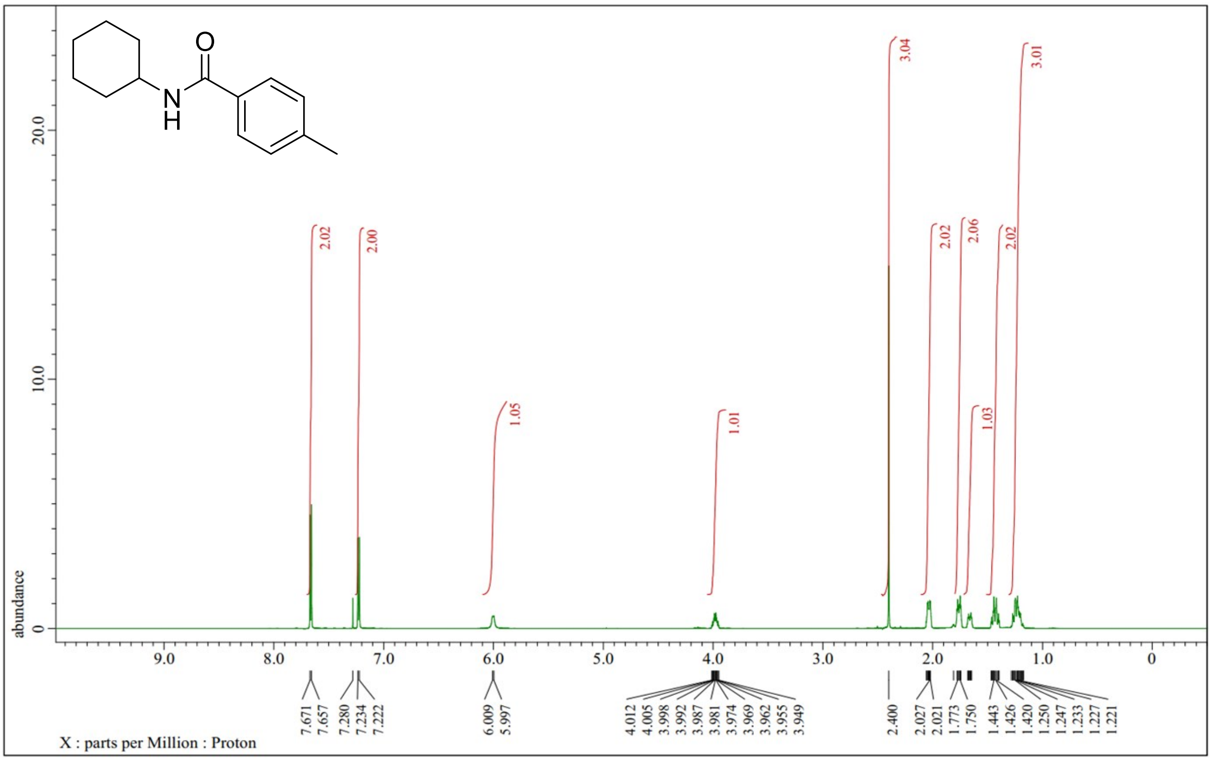

^{1}H NMR spectrum of *N*-cyclohexyl-4-methylbenzamide (3az)

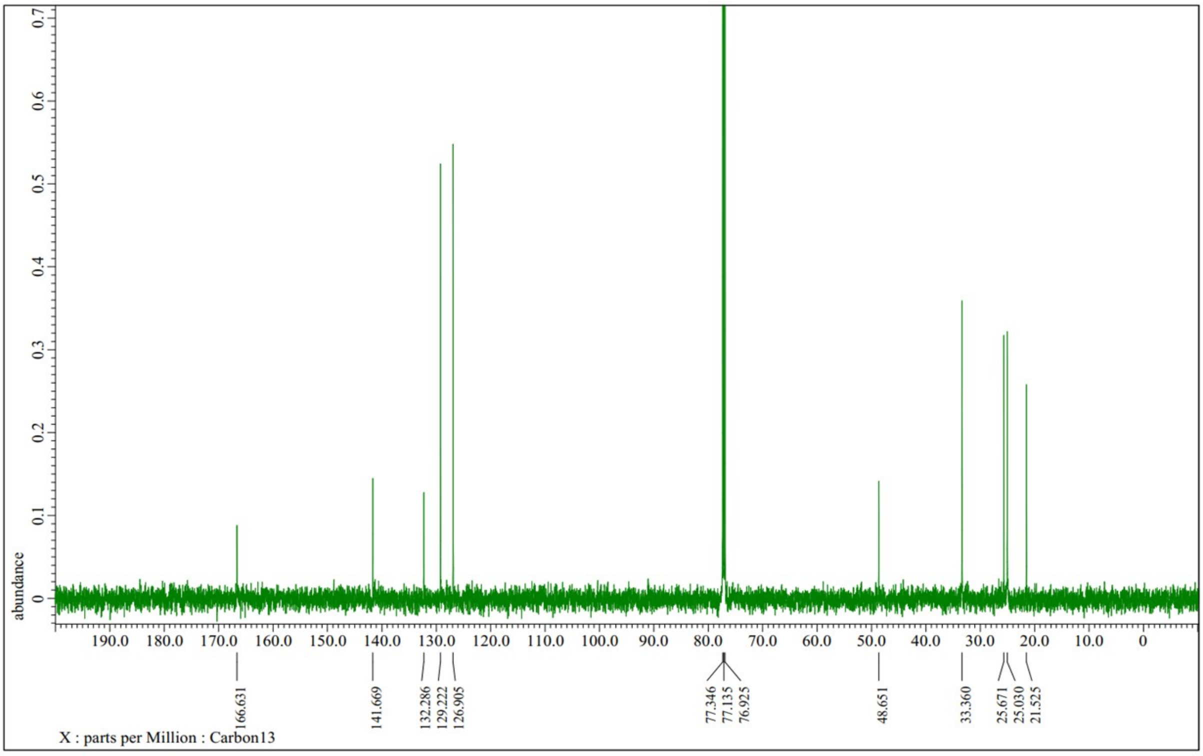

^{13}C NMR spectrum of *N*-cyclohexyl-4-methylbenzamide (3az)

4-chloro-*N*-cyclohexylbenzamide (3ba)

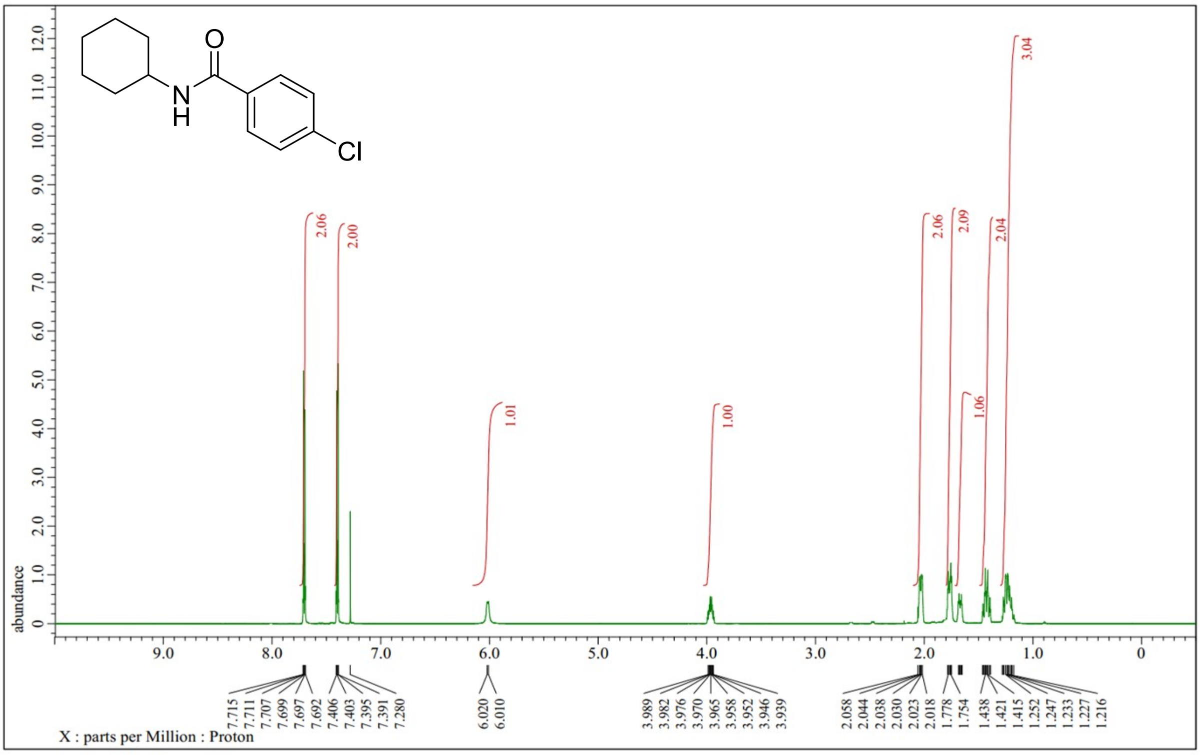

^{1}H NMR spectrum of 4-chloro-*N*-cyclohexylbenzamide (3ba)

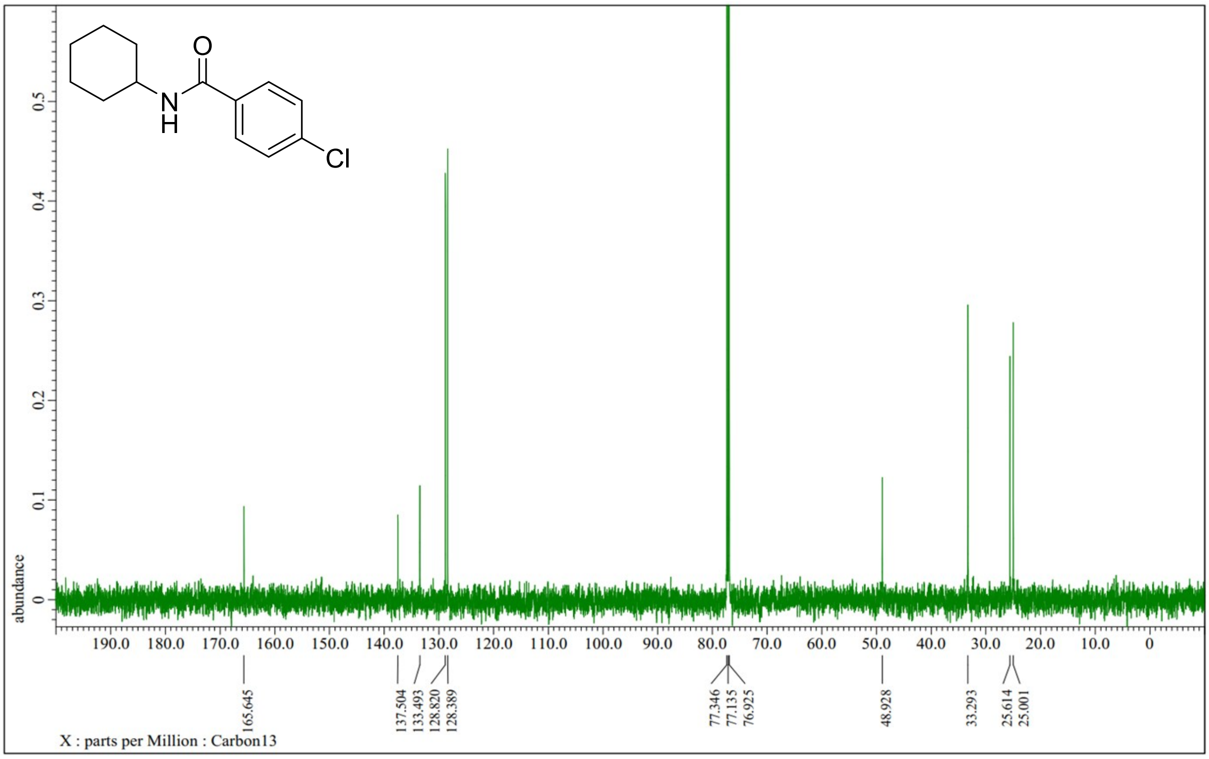

^{13}C NMR spectrum of 4-chloro-*N*-cyclohexylbenzamide (3ba)
